# Design and synthesis of bioactive Ru(ii) complexes: antibacterial activity, biocompatibility and biomolecular binding

**DOI:** 10.1039/d5ra05336f

**Published:** 2025-11-04

**Authors:** Debasis Bhunya, Riya Datta, Ribhu Maity, Alipe Saha, Sujata Sen, Paula Brandao, Satyajit Pattanayak, Tithi Maity, Keka Sarkar, Bidhan Chandra Samanta

**Affiliations:** a Department of Chemistry, Christ University Hosur Road Bengaluru-560029 Karnataka India riya.datta@christuniversity.in; b Department of Chemistry, Mugberia Gangadhar Mahavidyalaya Bhupatinagar Purba Medinipur-721425 West Bengal India bidhansamanta@yahoo.in; c Department of Microbiology, University of Kalyani West Bengal India; d Departamento de Química, CICECO, Universidade de Aveiro 3810-193 Aveiro Portugal; e Department of Chemistry, Prabhat Kumar College Purba Medinipur-721401 Contai, West Bengal India

## Abstract

Ruthenium(ii) complexes with N- and S-donor ligands have emerged as promising alternatives to conventional antibiotics due to their stability, biocompatibility, and ability to interact with biological macromolecules. In this work, a series of four Ru(ii)–thiazolidine complexes, [Ru(ii)(L1–L4)(*p*-cymene)Cl]PF_6_, were synthesized and structurally characterized using spectroscopic techniques and X-ray crystallography. Their interactions with DNA and proteins showed partial groove binding with calf thymus DNA and a static quenching mechanism with bovine serum albumin (BSA). Biological investigations revealed that two of the complexes exhibited strong antioxidant activity and significant antibacterial effects against methicillin-resistant *Staphylococcus aureus* (MRSA) and *Klebsiella pneumonia* (KP). Moreover, hemolysis assays confirmed their favourable biocompatibility. These results highlight Ru(ii)–thiazolidine frameworks as promising candidates for antimicrobial drug development. This study not only underscores their therapeutic potential but also advances the role of ruthenium-based coordination chemistry in addressing the persistent challenge of antibiotic resistance.

## Abstract 2

Ruthenium(ii) complexes with N- and S-donor ligands have emerged as promising alternatives to conventional antibiotics due to their stability, biocompatibility, and ability to interact with biological macromolecules.

## Graphical abstract

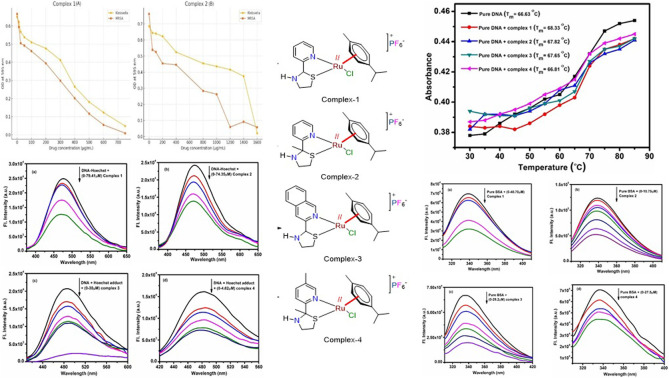

## 1. Introduction

The strategy to develop organometallic complexes with novel mechanisms of action against drug-resistant pathogens has now become a rapidly growing area of research.^1–5^ Among the transition metals, Ru(ii) complexes have attracted significant interest due to their convenient redox chemistry, low toxicity, and ability to avoid classical resistance mechanisms.^6–8^ The worldwide growth of antibiotic-resistant infections raises a serious healthcare challenge, mainly with pathogens such as MRSA and KP. MRSA is notorious for its resistance to β-lactam antibiotics through acquisition of the *mecA* gene, which encodes the low-affinity penicillin-binding protein PBP2a.^9,10^ Similarly, KP has emerged as a major cause of nosocomial infections, due to its ability to produce extended-spectrum β-lactamases (ESBLs), carbapenemases, and express multidrug efflux systems such as, AcrA (Acriflavine resistance protein A), AcrB (Acriflavine resistance protein B), TolC (Outer membrane protein TolC) and porin mutations.^11–13^

Contrasting traditional antibiotics, Ru(ii) complexes can act *via* manifold mechanisms, including DNA intercalation, and reactive oxygen species (ROS) generation, making them effective even against multidrug-resistant strains.^14–17^ These properties make Ru(ii) systems attractive platforms for the development of multifunctional bioactive compounds. Matshwele *et al.* (2021)^18^ reported that Ru(II/III) polypyridyl complexes with pyridyl moieties exhibited strong antibacterial activity against both drug-sensitive and resistant strains, including *Staphylococcus aureus*, MRSA and KP. Dinuclear Ru(ii) complexes bearing 2-picolyl-polypyridyl ligands effectively inhibited MRSA and non-resistant *K. pneumoniae*, with some outperforming ciprofloxacin and chloramphenicol. Though inactive against multidrug-resistant KPPP, they showed potent DNA denaturation, indicating DNA cleavage as the likely mechanism. The Ru center was essential for activity, as free ligands were inactive, highlighting Ru(ii) complexes as promising candidates against resistant bacterial infections.^19,20^ However, these studies primarily focused on polypyridyl or simple Ru scaffolds, leaving a gap in exploring ligand systems that combine nitrogen and sulphur donors.

In this context, thiazolidine-based ligands introduce a distinctive sulphur and nitrogen donor (S/N) environment and offer an underexplored but promising platform. Their dual (S/N) donor set provides enhanced chelation, which can stabilize the Ru(ii) center and potentially tune redox properties, lipophilicity. The sulphur donor, as a soft base, provides strong binding to the soft Ru(ii) center, improving kinetic stability and lipophilicity, which may facilitate membrane permeability. The nitrogen donor contributes additional electronic modulation, favouring DNA/protein binding and fine-tuning redox properties. Together, this cooperative S/N coordination can yield complexes that are not only stable in biological environments but also capable of interacting more effectively with biomolecular targets.^21–23^ Thus, these structural variations in thiazolidine ligands influence DNA/protein binding, antioxidant behaviour, and bacterial uptake, which translates into therapeutic implications (potency, selectivity, safety). Incorporating these ligands into the well-established Ru(ii)–*p*-cymene framework creates an opportunity to systematically probe how subtle structural variations influence biological activity. This structure–activity relationship (SAR) perspective is particularly important for identifying design features that translate into therapeutic potential.^24–28^

Here, we report the synthesis and characterization of Ru(ii)–thiazolidine complexes and their biological evaluation against MRSA and KP. By integrating DNA/protein binding, antioxidant assays, and antibacterial studies, we establish correlations between structural features and functional outcomes. To our knowledge, this is among the first studies to investigate Ru(ii)–thiazolidine systems against clinically relevant multidrug-resistant pathogens, highlighting the promise of S/N-donor scaffolds in antimicrobial drug development.

## 2. Experimental

### 2.1 Materials

The present experiments utilized materials as purchased without any further purification. Picolinaldehyde (99%, Sigma-Aldrich), aminoethane-1-thiol hydrochloride, 1-(pyridin-2-yl)ethan-1-one, methanol (≥99.8%, Sigma-Aldrich), [Ru ii(*p*-cymene)Cl_2_]_2_, ammonium hexafluorophosphate (99.98%, Sigma-Aldrich), K_2_CO_3_ (99.99%, Sigma-Aldrich), Hoechst (≥98.0%, Sigma-Aldrich), ethidium bromide (95%, Sigma-Aldrich) and DPPH (2,2-diphenyl-1-picrylhydrazyl) (≥90%, Sigma-Aldrich) were used for this present study. BSA and DNA were obtained from Sigma-Aldrich Chemicals Private Limited and prepared in a 0.1 M HEPES (4-(2-hydroxyethyl)-1-piperazineethanesulfonic acid) buffer with a pH of 7.4. A 0.1 M HEPES buffer solution contains 0.1 M of HEPES acid and 0.1 M HEPES sodium salt. The ionic strength of this buffer solution is approximately 0.172 M.

### 2.2 Synthesis of ligands

#### 2.2.1 2-(Pyridin-2-yl)thiazolidine (L1)

**Scheme 1:**
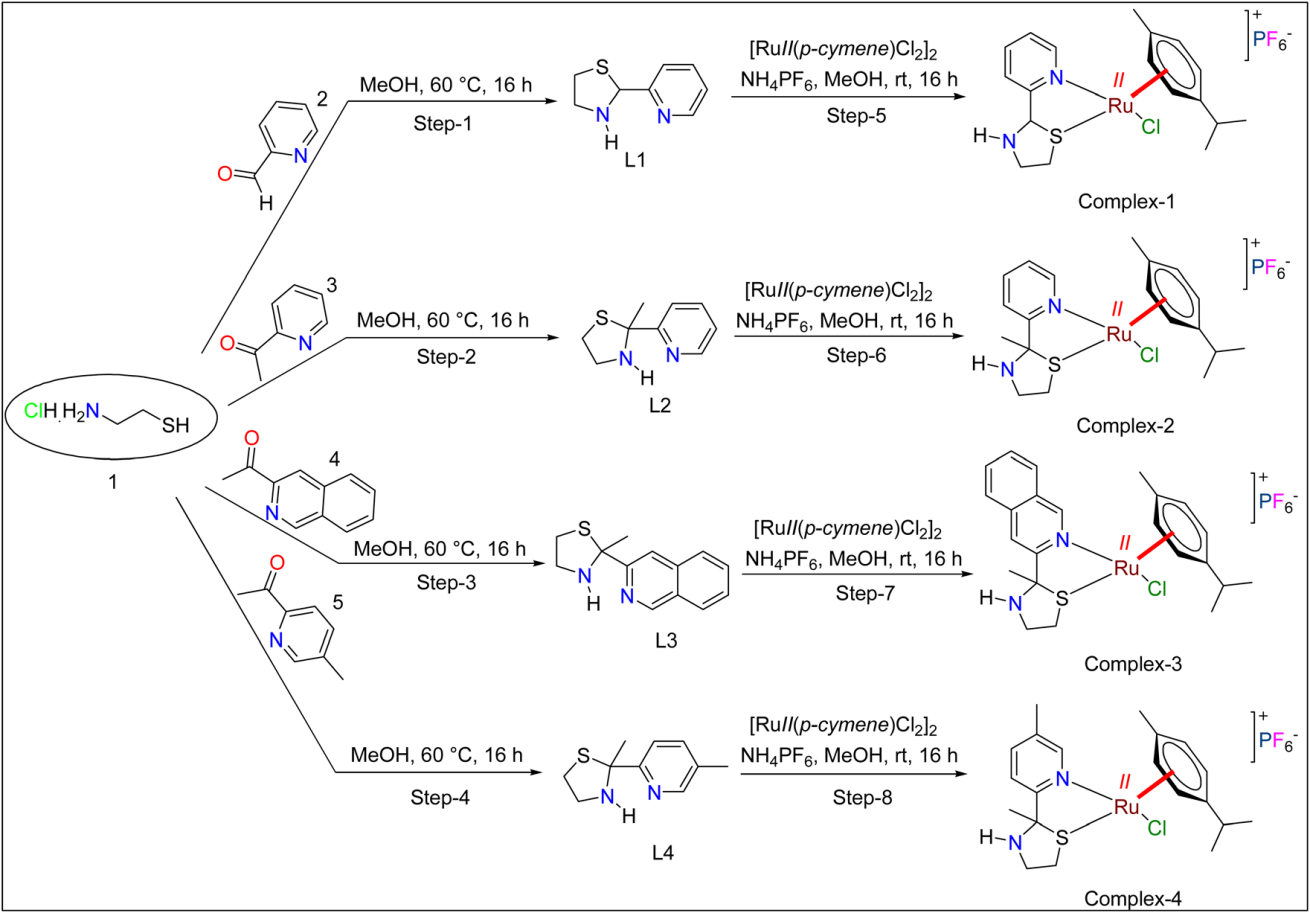
Preparation route of ligands and complexes.

A mixture of picolinaldehyde (1 g, 9.33 mmol) and 2-aminoethane-1-thiol hydrochloride (1.06 g, 9.33 mmol) in methanol (20 mL) was stirred at 60 °C for 16 h. After completion (TLC), the solvent was removed under reduced pressure, and the residue was diluted with water (30 mL) and extracted with ethyl acetate. The combined organic layers were washed with saturated NaHCO_3_ solution and brine, dried over anhydrous Na_2_SO_4_, and concentrated to give crude 2-(pyridin-2-yl)thiazolidine (600 mg) as a sticky liquid (Scheme 1).

##### 2.2.1.1 ESI-LCMS (methanol) *m*/*z* (exp.)

167.09 (167.06) [C_8_H_11_N_2_S (M + H)]. ^1^H NMR (400 MHz, CDCl_3_) δ 8.60 (d, *J* = 0.8 Hz, ^1^H), 7.64 (t, *J* = 3.8 Hz, ^1^H), 7.31 (d, *J* = 8.0 Hz, ^1^H), 7.20 (t, *J* = 2.4 Hz, ^1^H), 5.88 (s, ^1^H), 3.83–3.79 (m, ^1^H), 3.18–3.04 (m, 4H). ^13^C NMR (100 MHz, CDCl_3_): δ 158.31, 150.00, 136.91, 123.23, 122.22, 72.91, 53.65, 37.17.

#### 2.2.2 2-Methyl-2-(pyridin-2-yl)thiazolidine (L2)

1-(Pyridin-2-yl)ethan-1-one (1 g, 8.25 mmol) and 2-aminoethane-1-thiol hydrochloride (1.13 g, 9.91 mmol) were stirred in methanol at 60 °C for 16 h. Then, the mixture was concentrated under reduced pressure, diluted with water (30 mL), and extracted with ethyl acetate (2 × 20 mL). The organic layer was washed with saturated NaHCO_3_ and brine, dried over anhydrous Na_2_SO_4_, and concentrated to yield 2-methyl-2-(pyridin-2-yl)thiazolidine (600 mg) as a sticky liquid (Scheme 1).

##### 2.2.2.1 ESI-LCMS (methanol) *m*/*z* (exp.)

181.19 (181.07) [C_9_H_13_N_2_S (M + H)]. ^1^H NMR (400 MHz, CDCl_3_) δ 8.55 (d, *J* = 0.8 Hz, ^1^H), 7.64 (t, *J* = 3.8 Hz, ^1^H), 7.54 (d, *J* = 8.0 Hz, ^1^H), 7.14 (t, *J* = 2.4 Hz, ^1^H), 3.50–3.40 (m, 2H), 3.17–3.11 (m, ^1^H), 3.08–3.03 (m, ^1^H), 1.95 (s, 3H). ^13^C NMR (100 MHz, CDCl_3_) δ 164.01, 149.02, 136.96, 122.25, 119.24, 81.05, 52.42, 38.61, 31.25.

#### 2.2.3 2-(Isoquinolin-3-yl)-2-methylthiazolidine (L3)

A mixture of 1-(isoquinolin-3-yl)ethan-1-one (1 g, 5.84 mmol) and 2-aminoethane-1-thiol hydrochloride (536.6 mg, 5.84 mmol) in methanol (20 mL) was stirred at 60 °C for 16 h. After that, the mixture was concentrated, diluted with water (30 mL), and extracted with ethyl acetate (2 × 20 mL). The organic layer was washed with saturated NaHCO_3_ and brine, dried over anhydrous Na_2_SO_4_, and concentrated to give 2-(isoquinolin-3-yl)-2-methylthiazolidine (500 mg, crude) as a sticky liquid (Scheme 1).

##### 2.2.3.1 ESI-LCMS (methanol) *m*/*z* (exp.)

231.19 (231.09) [C_13_H_15_N_2_S (M + H)]. ^1^H NMR (400 MHz, CDCl_3_) δ 9.24 (s, ^1^H), 7.95 (d, *J* = 8.0 Hz, ^1^H), 7.87 (s, ^1^H), 7.82 (d, *J* = 8.0 Hz, ^1^H), 7.66 (t, *J* = 4.2 Hz, ^1^H), 7.55 (t, *J* = 4.0 Hz, ^1^H), 3.58–3.46 (m, 2H), 3.32–3.28 (m, ^1^H), 3.20–3.12 (m, 2H), 2.06 (s, 3H). ^13^C NMR (100 MHz, CDCl_3_): δ 157.34, 152.51, 131.21, 130.77, 127.89, 127.71, 127.29, 120.50, 114.56, 81.66, 52.55, 38.62, 31.14, 14.41.

#### 2.2.4 2-Methyl-2-(5-methylpyridin-2-yl)thiazolidine (L4)

A mixture of 1-(5-methylpyridin-2-yl)ethan-1-one (1 g, 7.40 mmol) and 2-aminoethane-1-thiol hydrochloride (840.5 mg, 7.40 mmol) in methanol (20 mL) was stirred at 60 °C for 16 h. Then, the mixture was concentrated, diluted with water (30 mL), and extracted with ethyl acetate (2 × 20 mL). The organic layer was washed with saturated NaHCO_3_ and brine, dried over anhydrous Na_2_SO_4_, and concentrated to yield 2-methyl-2-(5-methylpyridin-2-yl)thiazolidine (600 mg, crude) as a sticky liquid (Scheme 1).

##### 2.2.4.1 ESI-LCMS (methanol) *m*/*z* (exp.)

195 (195.09) [C_10_H_15_N_2_S (M + H)]. ^1^H NMR (400 MHz, CDCl_3_) δ 8.38 (s, ^1^H), 7.46 (d, *J* = 1.6 Hz, ^1^H), 7.42 (d, *J* = 7.6 Hz, ^1^H), 3.52–3.38 (m, 3H), 3.16–3.11 (m, ^1^H), 3.08–3.02 (m, ^1^H), 2.32 (s, 3H), 1.93 (s, 3H). ^13^C NMR (100 MHz, CDCl_3_): δ 161.08, 149.39, 137.49, 131.74, 118.80, 80.91, 52.41, 38.60, 31.23, 18.27.

### 2.3 Synthesis of the complexes

#### 2.3.1 [Ruii(L1)(*p*-cymene)Cl]PF_6_ (complex 1)

2-(Pyridin-2-yl)thiazolidine (200 mg, 1.20 mmol) and [Ruii(*p*-cymene)Cl_2_]_2_ (373 mg, 0.60 mmol) were stirred in methanol (30 mL) at room temperature for 16 h. After adding ammonium hexafluorophosphate (196 mg, 1.20 mmol) and stirring for 1 h, the mixture was concentrated and purified by silica gel chromatography (MeOH/DCM, 0–3%) to yield [Ruii(L1)(*p*-cymene)Cl]PF_6_ as a brown solid (130 mg, 23%) (Scheme 1). Crystallization attempts failed.

##### 2.3.1.1 ESI-LCMS (methanol) *m*/*z* (exp.)

437.24 (437.04) [C_18_H_24_ClN_2_SRu^+^], Anal. Calcd. for C_18_H_24_ClN_2_SRuPF_6_: C, 37.15; H, 4.16; N, 4.81. Found: C, 36.679; H, 4.149; N, 4.8522. ^1^H NMR (400 MHz, CDCl_3_) δ 8.94 (d, *J* = 0.8 Hz, ^1^H), 7.89 (t, *J* = 3.8 Hz, ^1^H), 7.64 (d, *J* = 8.0 Hz, ^1^H), 7.44 (t, *J* = 6.2 Hz, ^1^H), 6.51 (d, *J* = 7.6 Hz, ^1^H), 5.93 (d, *J* = 6.0 Hz, ^1^H), 5.82 (q, *J* = 4.8 Hz, 2H), 5.70 (d, *J* = 6.0 Hz, ^1^H), 3.59–3.47 (m, 2H), 3.20–3.18 (m, ^1^H), 2.96–2.93 (m, ^1^H), 2.68–2.61 (m, ^1^H), 2.30–2.39 (m, ^1^H), 2.16 (s, 3H), 1.33 (d, *J* = 6.8 Hz, 3H), 1.28 (d, *J* = 6.8 Hz, 3H). ^13^C NMR (100 MHz, CDCl_3_): δ 155.81, 139.88, 126.50, 125.85, 90.26, 87.18, 86.45, 85.99, 83.95, 51.25, 33.78, 30.88, 22.76, 22.15, 18.03. ^19^F NMR (400 MHz, CDCl_3_) δ −70.15 (d, *J* = 756 Hz). FTIR (KBr pellets, cm^−1^): 3369 (N–H).

#### 2.3.2 [Ruii(L2)(*p*-cymene)Cl]PF_6_ (complex 2)

2-Methyl-2-(pyridin-2-yl)thiazolidine (200 mg, 1.11 mmol) and [Ruii(*p*-cymene)Cl_2_]_2_ (344 mg, 0.55 mmol) were stirred in methanol (30 mL) at room temperature for 16 h. After adding ammonium hexafluorophosphate (181 mg, 1.11 mmol) and stirring for 1 h, the mixture was concentrated and purified by silica gel chromatography (MeOH/DCM, 0–3%) to yield [Ruii(L2)(*p*-cymene)Cl]PF_6_ as a brown solid (200 mg, 40%) (Scheme 1). Single crystals suitable for XRD were obtained from acetonitrile–diethyl ether after 2–3 days.

##### 2.3.2.1 ESI-LCMS (methanol) *m*/*z* (exp.)

451.54 (451.05) [C_19_H_26_ClN_2_SRu^+^], Anal. Calcd for C_19_H_26_ClN_2_SRuPF_6_: C, 38.29; H, 4.40; N, 4.70. Found: C, 38.194; H, 4.087; N, 4.3299. ^1^H NMR (400 MHz, CDCl_3_) δ 8.94 (d, *J* = 5.2 Hz, ^1^H), 7.89 (t, *J* = 7.2 Hz, ^1^H), 7.51 (d, *J* = 7.6 Hz, ^1^H), 7.43 (t, *J* = 6.0 Hz, ^1^H), 5.99 (d, *J* = 5.6 Hz, ^1^H), 5.80 (t, *J* = 6.4 Hz, 2H), 5.73 (d, *J* = 6.0 Hz, ^1^H), 3.60 (q, *J* = 5.7 Hz, 2H), 3.16 (d, *J* = 8.4 Hz, ^1^H), 2.99–2.93 (m, ^1^H), 2.81–2.74 (m, ^1^H), 2.17–2.22 (m, ^1^H), 2.14 (s, 3H), 1.98 (s, 3H), 1.35 (d, *J* = 7.2 Hz, 3H), 1.29 (d, *J* = 6.8 Hz, 3H). ^13^C NMR (100 MHz, CDCl_3_): δ 165.43, 156.64, 140.66, 126.77, 124.80, 109.88, 102.28, 96.51, 91.60, 88.39, 85.93, 85.25, 52.32, 35.58, 31.24, 27.11, 22.91, 22.26, 18.77. ^19^F NMR (400 MHz, CDCl_3_) δ −70.13 (d, *J* = 756 Hz). FTIR (KBr pellets, cm^−1^): 3355 (N–H).

#### 2.3.3 [Ruii(L3)(*p*-cymene)Cl]PF_6_ (complex 3)

2-(Isoquinolin-3-yl)-2-methylthiazolidine (200 mg, 0.87 mmol) and [Ruii(*p*-cymene)Cl_2_]_2_ (269 mg, 0.43 mmol) were stirred in methanol (30 mL) at room temperature for 16 h. After adding ammonium hexafluorophosphate (142 mg, 0.87 mmol) and stirring for 1 h, the mixture was concentrated and purified by silica gel chromatography (MeOH/DCM, 0–3%) to yield [Ruii(L3)(*p*-cymene)Cl]PF_6_ as a brown solid (150 mg, 36%) (Scheme 1). Crystallization attempts were unsuccessful.

##### 2.3.3.1 ESI-LCMS (methanol) *m*/*z* (exp.)

501.24 (501.07) [C_23_H_28_ClN_2_SRu^+^], Anal. Calcd for C_19_H_26_ClN_2_SRuPF_6_: C, 42.76; H, 4.37; N, 4.34. Found: C, 43.147; H, 4.199; N, 4.271. ^1^H NMR (400 MHz, CDCl_3_) δ 9.73 (s, ^1^H), 8.19 (d, *J* = 8.0 Hz, ^1^H), 7.89–7.82 (m, 3H), 7.73 (t, *J* = 3.4 Hz, ^1^H), 6.09 (d, *J* = 1.2 Hz, ^1^H), 5.93 (d, *J* = 5.6 Hz, ^1^H), 5.88 (d, *J* = 5.2 Hz, ^1^H), 5.79 (d, *J* = 6.0 Hz, ^1^H), 3.65–3.59 (m, 2H), 3.20–3.16 (m, ^1^H), 3.00–2.93 (m, ^1^H), 2.81–2.76 (m, ^1^H), 2.39–2.34 (m, ^1^H), 2.12 (s, 3H), 2.02 (s, 3H), 1.37 (d, *J* = 6.8 Hz, 3H), 1.28 (d, *J* = 3.6 Hz, 3H). ^13^C NMR (100 MHz, CDCl_3_): δ 160.61, 156.34, 137.12, 133.99, 129.90, 129.82, 128.63, 126.86, 121.60, 109.44, 102.42, 95.34, 92.11, 88.49, 86.01, 85.30, 52.06, 35.51, 31.25, 28.24, 23.04, 22.11, 18.36. ^19^F NMR (400 MHz, CDCl_3_) δ −70.13 (d, *J* = 756 Hz). FTIR (KBr pellets, cm^−1^): 3349 (N–H).

#### 2.3.4 [Ruii(L4)(*p*-cymene)Cl]PF_6_ (complex 4)

2-Methyl-2-(5-methylpyridin-2-yl)thiazolidine (200 mg, 1.03 mmol) and [Ruii(*p*-cymene)Cl_2_]_2_ (319 mg, 0.51 mmol) were stirred in methanol (30 mL) at room temperature for 16 h. After adding ammonium hexafluorophosphate (168 mg, 1.03 mmol) and stirring for 1 h, the mixture was concentrated and purified by silica gel chromatography (MeOH/DCM, 0–3%) to yield [Ruii(L4)(*p*-cymene)Cl]PF_6_ as a brown solid (150 mg, 36%) (Scheme 1). Single crystals formed from acetonitrile–diethyl ether after 2–3 days, but diffraction data could not be collected.

##### 2.3.4.1 ESI-LCMS (methanol) *m*/*z* (exp.)

464.79 (465.07) [C_20_H_28_ClN_2_SRu^+^], Anal. Calcd for C_19_H_26_ClN_2_SRuPF_6_: C, 39.38; H, 4.63; N, 4.59. Found: C, 39.774; H, 4.375; N, 4.569. ^1^H NMR (400 MHz, CDCl_3_) δ 8.73 (s, ^1^H), 7.69 (d, *J* = 1.2 Hz, ^1^H), 7.37 (d, *J* = 8.0 Hz, ^1^H), 5.98 (d, *J* = 0.8 Hz, ^1^H), 5.83 (d, *J* = 6.4 Hz, ^1^H), 5.79 (d, *J* = 1.2 Hz, ^1^H), 5.71 (d, *J* = 6.0 Hz, ^1^H), 3.61–3.56 (m, 2H), 3.14–3.10 (m, ^1^H), 2.99–2.92 (m, ^1^H), 2.79–2.72 (m, ^1^H), 2.42 (s, 3H), 2.18–2.16 (m, ^1^H), 2.13 (s, 3H), 1.95 (s, 3H), 1.36 (d, *J* = 7.2 Hz, 3H), 1.28 (d, *J* = 6.8 Hz, 3H). ^13^C NMR (100 MHz, CDCl_3_): δ 162.49, 156.19, 141.52, 137.30, 124.00, 109.87, 102.11, 96.30, 91.54, 88.31, 85.86, 85.15, 52.20, 35.50, 31.21, 27.11, 22.81, 22.25, 18.34, 18.24. ^19^F NMR (400 MHz, CDCl_3_) δ −70.13 (d, *J* = 756 Hz). FTIR (KBr pellets, cm^−1^): 3356 (N–H).

## 3. Characterization

### 3.1 Crystal structure determination

Single-crystal X-ray diffraction was employed to determine the geometric and electronic structure for the complex 2 only. The X-ray single crystal data was collected with monochromatic Mo-Kα radiation (*λ* = 0.71073 Å) on a Bruker D8 Venture diffractometer equipped with a photon detector 100 CMOS at 293 (2) K. Data reduction was carried out using the SAINT-NT software package. Multi-scan absorption correction was applied to all intensity data using the SADABS program. The structure was refined *via* full matrix least squares on F^2^ using the SHELX-2013 suite.^29^ All non-hydrogen atoms were refined with anisotropic thermal displacements. The C–H hydrogen atoms were included at calculated positions and refined with isotropic parameters equivalent to 1.2 times those of the atom to which they are attached. The hydrogen bonded to N2 atom was located on the difference Fourier map. Molecular diagrams were drawn with Mercury software.^30^ The crystal data and selected refinement details are listed in Table S1. The CCDC number for complex 2 is 2376098, as obtained from the submission of CIF files to the Cambridge Crystallographic Data Centre.

### 3.2 Spectroscopic analysis

Elemental analyses was carried out using Elemental Analyser – CHNSO (Model: UNICUBE; Make: Elementar Analysensysteme GmbH, Germany). ^1^H and ^13^C NMR spectra were obtained using a Bruker Avance 400 MHz spectrometer at room temperature. Mass spectra were measured by ESI-LCMS (Electrospray Ionization Liquid Chromatography Mass Spectrometry) and were recorded on a Finnigan MAT 95. A Systronic 2202 UV-vis spectrophotometer (manufactured by Systronic, India) was employed for UV studies.

### 3.3 Lipophilicity test

The flask-shaking method was used to assess the lipophilicity of the complexes, expressed as the partition coefficient between *n*-octanol and water (Log *P*_o/w_).^31^ For this purpose, 0.001 g of each complex was added to a 10 mL mixture of *n*-octanol and water (1 : 1 v/v) at 298 K. The concentration of the complex in each phase was determined using the Beer–Lambert law, and the lipophilicity was calculated using the formula 1:

1 Log *P*_o/w_ = log([complex]_octanol phase_/[complex]_water phase_)

### 3.4 Antioxidant studies

To evaluate the potential antioxidant properties of the complexes, the DPPH (2,2-diphenyl-1-picrylhydrazyl) free radical scavenging assay was performed using a well-established method.^32^ For this analysis, dissolving the complex in a 1 : 1 mixture of DMSO and water, the working solution was prepared. A 75 μM DPPH solution was prepared in methanol. Varying concentrations of the complex solutions in methanol were then added to 5 mL of the DPPH solution. The resulting mixture was vigorously shaken and incubated at 30 °C in the dark for 30 minutes. The reduction in absorbance of DPPH at 517 nm was measured for the complexes, using ascorbic acid (AA) as a standard. The colour change of the pure DPPH solution was also observed with the gradual addition of the complexes. The percentage of DPPH scavenging activity was calculated using the following formula 2:

2 Scavenging activity (%) = [(*A*_0_ − *A*_1_)/*A*_0_] × 100

where *A*_0_ and *A*_1_ are the absorbance of pure DPPH in absence and presence of an oxidant respectively. The IC_50_ was determined by means of percentage of activity.

### 3.5 DNA binding interaction studies

The interaction with DNA was examined using a UV-vis spectrophotometer (Systronic India) by incrementally adding CT-DNA to a fixed concentration of each complex in a reference buffer solution.^31^ Emission studies were conducted on a Shimadzu RF-6000 Spectrofluorophotometer, covering the wavelength range of 200 to 700 nm.^32^

### 3.6 BSA interaction study

The interaction study with BSA was monitored using absorption and emission spectroscopy techniques. In the UV-vis study, changes in the absorption for a fixed concentration of each complex were observed with a gradual increase of BSA concentration. Meanwhile, in the fluorescence study, variations at 335 nm were recorded with a gradual increase in each complex concentration.^31,32^

### 3.7 Antibacterial activity assays

Various assays were performed to investigate the antibacterial efficacy of complex 1, complex 2, complex 3 and complex 4 using methicillin-resistant *Staphylococcus aureus* (ATCC700699; MRSA) and a drug-resistant strain of *Klebsiella pneumoniae* (BAA1705; KP) as the model organisms.^33^

#### 3.7.1 Cup plate assay

The test microorganisms were qualitatively assessed for susceptibility to complex 1, complex 2, complex 3 and complex 4 as compared to a standard prescribed antibiotic *via* a cup plate assay. The turbidness of an overnight bacterial culture of KP and MRSA was calibrated with Nutrient Broth (NB) to achieve an optical density of 0.5 on the McFarland scale, corresponding to 10^7^ CFU mL^−1^, and 100 μL of bacterial inoculum was inoculated onto a nutrient agar (NA) plate using a sterile glass spreader. Next, wells were punctured into the solid medium using a sterile cork borer to incorporate 100 μL of each test compound dissolved in 1 percent DMSO solution with concentration of 5 mg mL^−1^ respectively. To compare the antibacterial effect of the complexes, a positive control well was administered with 100 μL of commercially purchased cefoxitin (30 μg mL^−1^). Additionally, 100 μL of 1% DMSO solution was added to a well as negative control. Following a 24 h incubation at 37 °C, the diameters of the inhibition zones were measured using a caliper. The experiment was replicated using MRSA as test organism.^33^

#### 3.7.2 MIC and MBC analyses

In order to determine the MIC values of the complexes, 96-well microtiter plates (Himedia) were used. NB was aliquoted (200 μL) into the wells and inoculated with 2 μL of overnight grown bacterial suspension (MRSA or KP into designated wells). Next, test concentrations of the complexes (Complex 1: 750, 600, 500, 400, 300, 200, 100, 50, 25, and 12.5 μg mL; Complex 2: 1600, 1400, 1200, 1000, and 800 μg mL^−1^) were prepared and incorporated into the designated wells. A positive control set was established using inoculum-seeded NB without any treatment. NB alone was added to a set of wells to prepare a negative control. An additional set of wells were furnished with the complexes in the range of the test concentrations to calibrate the O.D.s from these complexes alone. Subsequently, the plates were subjected to shaking incubation at 37 °C, 160 rpm for 24 h. Bacterial growth was determined through measuring O.D. at 595 nm using an ELISA reader (Bio-Rad iMarkTM), and the lowest concentration of these complexes where no bacterial growth was seen was recorded as the MIC values.

MBC depicts the lowest concentration of an antimicrobial agent where no microbial colonies appeared as compared to its corresponding MIC. MBC assay of complex 1 and complex 2 was conducted by seeding inoculum from the replica wells of both KP and MRSA sets from the prior MIC experiment onto NA plates. Aliquots from the positive controls of both sets were plated to establish baseline concentrations of the test microorganisms, and the plates were incubated for 24 h at 37 °C.^33^

#### 3.7.3 SEM studies

Fresh overnight cultures of MRSA and KP were diluted with NB in a 1 : 10 ratio to attain bacterial suspensions containing approximately 10^7^ CFU mL^−1^. Subsequently, 1% DMSO solutions of the test compounds were incorporated into the diluted suspensions: complex 1 (250 μg mL^−1^ for both MRSA and KP) and complex 2 (600 and 800 μg mL^−1^ for MRSA and KP, respectively). The treated and untreated (control) bacterial suspensions were incubated at 160 rpm and 37 °C for 24 h, following which, 1 mL aliquots of each sample were transferred to 1.5 mL microcentrifuge tubes and centrifuged at 10 000 rpm for 5 min. The resultant pellets were rinsed thrice with 1× PBS (phosphate buffered saline) to eliminate any residual impurities and dissolved in 100 μL of 1× PBS. Next, 3 mL of the suspensions were applied to 12-mm round cover slips and permitted to air dry. The bacterial cells were immobilized on the cover slips with 10 mL of 2.5% glutaraldehyde, and the cover slips were incubated overnight in the dark at 4 °C. Post-incubation, the cover slips were washed with graded ethanol (concentrations ranging from 30% to 100%) to dehydrate the samples and then stored in a desiccator until microscopic imaging.^33^

#### 3.7.4 Hemolysis assay

In order to assess the biocompatibility of complex 1 and complex 2 with blood, a hemolysis assay was conducted using sheep blood. Heparin-stabilized fresh blood was centrifuged at 5000 rpm for 10 min at room temperature. The supernatant was discarded, the pellet resuspended in freshly prepared PBS, and centrifuged under the aforementioned conditions; this process was repeated three times to purify the erythrocytes. Subsequently, the pellet was diluted with PBS and the resultant solution was treated with three distinct concentrations of complex 1 and complex 2 – half MIC (Complex 1: 250 μg mL^−1^, complex 2: 800 μg mL^−1^), MIC (Complex 1: 500 μg mL^−1^, complex 2: 1600 μg mL^−1^), and double MIC (complex 1: 1000 μg mL^−1^, complex 2: 3200 μg mL^−1^), while maintaining the final volume at 1 mL. PBS- and Triton X-100-treated cells were used as negative and positive controls, respectively, and all the samples were incubated at 37 °C for four hours. Following incubation, the samples were centrifuged for 10 min at 10 000 g and monitored for occurrence of hemolysis.^33^

#### 3.7.5 Analytical statistics

Every experiment was carried out three times and then independently. A mean ± standard deviation (0.05) derived from three separate replicates is used to present the data.

## 4. Results and discussion

### 4.1 Spectroscopic analyses of both the ligands and the complexes

Scheme 1 illustrates the synthetic route for the successful preparation of ligands and the complexes. The structures were confirmed through comprehensive spectroscopic and analytical characterization including ^1^H NMR, ^13^C NMR and mass spectrometry for all the four ligands and elemental analysis, ^1^H NMR, ^13^C NMR, ^19^F NMR and mass spectrometry for all the four complexes.

The results obtained from elemental analyses of the complexes reveals good agreement of them with the theoretical values. Comparison of the ^1^H NMR spectra of the ligands (Fig. S1–S4) and corresponding complexes (Fig. S13–S16) exposes indicative chemical shift changes consistent with complex formation. For example, the pyridyl proton in ligand L1 appears at δ 8.60 ppm, which shifts downfield to δ 8.95 ppm in complex 1, indicating coordination of the nitrogen donor to the ruthenium center. Similarly, for complex 2, a doublet at δ 8.94 ppm (pyridyl-H) and multiplets at δ 7.89–7.43 ppm confirm aromatic coordination. The η^6^-bound *p*-cymene moiety in all complexes shows characteristic aromatic proton signals between δ 5.68–6.10 ppm, while the isopropyl methyl groups resonate as doublets near δ 1.14–1.35 ppm. Methyl groups on ligand scaffolds are also preserved, with signals such as δ 2.16 (CH_3_, complex 1), δ 2.14 and 1.98 (CH_3_, complex 2), δ 2.02 (CH_3_, complex 3) and δ 2.42, 2.12 and 1.95 (CH_3_, complex 4).

The ^13^C NMR spectra of ligands (Fig. S5–S8) and the complexes (Fig. S17–S20) further support complex formation from the ligands. In complex 1, characteristic downfield carbon signals are observed at δ 155.81 (C=N), 139.88, and 126.50 ppm for the pyridyl-thiazolidine framework, while the arene carbons of the *p*-cymene ligand resonate between δ 83.9–90.0 ppm. Aliphatic carbons from the thiazolidine ring appear at δ 30.2–50.8 ppm. Similar patterns are observed in complex 2, with δ 165.4, 156.8 (aromatic C), and δ 85.5–96.5 (*p*-cymene), while methyl and methylene carbons resonate at δ 22.6–34.8 ppm. These shifts, particularly at donor-linked aromatic positions, provide compelling evidence of metal–ligand coordination. In complex 3, the spectrum displays characteristic downfield signals at δ 160.6, 156.7, and 137.1 ppm, which are attributed to the coordinated isoquinoline-thiazolidine framework. The arene carbons of the η^6^-coordinated *p*-cymene ring are observed between δ 85.30 and 95.5 ppm, consistent with other complexes. Aliphatic carbon resonances from the thiazolidine and isopropyl groups appear in the range of δ 31.2–52.0 ppm. These include methylene, methine, and methyl carbons, confirming the integrity of the ligand scaffold and its coordination environment. The deshielded aromatic carbons near the coordination sites indicate strong metal–ligand interaction. In complex 4, similar spectral features are observed. The aromatic carbon signals of the 5-methylpyridyl-thiazolidine moiety appear at δ 162.5, 156.2, and 141.5 ppm. The *p*-cymene carbons resonate between δ 85.1 and 96.3 ppm, in line with η^6^-arene coordination. Methyl and methylene carbons from the ligand and *p*-cymene side chain are present at δ 18.2–34.5 ppm. Notably, the appearance of multiple aliphatic carbon peaks confirms the presence of three methyl groups and a preserved thiazolidine ring. Overall, the observed shifts and signal patterns confirm successful coordination of the ligand to the Ru(ii) center in the complexes.

The presence of PF_6_^−^ as a counter ion is confirmed by ^19^F NMR (Fig. S21–S24), which shows a doublet signal at δ −70.13 ppm for all the complexes. This is because of the fact that the fluorine nuclei are directly coupled to the phosphorus nucleus (^31^P). This coupling splits the signal into a doublet. The P–F coupling constant (J_PF_) is approximately 756 Hz.

The ESI-LCMMS spectra (Fig. S9–S12 for the ligands and S25–S28 for the complexes) exhibit peaks at *m*/*z* 167.09, 181.19, 231.19 and 195 respectively for ligand 1 to 4 and [M–PF_6_]^+^ peaks at *m*/*z* 437.24 (complex 1), 451.54 (complex 2), 501.24 (complex 3), and 464.79 (complex 4), which are in excellent agreement with theoretical values. Additionally, FTIR spectra (Fig. S29–S32) reveal retained N–H stretching bands near 3350–3370 cm^−1^ and minor shifts in the C=N and C–N stretching regions, indicative of chelation *via* nitrogen atoms for all the complexes. Further, the peaks at 840 and 557 cm^−1^ demonstrate stretching and bending vibrations for PF_6_ ion respectively. Collectively, these spectroscopic data clearly confirm the formation of mononuclear Ru(ii) complexes bearing bidentate thiazolidine-based ligands.

### 4.2 Crystallographic structure description of complex 2

**Fig. 1:**
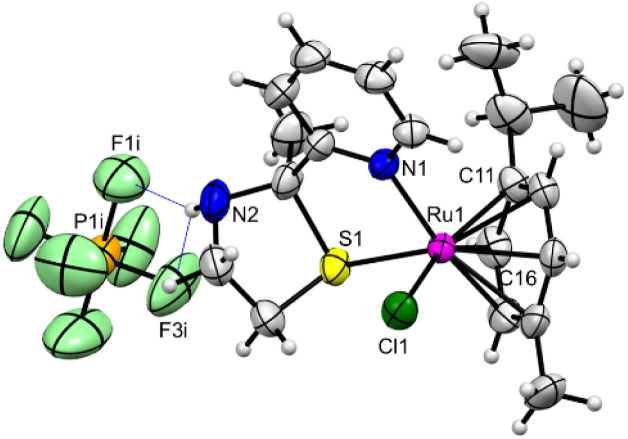
Molecular structure of complex 2 [Ruii(L2)(*p*-cymene)Cl](PF_6_) showing atom numbering scheme. Ellipsoids are drawn at the 50% probability level. Blue dashed lines denote N⋯H–F hydrogen bonds. Symmetry operation: *i* = 1.5 − *x*, 1 − *s*,0.5 + *z*.

A brown crystal of Ru complex 2 was analysed through single-crystal X-ray diffraction, revealing that it crystallizes in orthorhombic system with a space group *P*2_1_2_1_2_1_. This structure was refined as racemic twin with the BASF parameter = 0.45934. The complex contains one complex cation [Ruii(L2)(*p*-cymene)Cl]^+^ and one PF_6_^−^ anion as shown in Fig. 1. The Ru center is connected to the benzene ring of *p*-cymene ligand in an η_6_ fashion with distances ranging from 2.186 (3) to 2.236 (3) Å. The coordination sphere of the ruthenium is completed by one nitrogen atom N1 from the pyridine ring (Ru–N1 = 2.101 (2) Å), one sulfur atom (Ru–S1 = 2.3393 (7) Å) both from ligand L2 and one chloride anion (Ru–Cl1 = 2.186 (3) Å) (Table S2). All bond lengths around the Ru center are comparable with others Ru(ii) organo–metallic complexes described in CCDC Cambridge database.^34^

The PF_6_ anion establish hydrogen bonds of type N–H⋯F with the nitrogen atom N2 from the sulfur cyclopentane ring with dimensions of 3.346 (5) and 3.240 (4) Å. No classical hydrogens bonds are observed also of type C–H⋯F and C–H⋯Cl (Table S3).

### 4.3 Stability check in solution and lipophilic characteristics of the complexes

The stability of the complexes was assessed over three consecutive days using UV-vis spectroscopy. Measurements were taken on days 1, 2, 3 and the results showed that there was virtually no change in the type of spectral properties, including the spectrum's nature, intensity, and the solution's colour. This suggests that all the complexes remain stable in the working buffer solution even after three days (Fig. S33a–d).

Additionally, a lipophilicity test was conducted by calculating the log *P*_o/w_ values, which was found to be −0.93, −0.77, −0.96 and −1.2 for complex 1 to 4 respectively. These values indicate that all the complexes have good lipophilic character, as the values fall within the typical range of cisplatin (−2.28 ± 0.07) reported in the literature demonstrating that the complexes mostly remain in water part having potentiality to be a drug.^35,36^

### 4.4 Antioxidant activity of the complexes

The study aimed to evaluate the free radical scavenging or antioxidant activities of the complexes using the *in vitro* DPPH assay method. This assay is a well-established method for measuring antioxidant properties and involves determining the IC_50_ values, which represent the concentration required to inhibit 50% of the free radicals present.^37–39^ The results are visually represented in Fig. S34a to d, with a numerical analysis provided in Table 1.

**Table 1:** IC_50_ values for all the complexes obtained from DPPH assay

Sample	IC_50_ value (μg mL^−1^)
Complex 1	7.8
Complex 2	8.2
Complex 3	7.4
Complex 4	7.5

The IC_50_ values obtained reveal that the complexes possess significant free radical scavenging capabilities, making it a promising candidate for antioxidant applications. When compared to ascorbic acid, a widely recognized standard antioxidant and the IC_50_ value for which typically falls in the range of 24.34 ± 0.09 μg mL^−1^ with some studies reporting values as low as 8.4 μg mL^−1^, our complexes demonstrated very much comparable efficacy in neutralizing free radicals (Table 1).

The change in colour of the DPPH solution serves as a qualitative confirmation of the complex's antioxidant activity. Pure DPPH solutions exhibit a deep violet colour, and the addition of antioxidants typically results in a noticeable colour shift towards yellow as the free radicals are neutralized. In this study, the introduction of the complex led to a significant colour change, as shown in Fig. S35a to d which further supports the quantitative IC_50_ data. This visual evidence underscores the complexes' ability to effectively scavenge free radicals.

Given these promising results, we were motivated to extend our research to explore the interaction behaviour of the complex with biomolecules, particularly DNA and BSA. Understanding these interactions is crucial, as it can provide insights into the complex's potential therapeutic applications, including its role in preventing oxidative damage to cellular components and its binding affinity with essential biomolecules. Such interactions can also shed light on the complex's mechanism of action in biological systems, paving the way for future investigations into its potential as a therapeutic agent. So, this study not only highlights the complex's potential as a free radical scavenger but also lays the groundwork for further research into its interactions with biologically relevant molecules.

### 4.5 Insights on binding interactions of the complexes with CT-DNA and BSA

#### 4.5.1 DNA binding studies for all the complexes

**Fig. 2:**
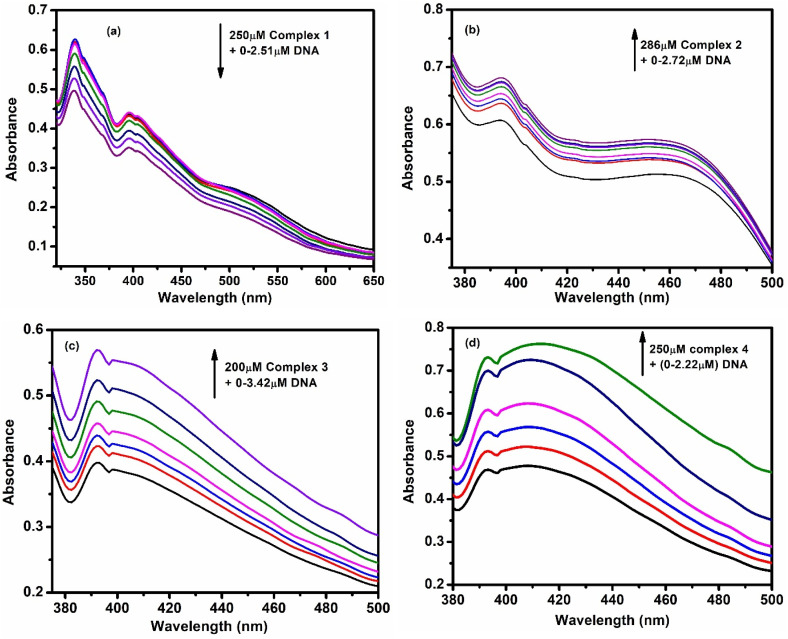
UV spectral changes with increasing concentration of DNA (a) for complex 1 (b) for complex 2 (c) for complex 3 and (d) for complex 4.

UV-vis absorption spectroscopy was used to study how the complexes interact with CT-DNA. For each complex, their absorbance was recorded while increasing the DNA concentration. In Fig. 2a–d, it is shown that absorbance decreased for complex 1 and increased for the other three as DNA was added. These changes suggest strong interactions between the complexes and DNA. The binding strength was measured using the Wolfe–Shimmer eqn (3), and the binding constants (*K*_ib_) were found to be 6.73 × 10^5^, 4.97 × 10^5^ 2.93 × 10^5^ and 3.55 × 10^5^ respectively for complex 1, 2, 3 and 4 (Fig. S36a–d).

3 [DNA] × (*ε*_a_ − *ε*_f_)^−1^ = [DNA] × (*ε*_b_ − *ε*_f_)^−1^ + *K*_b_^−1^× (*ε*_b_ − *ε*_f_)^−1^

where *ε*_a_, *ε*_f_ and *ε*_b_ are the extinction coefficients of the complex, CT-DNA and bound complex, respectively.

To understand the mode of binding, fluorescence experiments were done, including ethidium bromide (EB) displacement and helix melting studies.

**Fig. 3:**
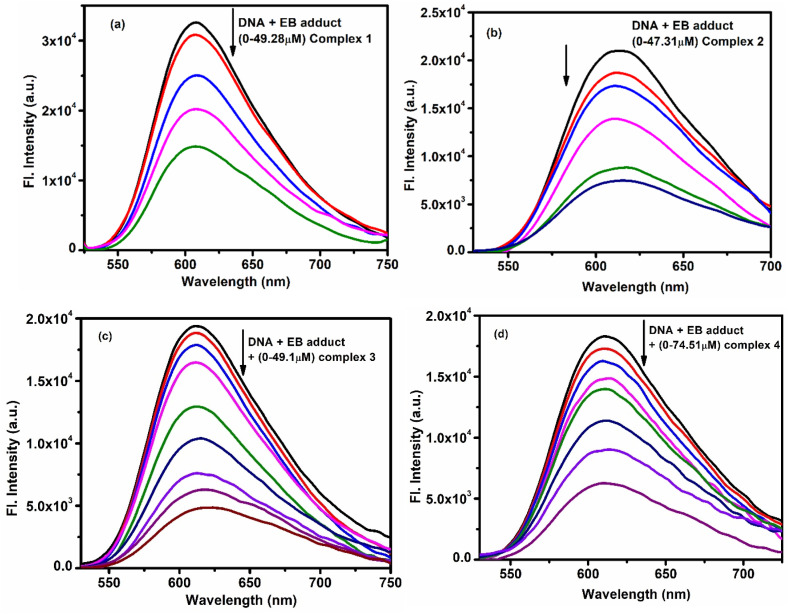
Emission intensity quenching of DNA –EB adduct with gradual addition of (a) complex 1 (b) complex 2 (c) complex 3 and (d) complex 4.

For the EB displacement experiment, CT-DNA was mixed with EB and excited at 612 nm. As the complexes were added, the fluorescence of EB-DNA decreased (Fig. 3a–d), showing that the complexes were replacing EB on the DNA. This quenching indicates strong binding. The Stern–Volmer eqn (4) was used to calculate the quenching constant (*K*_sv_).

4 *F*_0_/*F* = *K*_sv_ [Q] + 1

In this context, *F*_0_ and *F* represent the fluorescence intensities in the absence and presence of the complex, respectively, while *K*_sv_ denotes the Stern–Volmer constant, and [Q] is the concentration of the complex.

The following Scatchard eqn (5) helped to determine the binding constant (*K*_b_) and the number of binding sites (*n*).

5 log[(*F*_0_ − *F*)/*F*] = log *K*_b_ + *n* log[complex]

The plot of log[(*F*_0_ − *F*)/*F*] against log[complex] produces a straight line, where the slope corresponds to *n* and the intercept represents log *K*_b_. The observed results (shown in Fig. S37a–d, S38a–d, and Table 2) confirm strong binding of all complexes with DNA.

**Table 2:** Binding parameters for displacement studies of the complexes with DNA-EB and DNA-hoechst binding system

Binding system	Sample	*K*_sv_	*K*_b_	*n*
DNA-EB	Complex 1	3.22 × 10^4^	4.46 × 10^4^	2.19
	Complex 2	4.00 × 10^4^	2.24 × 10^4^	1.56
	Complex 3	5.07 × 10^4^	4.78 × 10^3^	2.01
	Complex 4	2.19 × 10^4^	9.12 × 10^3^	1.20
DNA-hoechst	Complex 1	1.26 × 10^4^	4.78 × 10^4^	1.02
	Complex 2	1.24 × 10^4^	9.33 × 10^4^	1.27
	Complex 3	7.56 × 10^4^	2.09 × 10^5^	0.78
	Complex 4	9.32 × 10^4^	2.39 × 10^5^	0.72

**Fig. 4:**
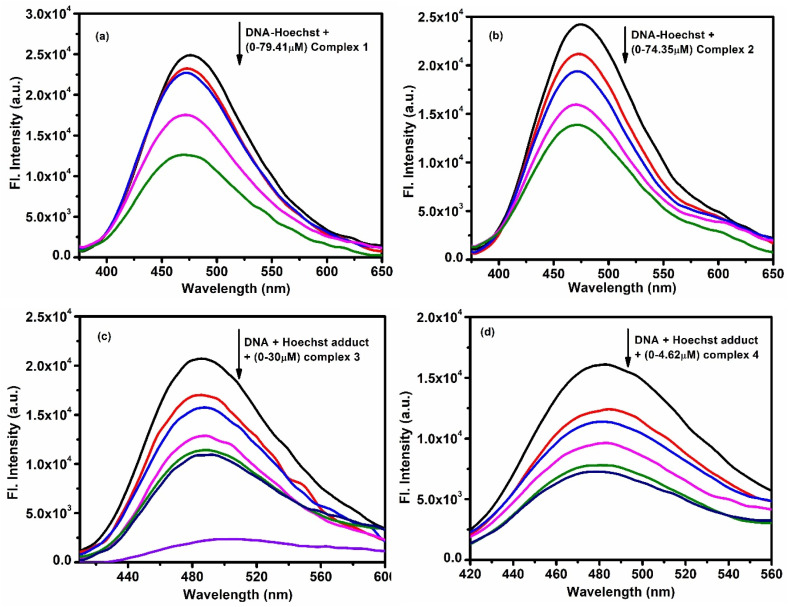
Emission intensity quenching of DNA – Hoechst adduct with gradual addition of (a) complex 1 (b) complex 2 (c) complex 3 and (d) complex 4.

Another test was done using Hoechst dye, a known groove binder. Changes in the fluorescence emission at 475 nm were monitored as the complexes were added (Fig. 4a–d). Binding parameters were again calculated (Fig. S39a–d and S40a–d), and the *K*_b_ values were slightly higher than in EB studies, suggesting a partial groove binding mode for all the complexes.^40,41^

**Fig. 5:**
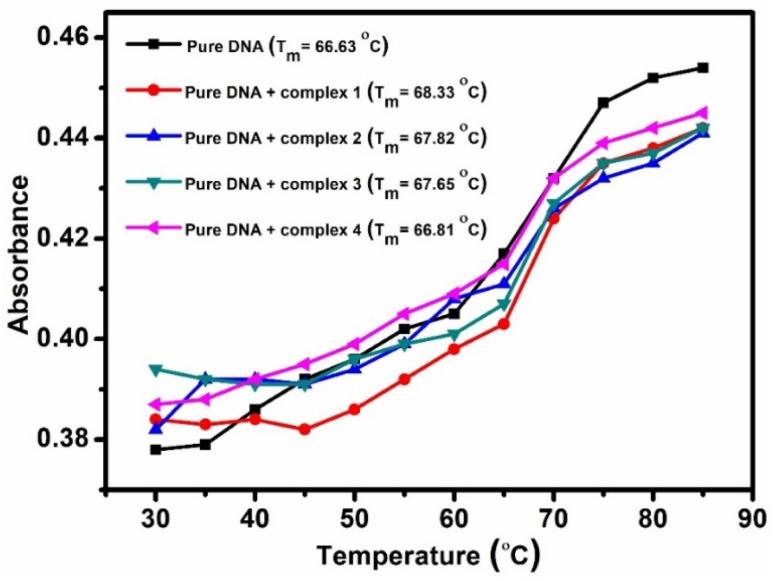
Melting temperature of pure DNA and DNA combined with the complexes.

To further confirm the mode of binding, helix melting experiments were performed. When DNA binds through intercalation, its melting temperature (*T*_m_) increases significantly by more than 5 °C. However, in our study (Fig. 5), *T*_m_ increased only by about 1.70 °C, 1.22 °C, 1.02 °C and 0.18 °C for complex 1, 2, 3 and 4 respectively. This small increase (less than 5 °C) in *T*_m_ suggests that all the complexes bind to DNA through the groove mode of binding.^42–44^

#### 4.5.2 BSA binding interaction studies by absorption and fluorescence spectroscopy

**Fig. 6:**
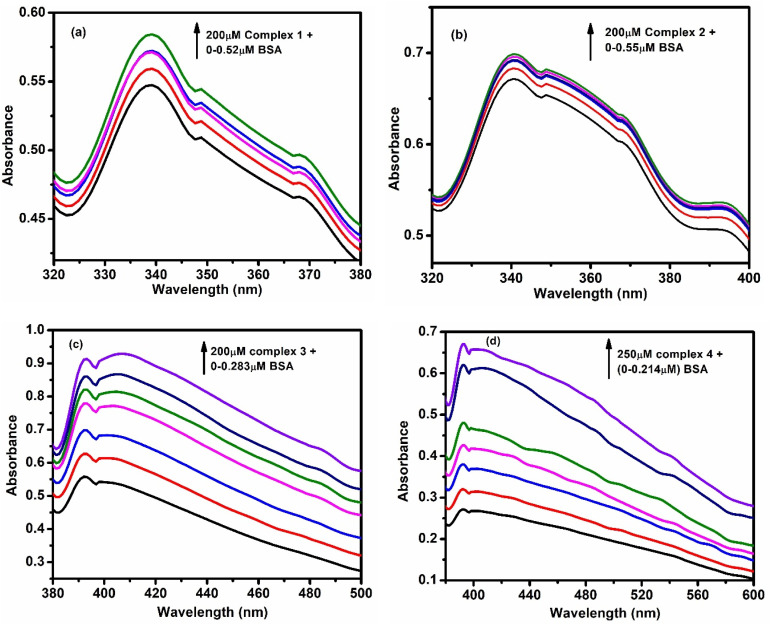
UV spectral changes of (a) complex 1 (b) complex 2 (c) complex 3 and (d) complex 4 with increasing concentration of BSA.

The interaction between the complexes and BSA was studied using UV-vis and fluorescence spectroscopy. UV results showed that with increasing BSA concentration, the absorbance of the complexes increased (Fig. 6a–d), indicating strong interaction. The apparent binding constants (*K*_app_) were calculated as 5.99 × 10^5^, 2.54 × 10^5^, 1.29 × 10^5^ and 4.94 × 10^5^, respectively (Fig. S41a–d).

**Fig. 7:**
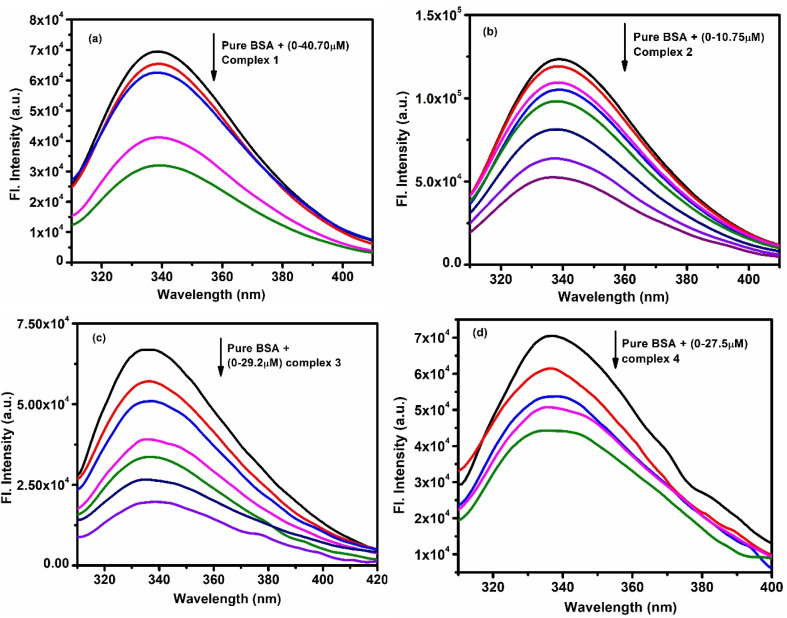
Emission intensity quenching of BSA with gradual addition of (a) complex 1 (b) complex 2 (c) complex 3 (d) complex 4.

Fluorescence studies showed a gradual decrease in BSA's emission intensity at ∼335 nm as more complex was added (Fig. 7a–d), confirming fluorescence quenching. Using eqn (4)–(6), the quenching constants (*K*_sv_ and *K*_q_), binding constants (*K*_b_), and the number of binding sites (*n*) were calculated. Here, *τ*_0_ is the fluorescence lifetime of BSA without any quencher (about 5 × 10^−9^ s).

6 *K*_sv_ = *K*_q_*τ*_0_

The data (Table 3) and supporting plots (Fig. S42a–d and S43a–d) suggest that the complexes interact with BSA mainly through a single, static quenching mechanism.

**Table 3:** Binding parameters of complexes for BSA interaction

Sample	*K*_sv_	*K*_b_	*K*_q_	*n*
Complex 1	3.2 × 10^4^	3.8 × 10^4^	6.4 × 10^12^	1.88
Complex 2	1.2 × 10^5^	1.9 × 10^4^	2.4 × 10^13^	0.77
Complex 3	4.9 × 10^4^	1.2 × 10^5^	9.8 × 10^12^	1.02
Complex 4	5.2 × 10^4^	1.3 × 10^5^	1.1 × 10^13^	1.14

### 4.6 Antibacterial effects of the complexes

The susceptibility of Gram-positive and Gram-negative bacteria to our complexes under present investigation was explored *via* a cup plate assay using MRSA and KP, respectively, as test organisms. Following overnight incubation at the designated temperature, the diameter of the clear area surrounding each well was measured; this area referred to as the “zone of inhibition” depicts the extent to which bacterial growth is suppressed by the examined complexes. Cefoxitin was employed as the standard antibiotic control in the cup plate assay to evaluate the antimicrobial efficacy of the synthesized Ru complexes against MRSA and KP. As because of the fact that cefoxitin is a second-generation cephamycin with broad-spectrum activity and is recommended by the Clinical and Laboratory Standards Institute (CLSI) as a substitute marker for detecting methicillin resistance in *S. aureus* due to its strong induction of the *mecA* gene. Additionally, cefoxitin maintains activity against certain β-lactamase-producing Gram-negative organisms, making it a suitable comparator for assessing efficacy against KP. Its well-characterized diffusion profile in agar-based methods also ensures reproducible and consistent zone formation, making it an appropriate and reliable standard for benchmarking novel antimicrobial agents such as Ru complexes.

The experimental results reveal that complexes 1 and 2 demonstrated antibacterial activity through the formation of measurable inhibition zones. Complexes 3 and 4 failed to display inhibitory activity against MRSA and KP so they were eliminated from further study. In our previous study (Das *et al.* 2024),^32^ we have already seen that the MRSA700699 strain showed resistance towards B lactam antibiotic cefoxitin according to the CLSI Guideline. However, the Ni(ii) Schiff base complex^32^ produced a significant inhibition zone against MRSA yet failed to show activity against KP. In this study, our Ru(ii) complexes 1 and 2 displayed substantial inhibitory activity against both MRSA and KP even though KP remains challenging to treat due to its complex outer membrane structure.

**Fig. 8:**
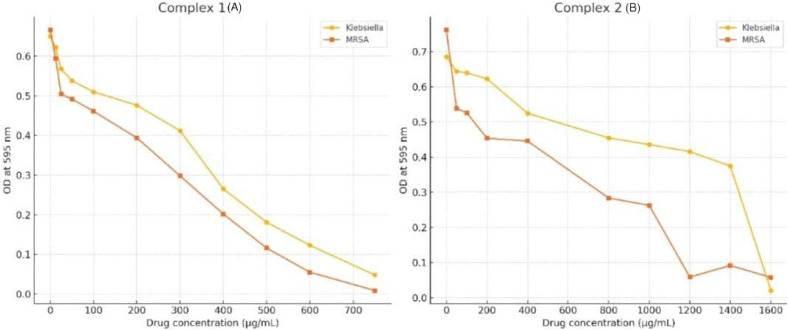
Determination of MIC (A) The MIC value of complex 1 against KP and MRSA at 500 μg mL; (B) the MIC value of complex 2 against KP and MRSA at 1600 μg mL^−1^ and 1200 μg mL^−1^, respectively; the data provided represent the mean ± standard deviation (0.05) of triplicate experiments.

The MIC values were determined through broth microdilution tests for both bacterial strains. Bacterial growth was evaluated by measuring the post-incubation (24 h at 37 °C) optical density (OD) at 595 nm. The results depicted that both the complexes reduced bacterial growth of MRSA and KP in a dose-dependent manner. Complex 1 displayed MIC values of 500 μg mL^−1^ for MRSA and KP whereas, complex 2 displayed MIC values of 1200 μg mL^−1^ for MRSA and 1600 μg mL^−1^ for KP as illustrated graphically in Fig. 8. The MBC of complex 1 was 500 μg mL^−1^ against KP which shows its bactericidal properties. Complex 2 demonstrated bactericidal effects against KP but required higher concentrations reaching an MBC of 2000 μg mL^−1^. And in case of MRSA, 400 μg mL^−1^ and 1600 μg mL^−1^ were the MBC for complex 1 and complex 2 respectively (Fig. S44). The aforementioned findings imply that our complexes demonstrated MIC and MBC values that surpassed those of the Ni(ii) Schiff base complex reported by Das *et al.* 2024.^32^ However complex 1 demonstrated bactericidal activity against both MRSA and KP compared to Ni(ii) complex which failed to inhibit KP. Therefore, this Ru(ii) complex 1 and complex 2 exhibits promising broad-spectrum antibacterial activity especially when addressing Gram-negative resistance.

The comparison between complex 1 and complex 2 shows that complex 1 is almost equally potent against KP and MRSA whereas, complex 2 is more effective against MRSA as compared to KP. The differential responses against Gram-positive and Gram-negative bacteria induced by complex 2 may be attributed to distinctions in cell wall structure and permeability.^45^ The potential activity of complexes 1 and 2 and inability of the complexes 3 and 4 to inhibit both the MRSA and KP growth may be attributed indirectly with Highest Occupied Molecular Orbital (HOMO) – Lowest Unoccupied Molecular Orbital (LUMO) gap predictor of antibacterial activity. Studies have shown that metal complexes with large HOMO–LUMO gaps (*e.g.* 2–3 eV) may lack significant antibacterial activity. Conversely, some metal complexes with smaller HOMO–LUMO gaps (*e.g.* 1–2 eV) have demonstrated antibacterial activity against specific bacterial strains. In general, a larger HOMO–LUMO gaps often suggests a more stable molecule which might not have the reactivity needed for antibacterial action. In our present study, the experimentally obtained HOMO–LUMO gap values are found as 1.51 and 1.63 eV for complexes 1 and 2 whereas for complexes 3 and 4 these are 2.34 and 2.14 eV respectively (calculated using the Tauc formula). This may be one of the key electronic structure–activity relationships that help to explain why some Ru complexes exhibit antibacterial activity while others do not. The superior antibacterial efficacy of complex 1 than complex 2 may be due to its lower HOMO–LUMO gap value than complex 2 to some extent. Other factors such as ligand variation in complex 2 may also play a crucial role.

### 4.7 Hemolysis biocompatibility assay

**Fig. 9:**
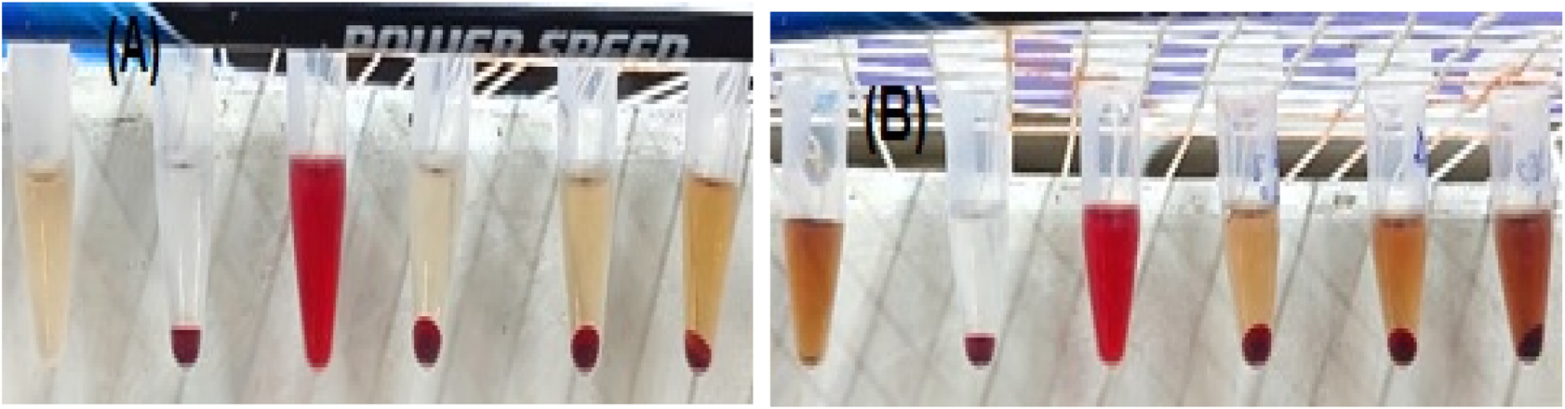
Hemolysis biocompatibility assay (A) complex 1 and (B) complex 2. From left to right, 1% DMSO-solubilized complexes, PBS-treated cells (negative control), Triton X-100-treated cells (positive control), cells treated with complexes in increasing concentrations. Neither complex 1 (250, 500, and 1000 μg mL^−1^) nor complex 2 (800, 1600, and 3200 μg mL^−1^) demonstrated hemolysis of erythrocytes. The 1% DMSO-solubilized tubes have been employed as reference for calibration of coloured complexes.

A hemolysis assay was performed to evaluate the hemocompatibility of complex 1 and complex 2. Red blood cells (RBCs) in heparin-stabilized blood were incubated with varying concentrations of complex 1 (250, 500, and 1000 μg mL^−1^) and complex 2 (800, 1600, and 3200 μg mL^−1^) for 4 h. The complex 1- and complex 2-treated groups exhibited negligible hemolysis, comparable to that of the negative control group, whereas the positive control treated with Triton X-100 demonstrated lysis of RBCs, absence of visible RBC precipitation, and a uniform red coloration post-centrifugation (Fig. 9). The results indicated that the complexes evidence minimal cytotoxicity and enhanced biocompatibility and, consequently, endorse their potential for *in vivo* administration.^46,47^

### 4.8 SEM analysis

**Fig. 10:**
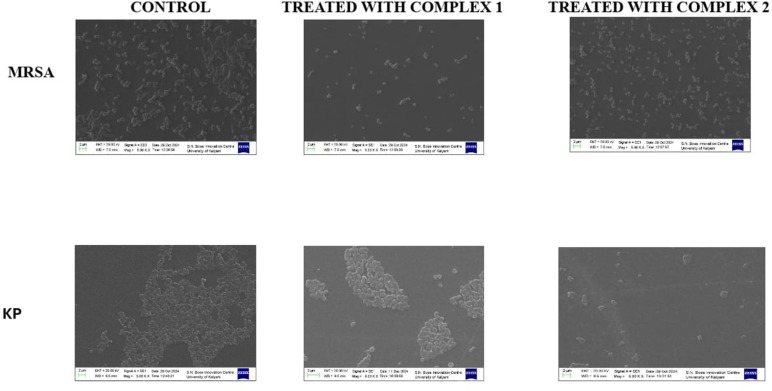
SEM images showing the effect of complex 1 and complex 2 on MRSA and KP. Treated with half-MIC doses of complex 1 and complex 2 resulted in a significant reduction in the number of bacterial cells and also affect shape of bacterial cells. In case of KP, individual cells became dispersed and lost their aggregated arrangement, indicating disruption of biofilm or cell-to-cell interactions. For MRSA, the production of EPS was significantly reduced, resulting in the disruption of coccal chains. These morphological changes were observed under SEM.

The effects of complex 1 and complex 2 on MRSA and KP were evaluated at half-MIC concentrations. Both complexes demonstrated significant antibacterial activity, as evident by a reduction in cell numbers for both organisms (Fig. 10). In the case of KP, cells were observed to be isolated from one another after treatment, suggesting disruption of cell-to-cell adhesion and possible inhibition of biofilm formation. Similarly, MRSA cells exhibited reduced extracellular polymeric substances (EPS) production, leading to the absence of typical coccal chains, which are characteristic of untreated MRSA. These findings indicated that complex 1 and complex 2 may exert their antibacterial effects not only through direct inhibition of bacterial growth but also by interfering with key structural and adhesive properties, such as EPS production and cellular aggregation. Further studies are required to elucidate the precise mechanisms of action.

## 5. Conclusion

This study presents the synthesis and detailed characterization of four thiazolidine based Ru(ii) complexes, highlighting their structural and biological properties. The findings underscore the potential antibacterial activity of complexes 1 and 2 against KP and MRSA among the four synthesized complexes. The DNA and BSA interaction studies demonstrating a groove mode of binding for all the complexes with DNA and a single, static quenching interaction between the complexes and BSA. The results also pointed out that the complexes 1 and 2 ascertain minimal cytotoxicity and enhanced biocompatibility and, consequently, endorse their potential for *in vivo* administration as antibacterial therapeutic agents. The observed antibacterial performance particularly, the potent activity of selected complexes (1 and 2) against resistant strain, demonstrates the potential of Ru(ii)-thiazolidine frameworks as next-generation antimicrobial agents, offering a promising avenue for addressing antimicrobial resistance in clinical contexts.

## Author contributions

Debasis Bhunya: investigation, methodology; Riya Datta: supervision; Ribhu Maity: investigation, methodology; Alipe Saha: investigation, methodology; Sujata Sen: investigation, data curation; Paula Brandao: software, validation; Satyajit Pattanayak: investigation, methodology; Tithi Maity: data curation, formal analysis; Keka Sarkar: writing – review and editing, supervision; Bidhan Chandra Samanta: conceptualization, writing – original draft and supervision.

## Conflicts of interest

No conflicts of interest to declare.

## Supplementary Material

RA-015-D5RA05336F-s001

RA-015-D5RA05336F-s002

## Data Availability

The data supporting this study's findings are available from the corresponding author, upon reasonable request. CCDC 2376098 contain the supplementary crystallographic data for this paper.^48^ Supplementary information is available. See DOI: https://doi.org/10.1039/d5ra05336f.
