# Supplementary material for: Design and synthesis of bioactive Ru(ii) complexes: antibacterial activity, biocompatibility and biomolecular binding
Source: RSC Adv. 2025 Nov 4;15(50):42573–87. doi: 10.1039/d5ra05336f (PMC12584959; doi:10.1039/d5ra05336f)
Supplement: RA-015-D5RA05336F-s001 [file RA-015-D5RA05336F-s001.pdf]

Supplementary Information (SI) for RSC Advances.
This journal is © The Royal Society of Chemistry 2025

Supporting Information

Design and Synthesis of Bioactive Ru(II) Complexes: Antibacterial Activity, Biocompatibility and Biomolecular Binding

Debasis Bhunya[a], Riya Datta*[a], Ribhu Maity[b], Alipe Saha[c], Sujata Sen[c], Paula Brandao[d], Satyajit Pattanayak[b], Tithi Maity[e], Keka Sarkar*[c] and Bidhan Chandra Samanta*[b]

[a]*Department of Chemistry, Christ University, Hosur Road, Bengaluru-560029, Karnataka, India.*

[b]*Department of Chemistry, Mugberia Gangadhar Mahavidyalaya, Bhupatinagar, Purba Medinipur-721425, West Bengal, India.*

[c]*Department of Microbiology, University of Kalyani, West Bengal, India*

[d]*Departamento de Química, CICECO, Universidade de Aveiro, 3810-193 Aveiro, Portugal*

[e]*Department of Chemistry, Prabhat Kumar College, Purba Medinipur-721401, Contai, West Bengal, India*

***Corresponding Email:** riya.datta@christuniversity.in **(RD);** bidhansamanta@yahoo.in **(BCS)**

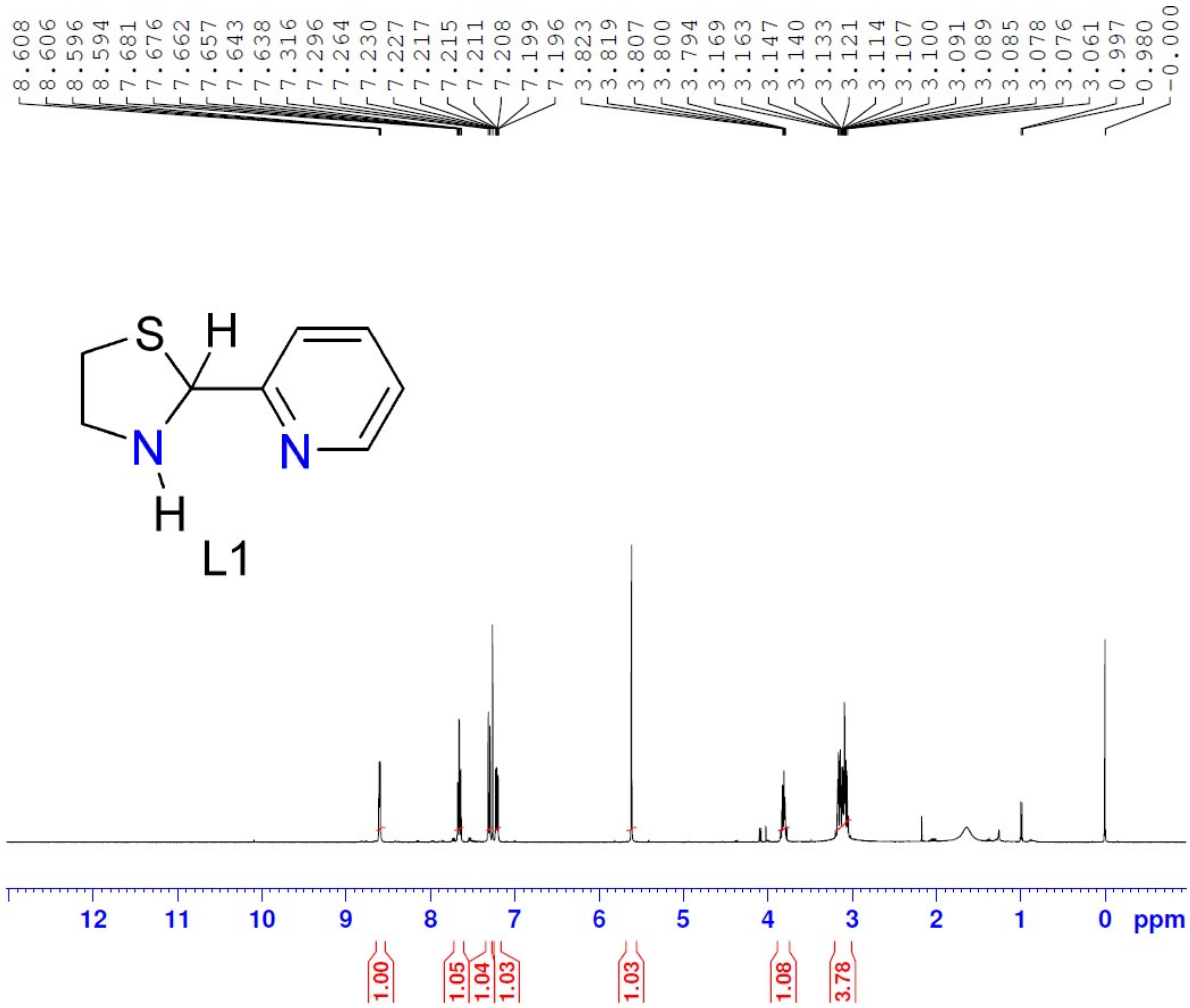

Fig. S1 ^{1}H- NMR spectrum of ligand L1

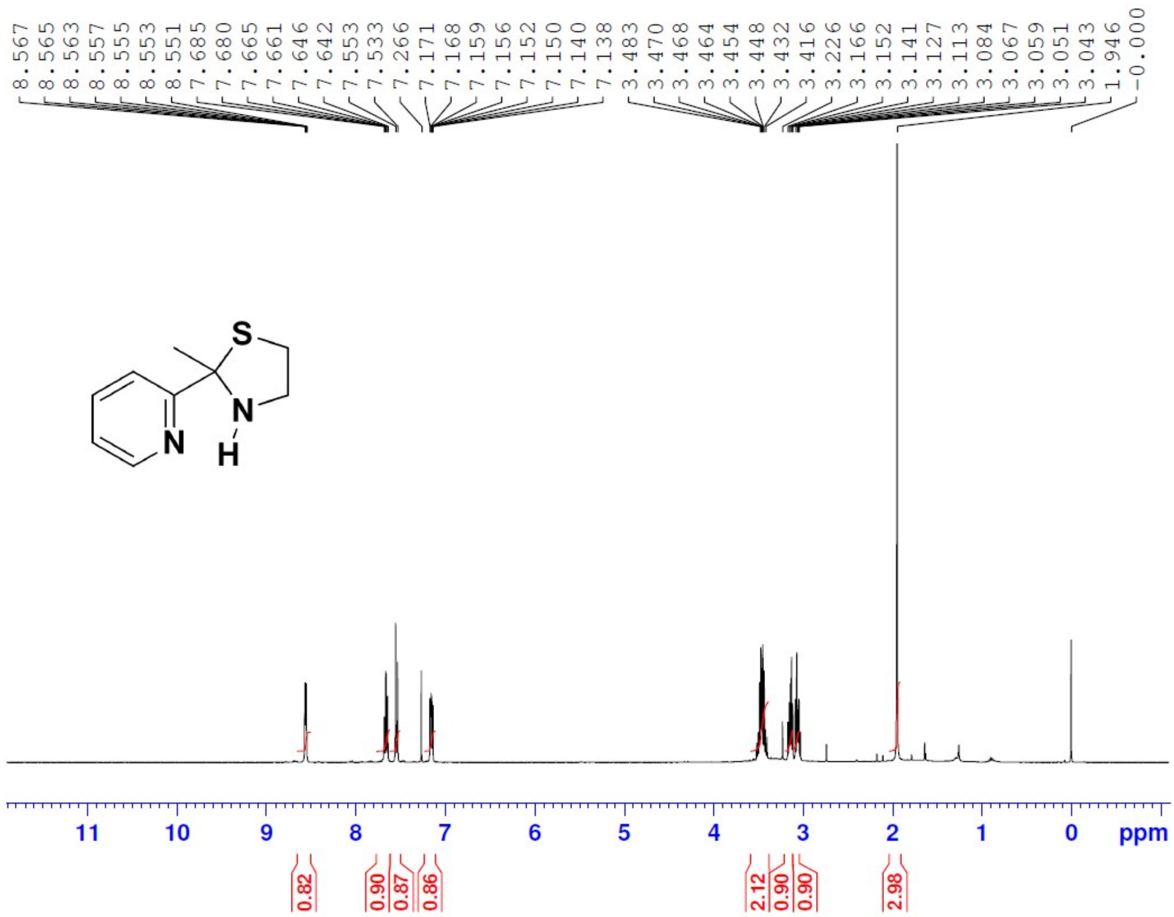

Fig. S2 ^{1}H- NMR spectrum of ligand L2

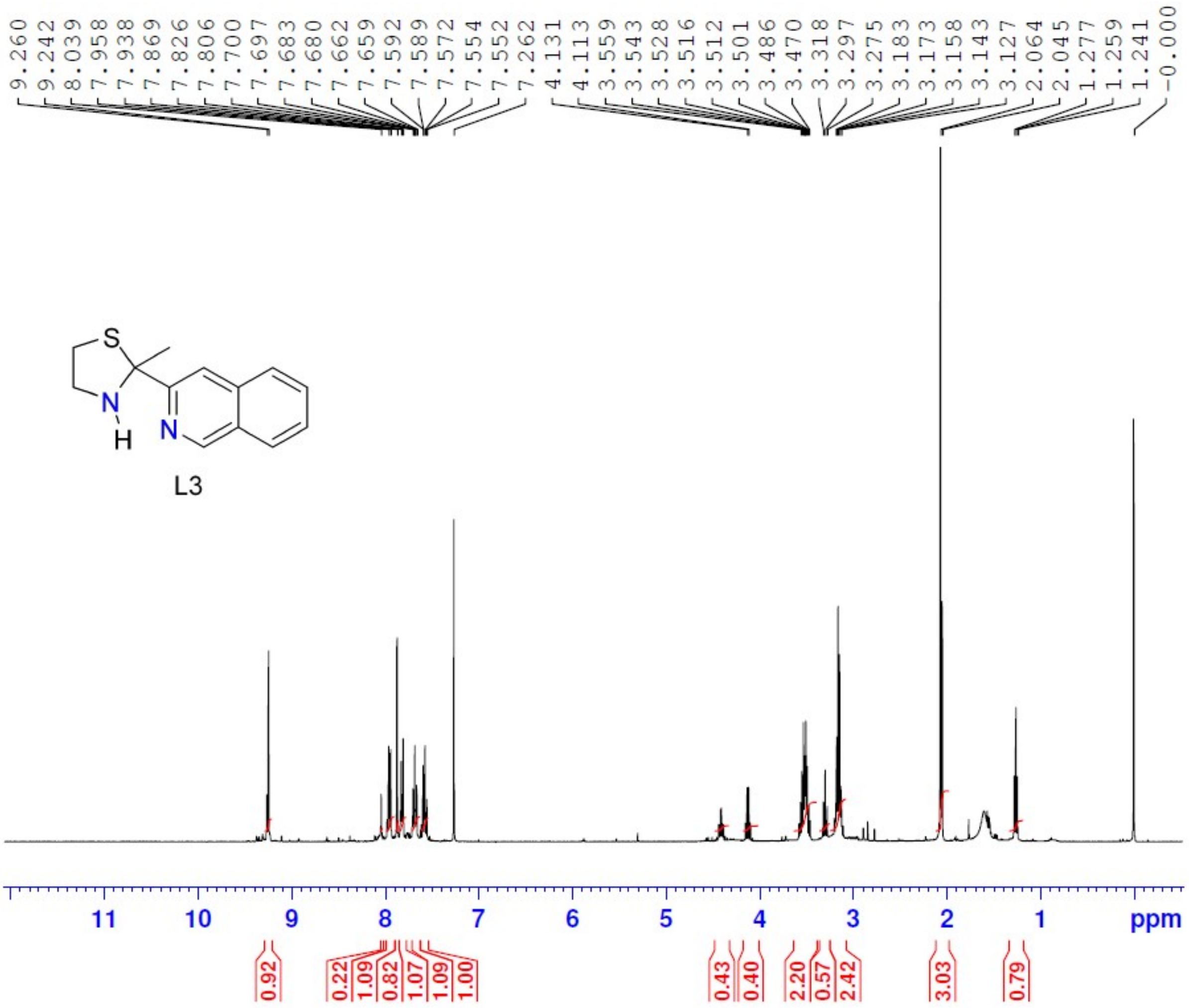

Fig. S3 ^{1}H- NMR spectrum of ligand L3

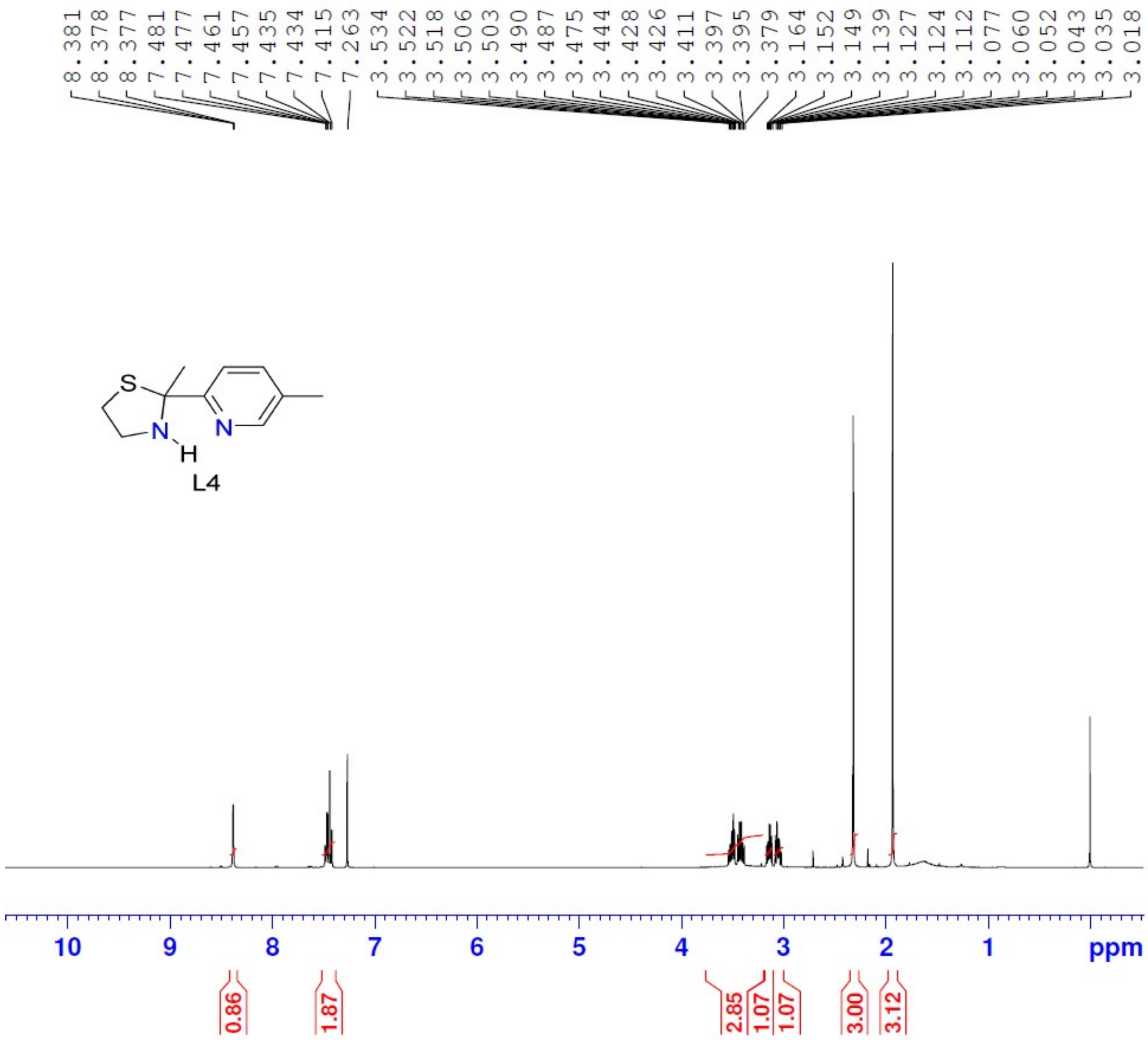

Fig. S4 ^{1}H- NMR spectrum of ligand L4

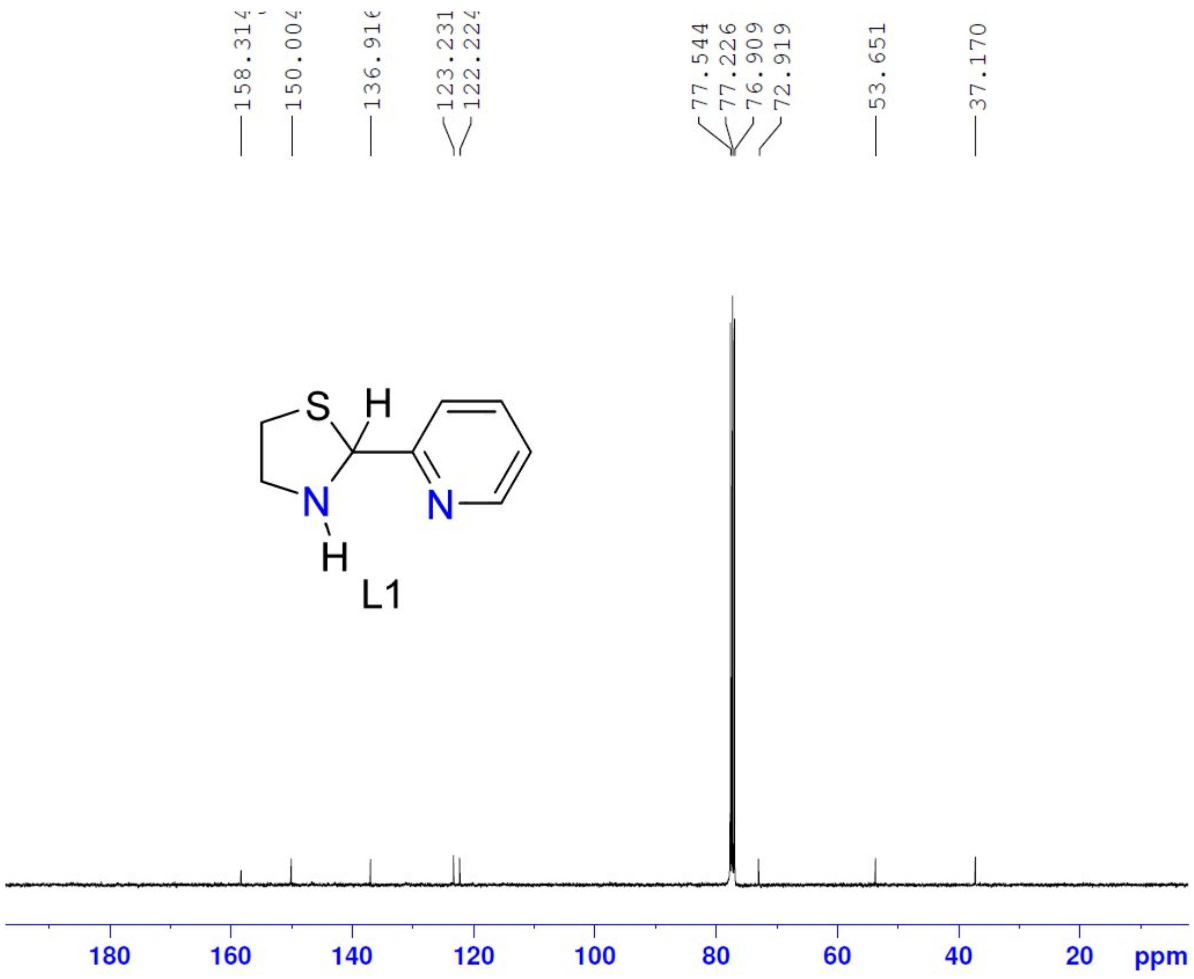

Fig. S5 ^{13}C- NMR spectrum of ligand L1

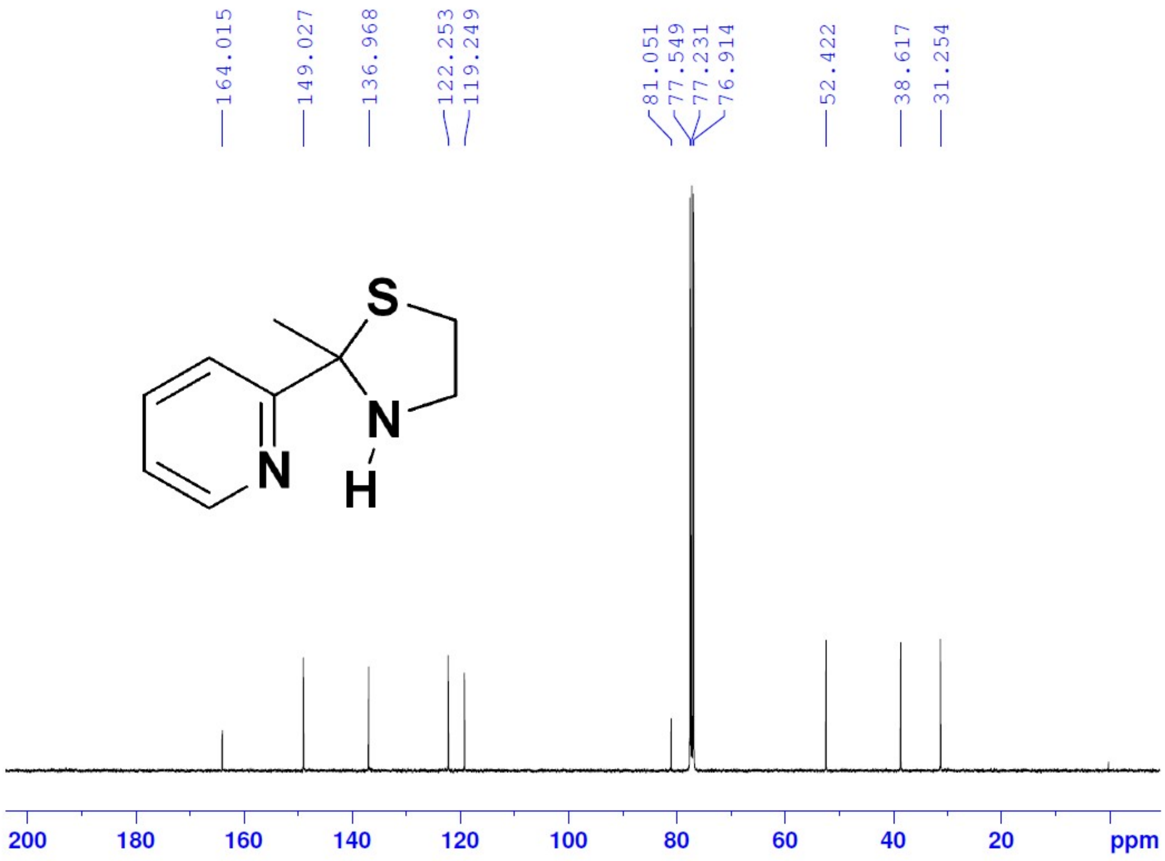

Fig. S6 ^{13}C- NMR spectrum of ligand L2

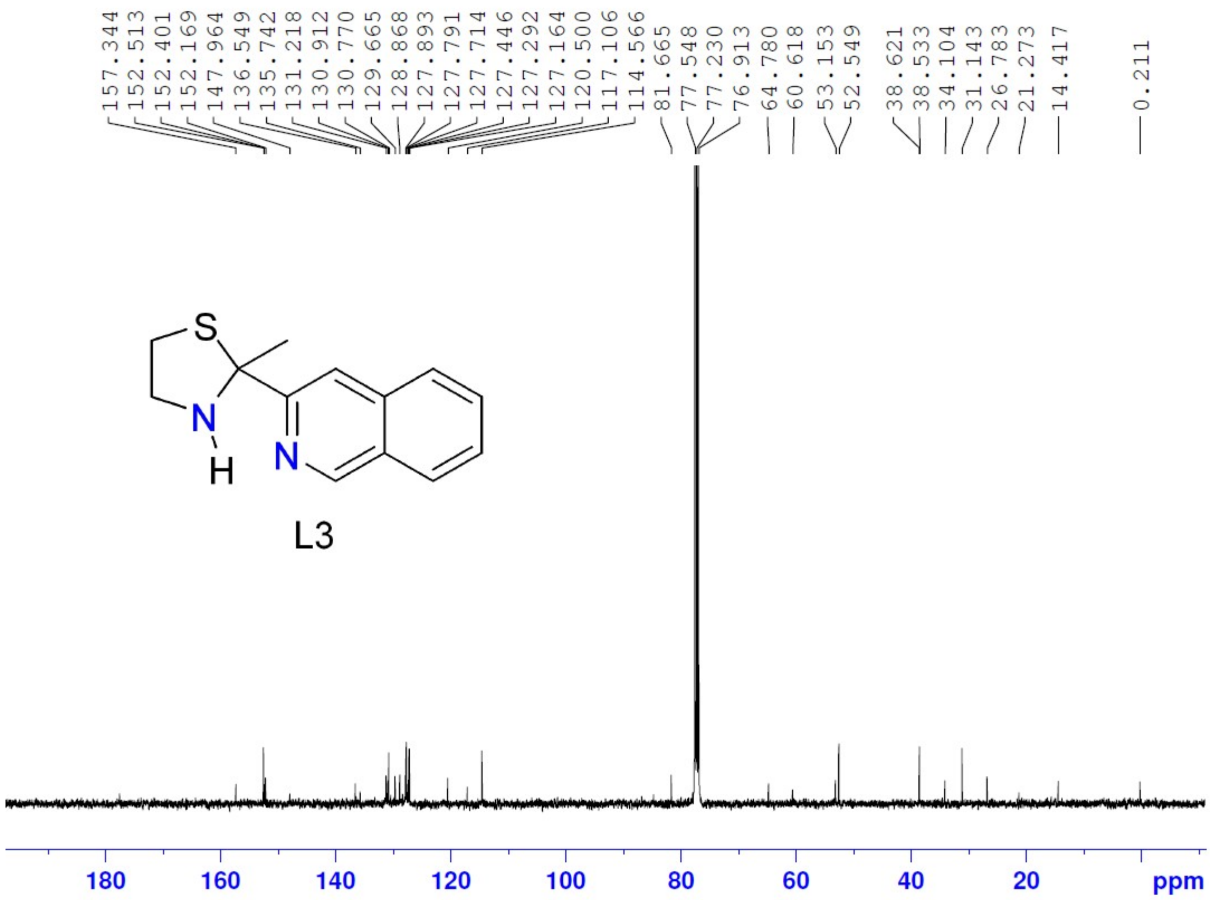

Fig. S7 ^{13}C- NMR spectrum of ligand L3

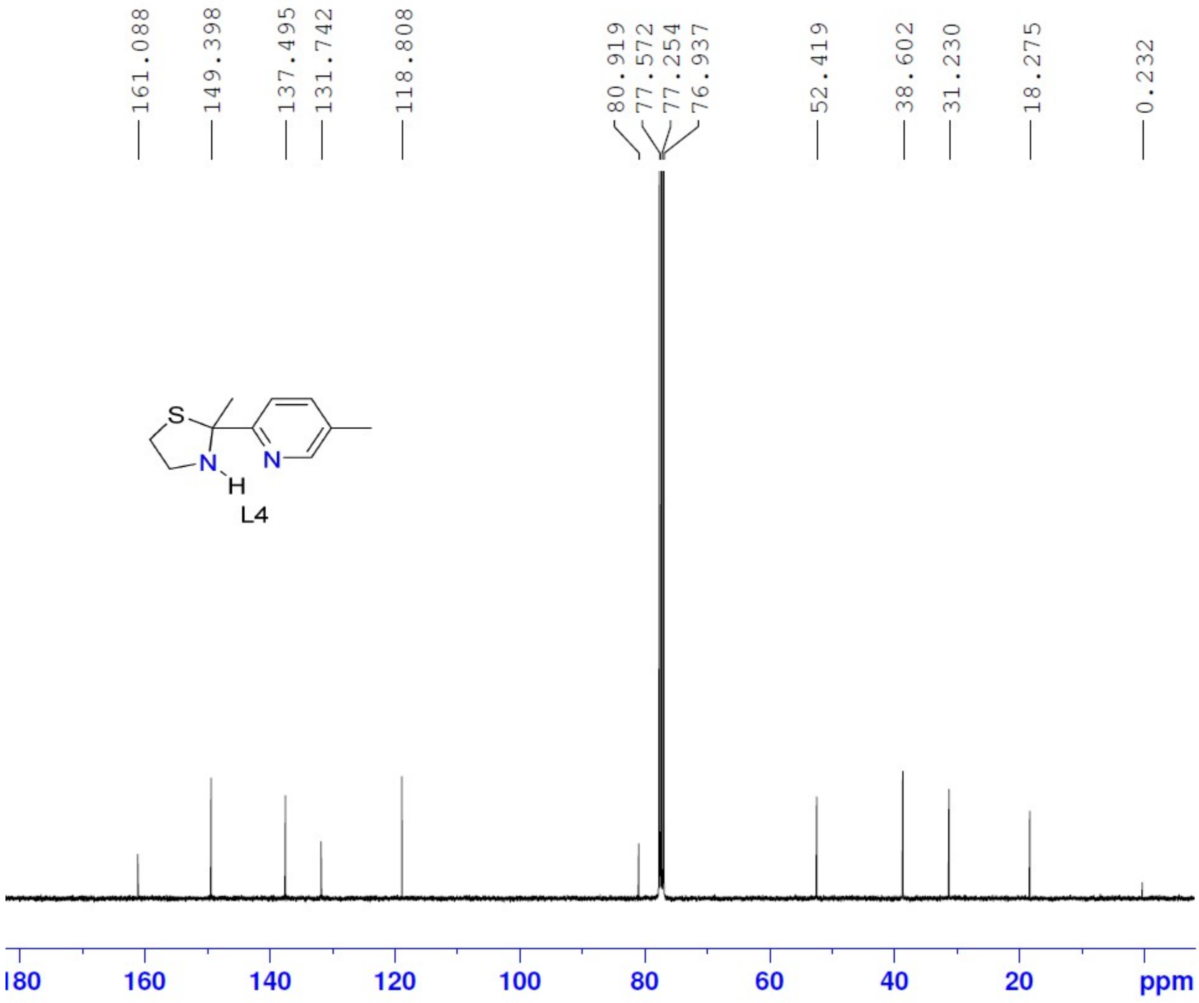

Fig. S8 ^{13}C- NMR spectrum of ligand L4

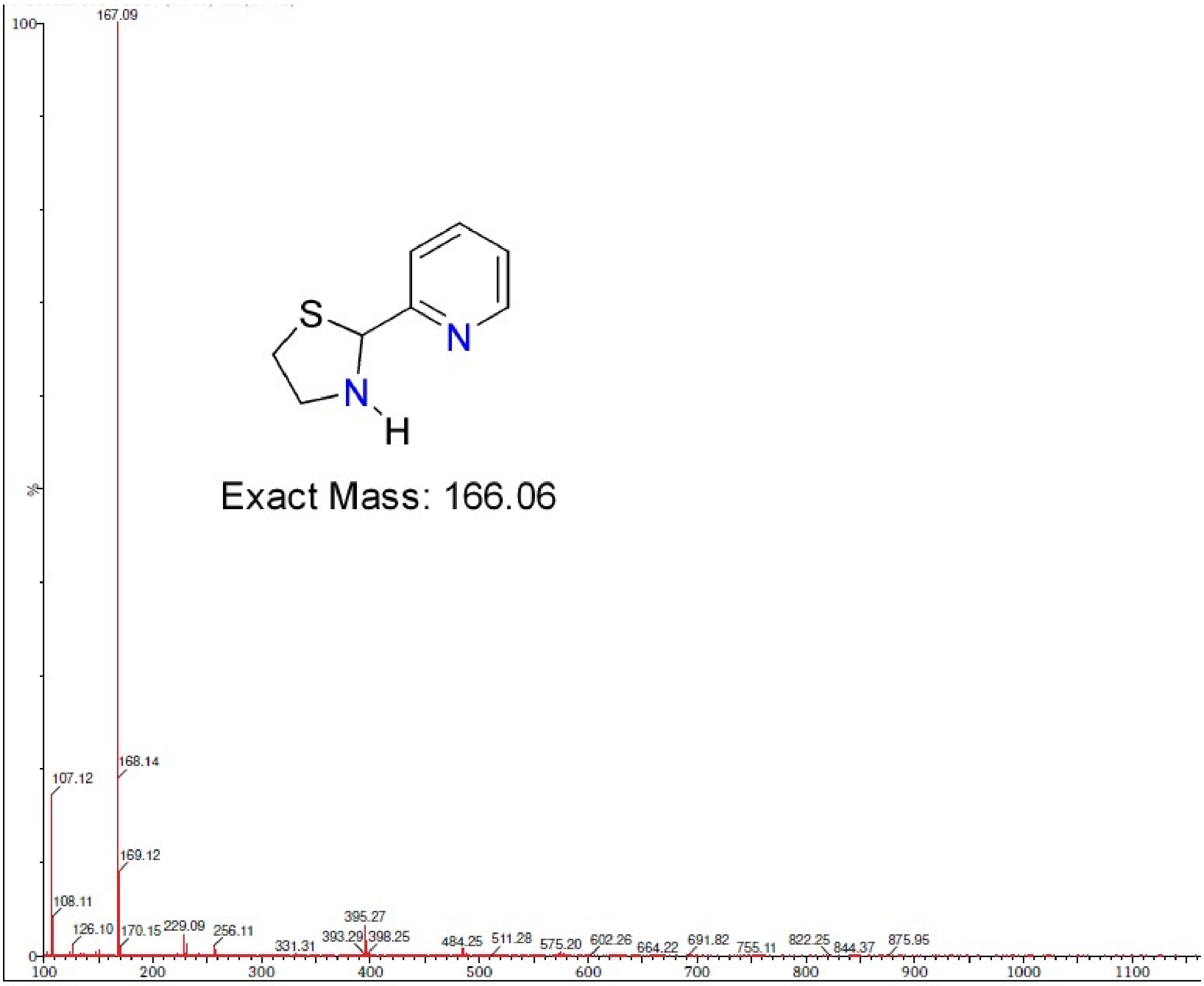

Fig. S9 Mass spectrum of ligand L1

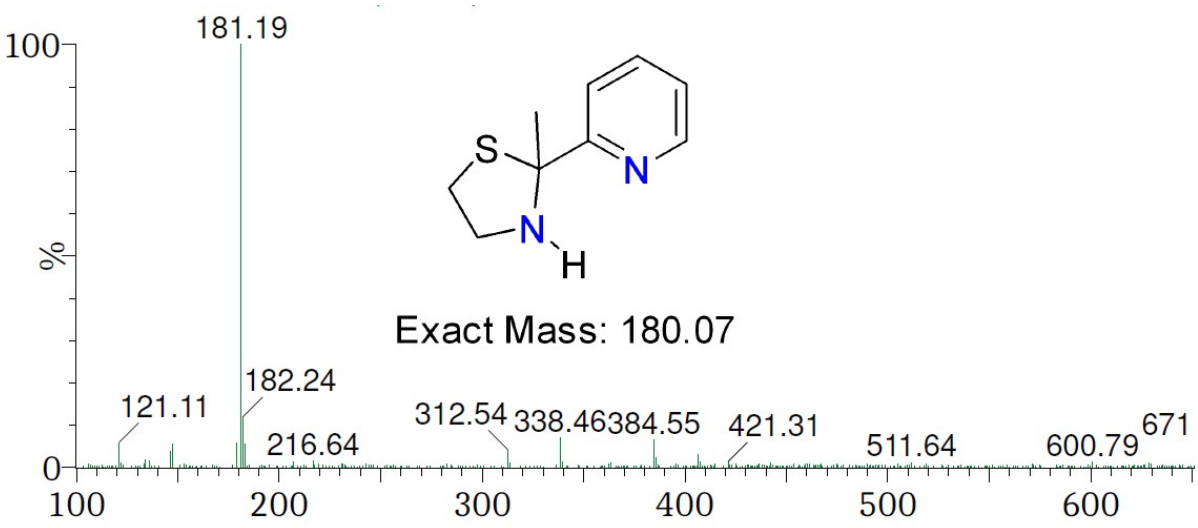

Fig. S10 Mass spectrum of ligand L2

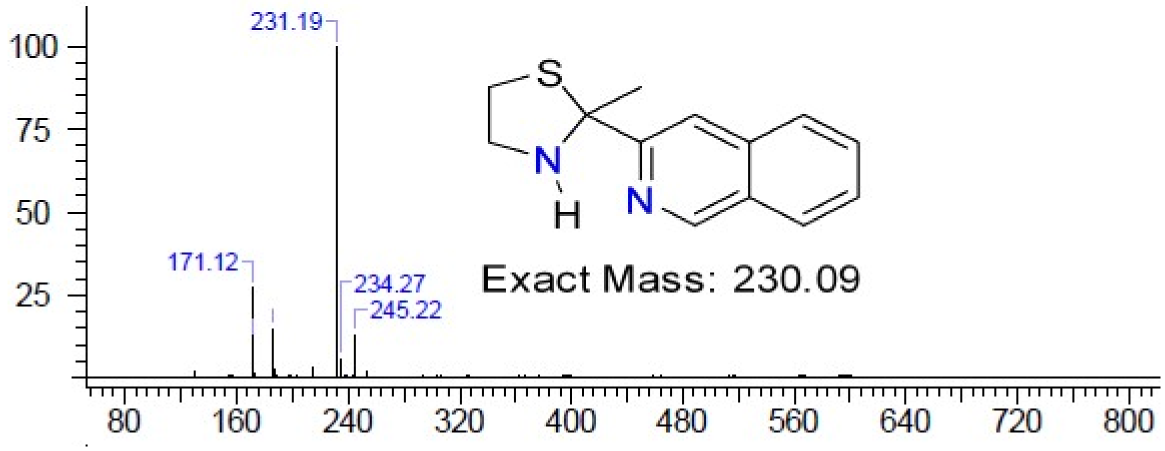

Fig. S11 Mass spectrum of ligand L3

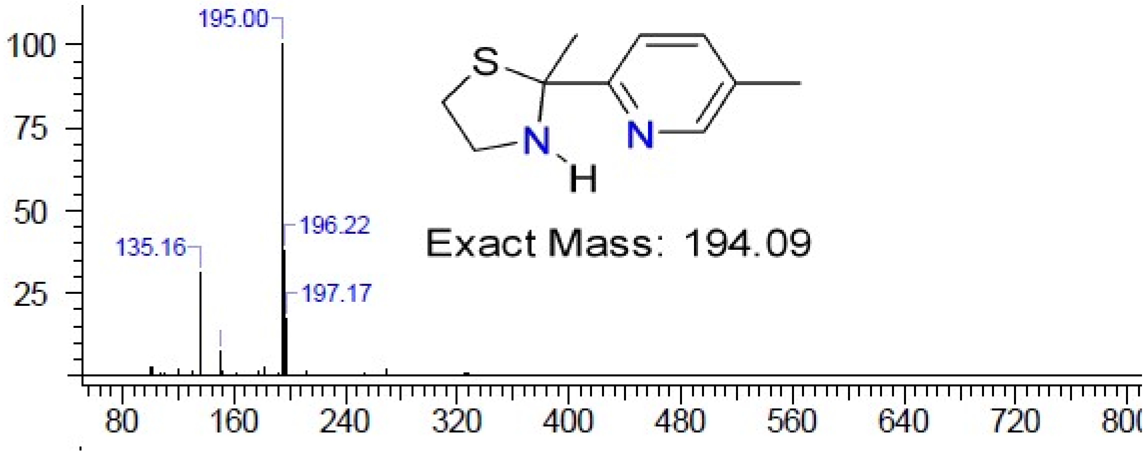

Fig. S12 Mass spectrum of ligand L4

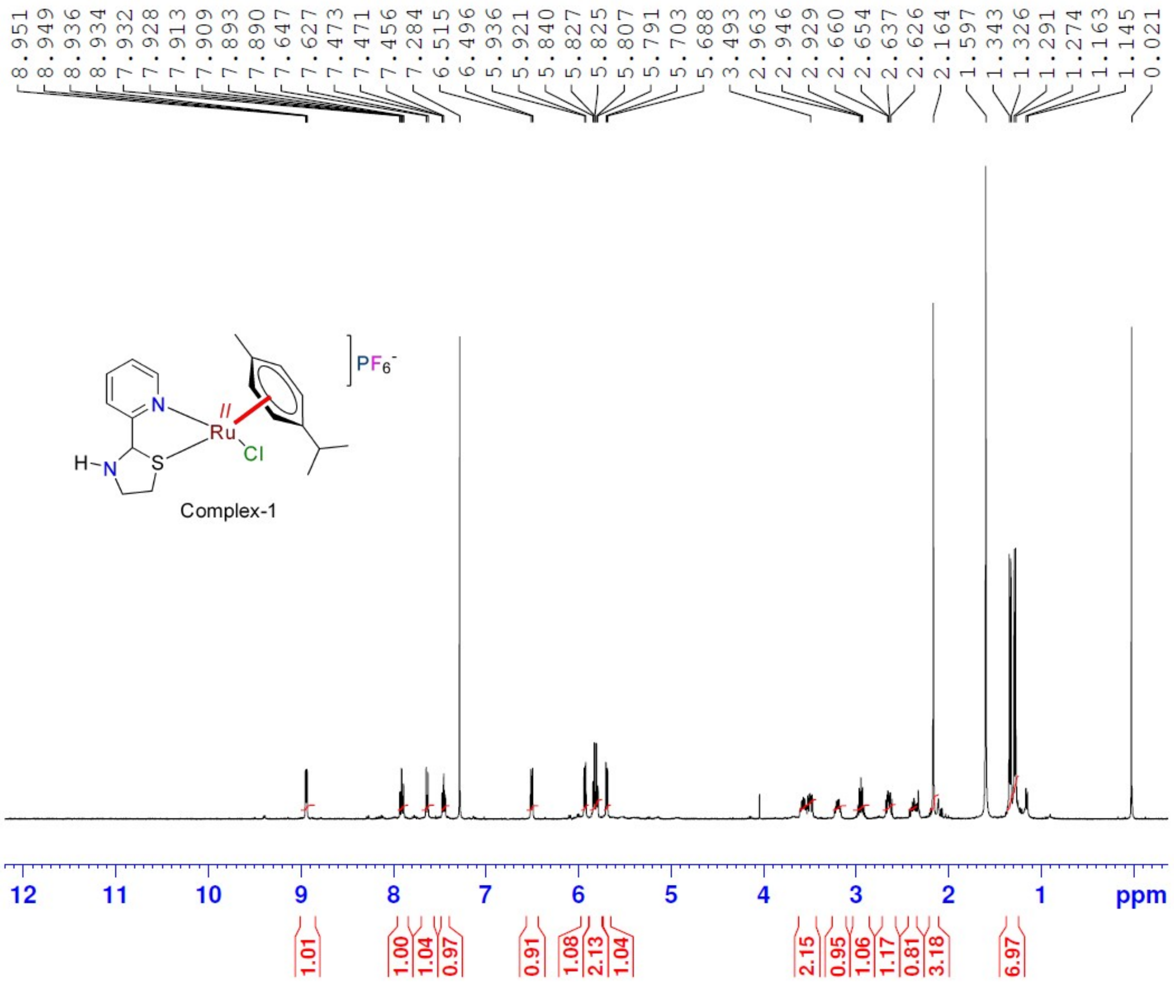

Fig. S13 ^{1}H- NMR spectrum of complex 1

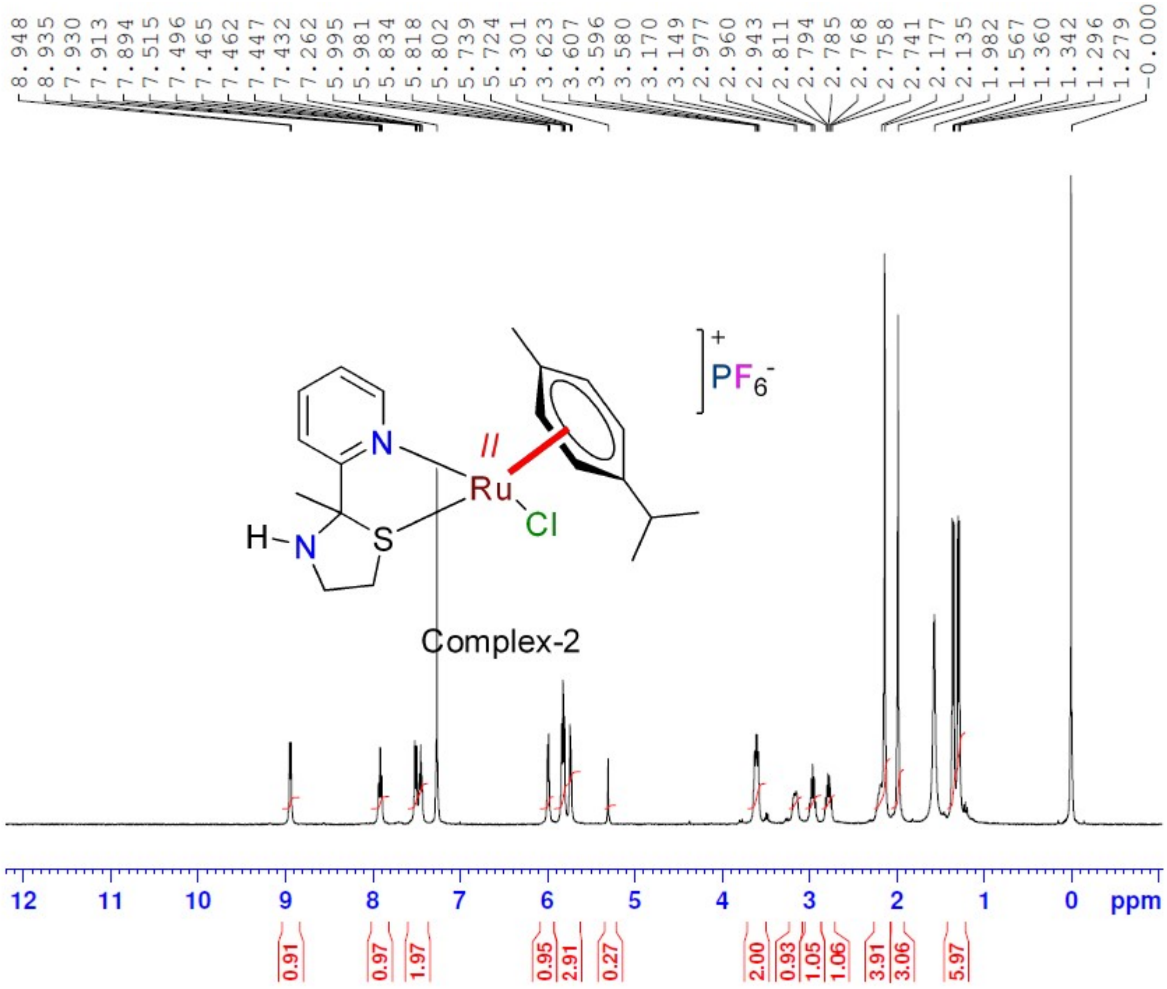

Fig. S14 ^{1}H- NMR spectrum of complex 2

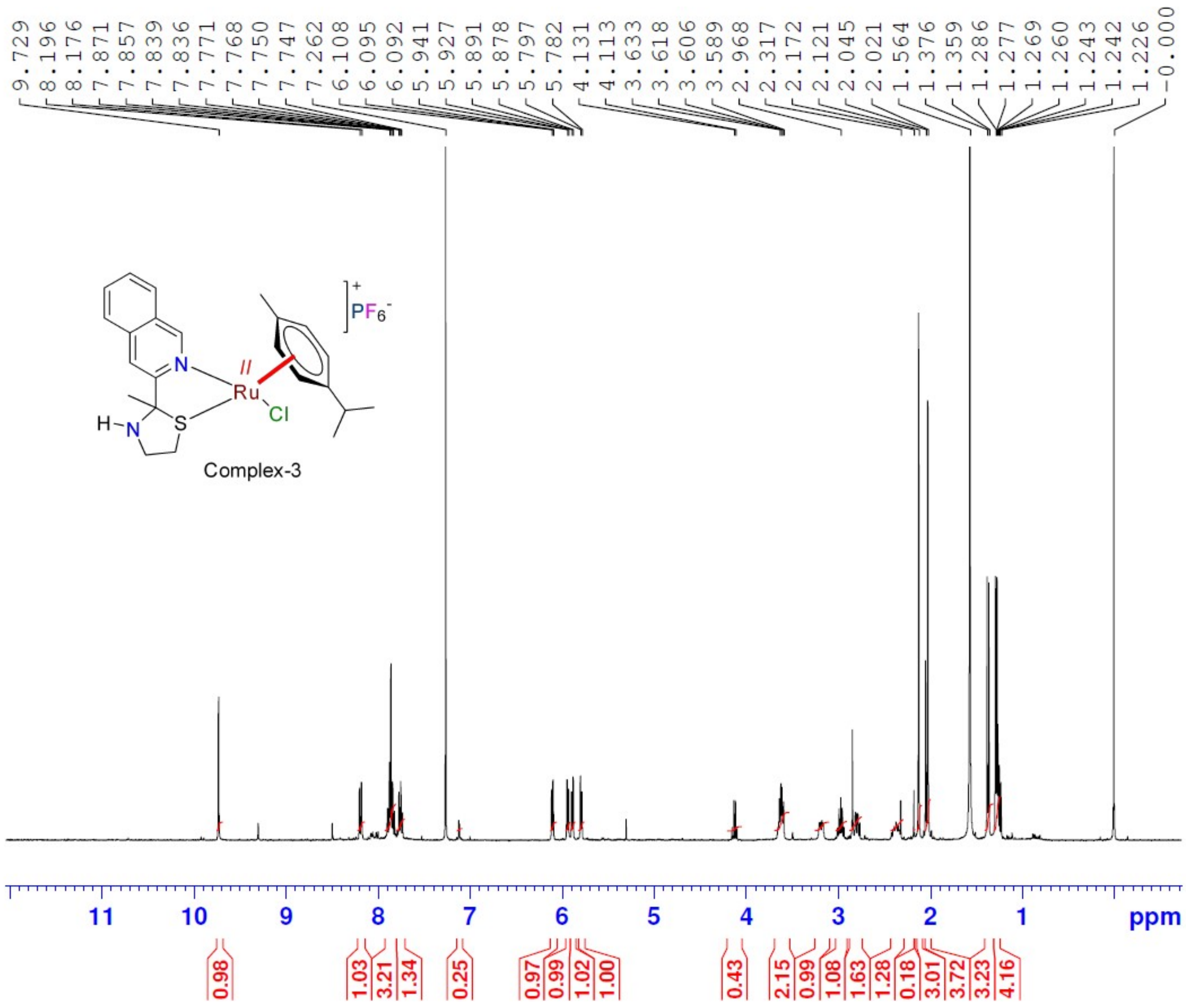

Fig. S15 ^{1}H- NMR spectrum of complex 3

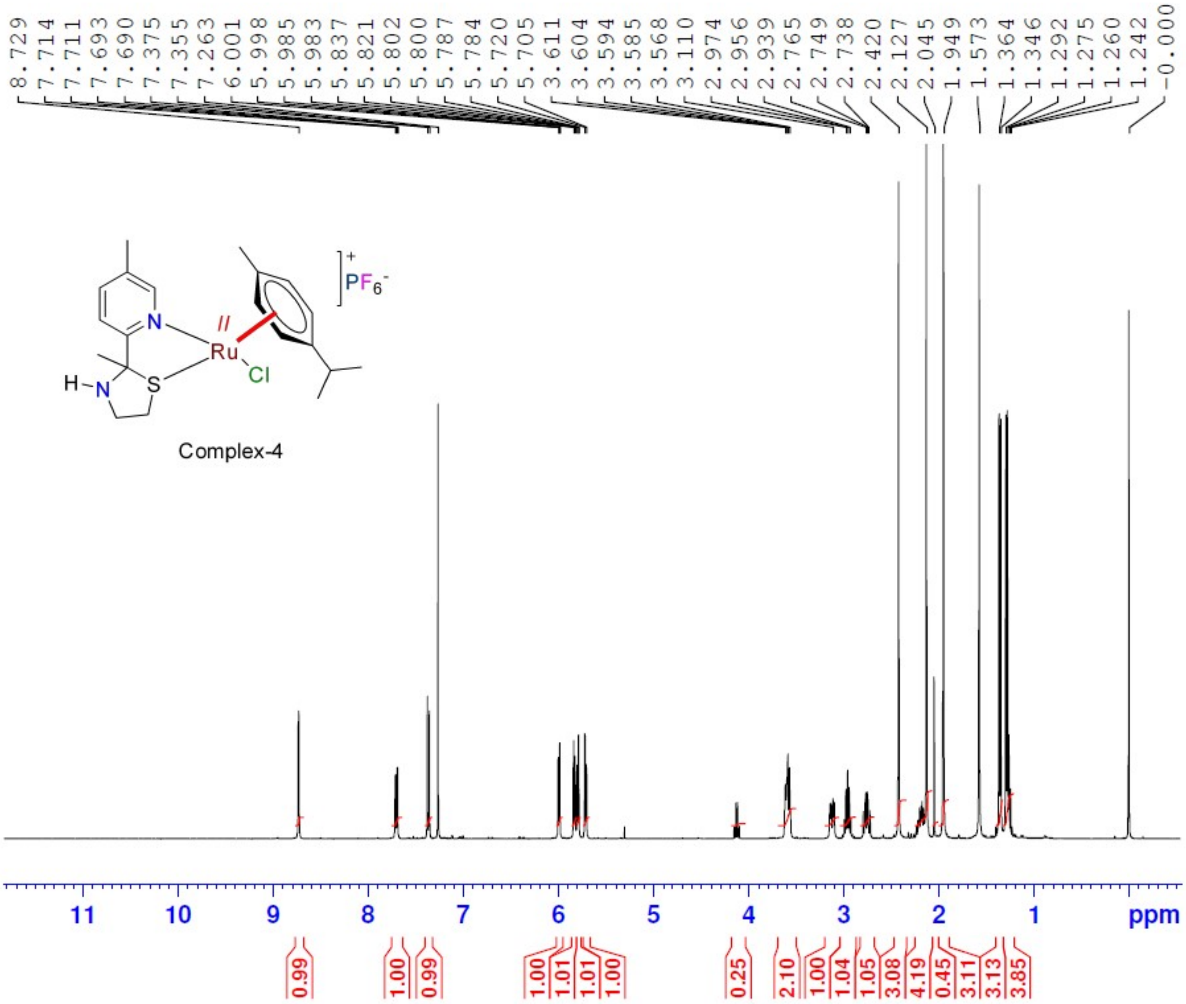

Fig. S16 ^{1}H- NMR spectrum of complex 4

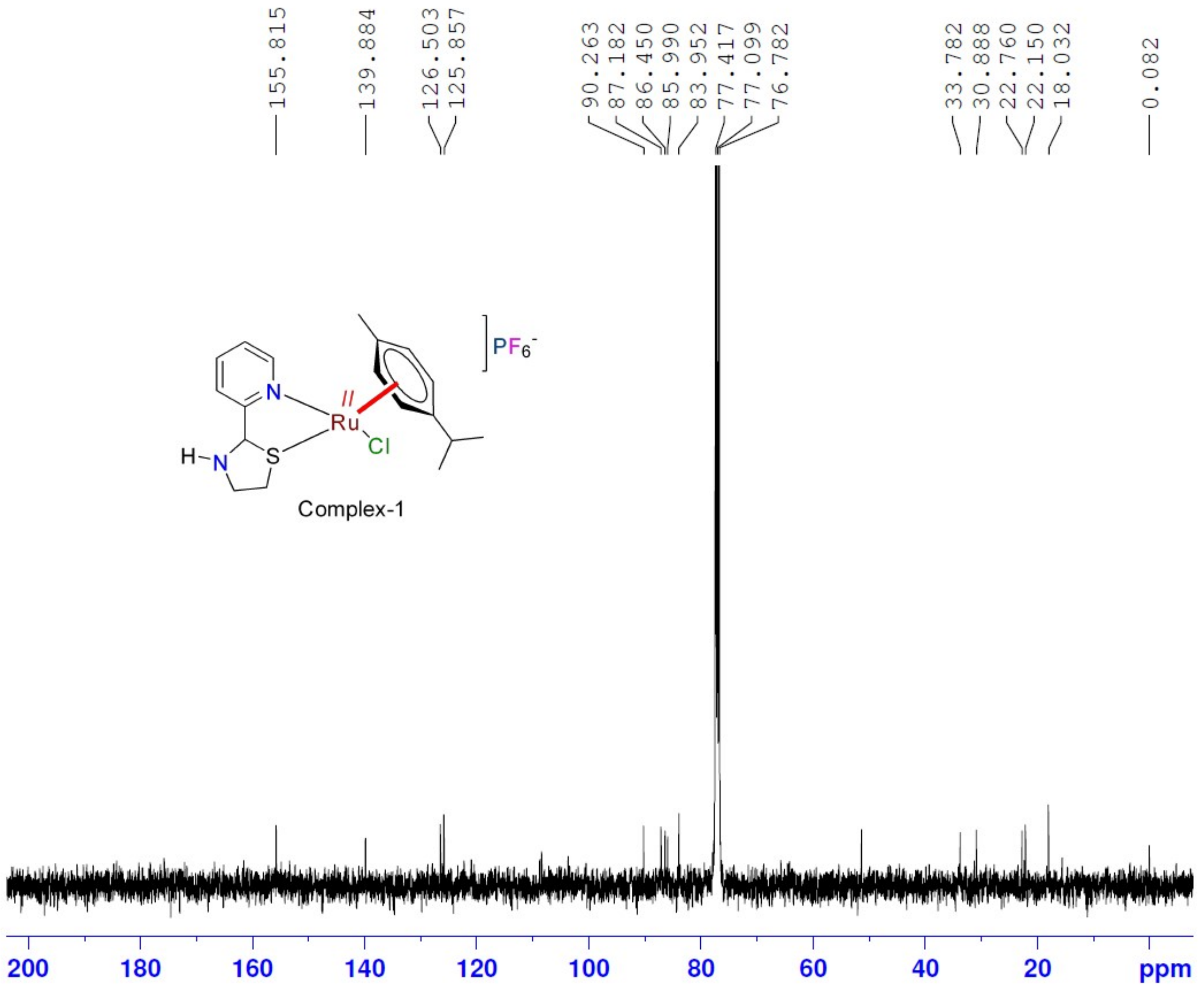

Fig. S17 ^{13}C-NMR spectrum of complex 1

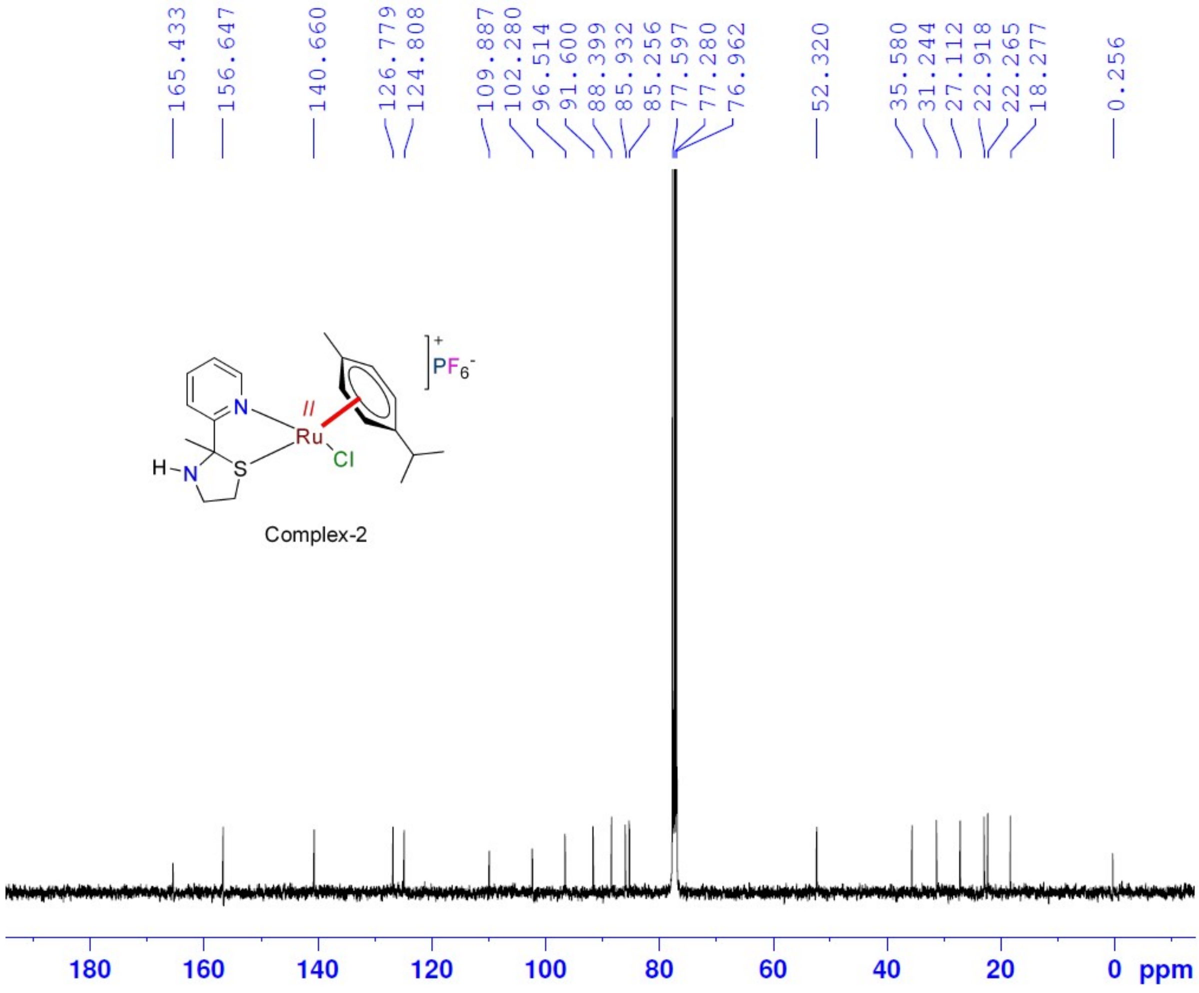

Fig. S18 ^{13}C-NMR spectrum of complex 2

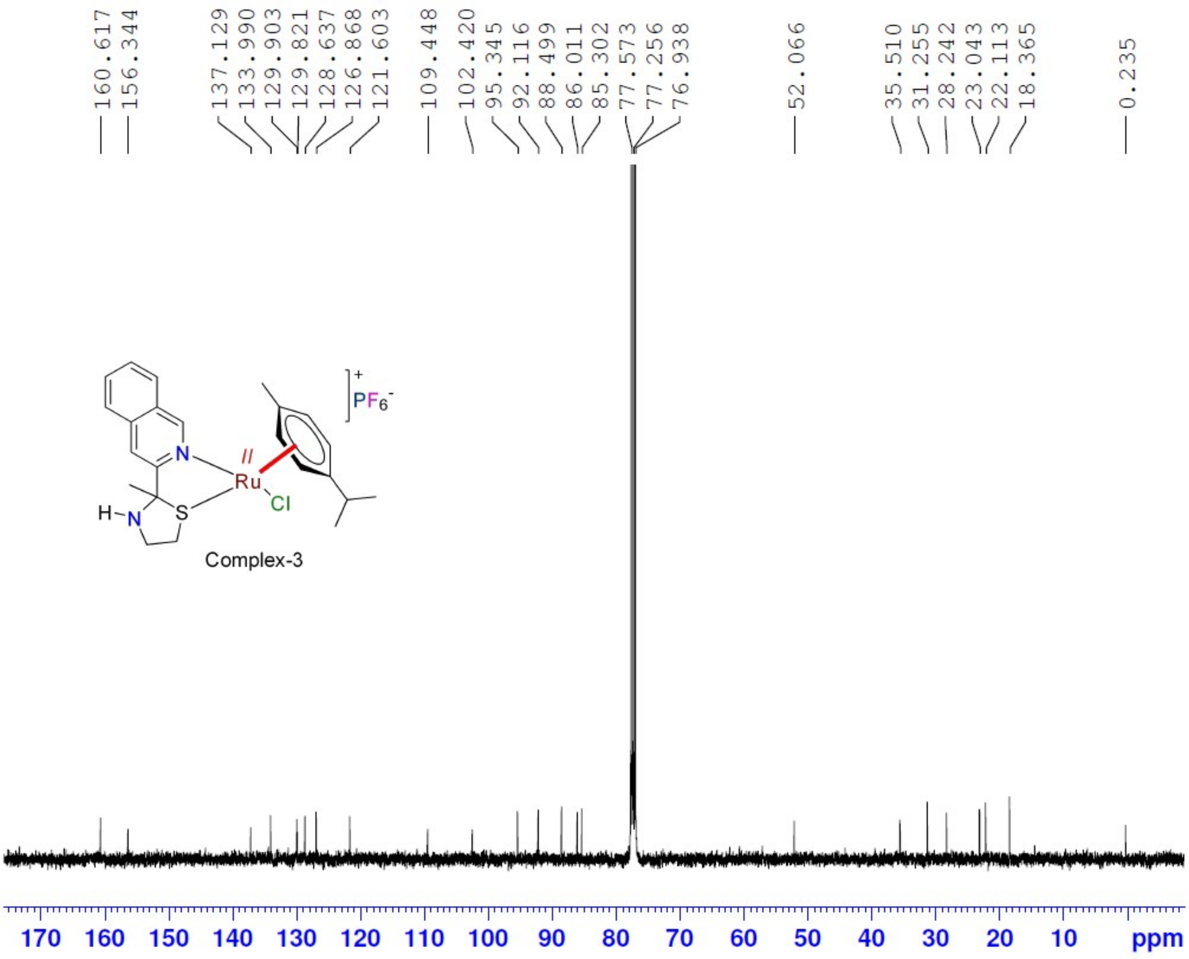

Fig. S19 ^{13}C-NMR spectrum of complex 3

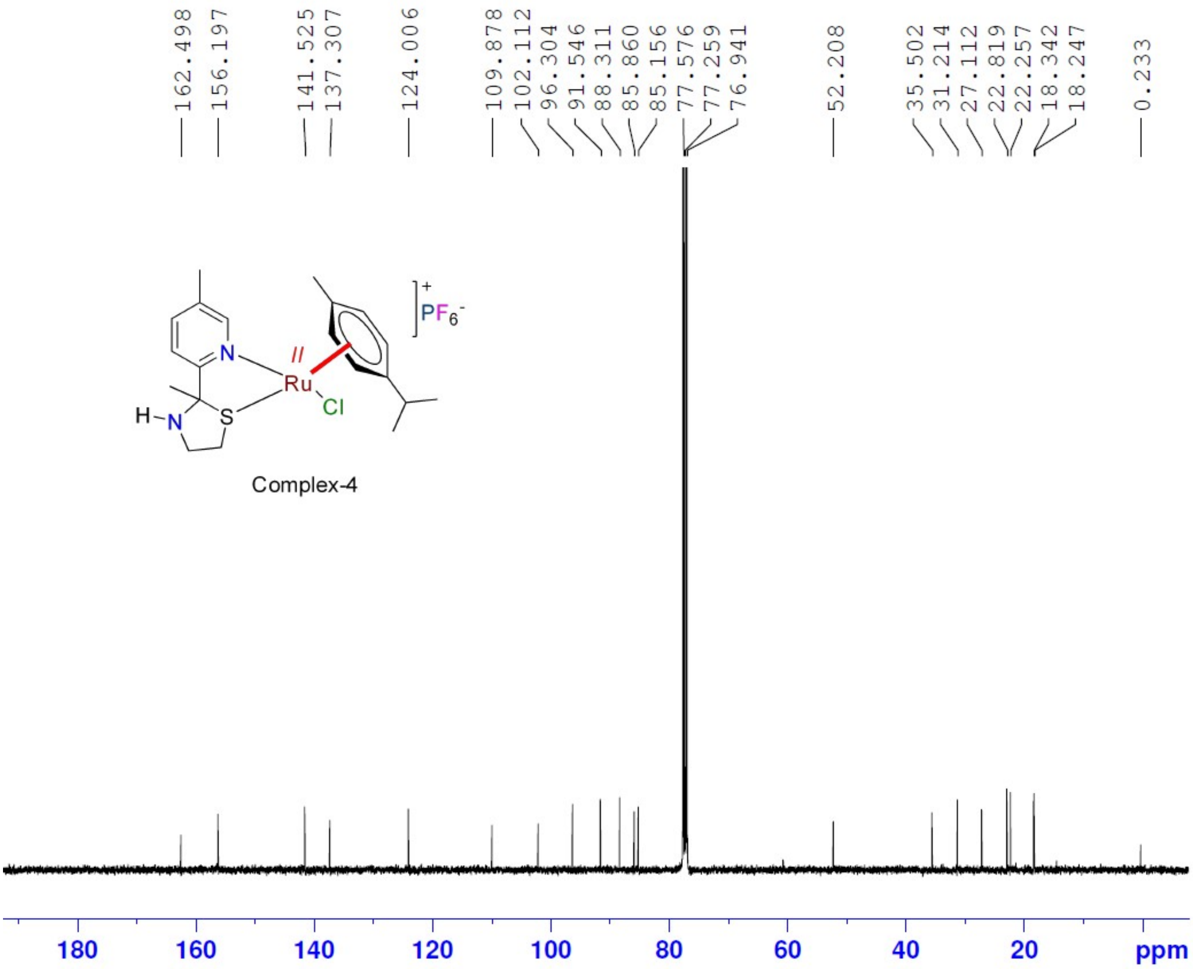

Fig. S20 ^{13}C-NMR spectrum of complex 4

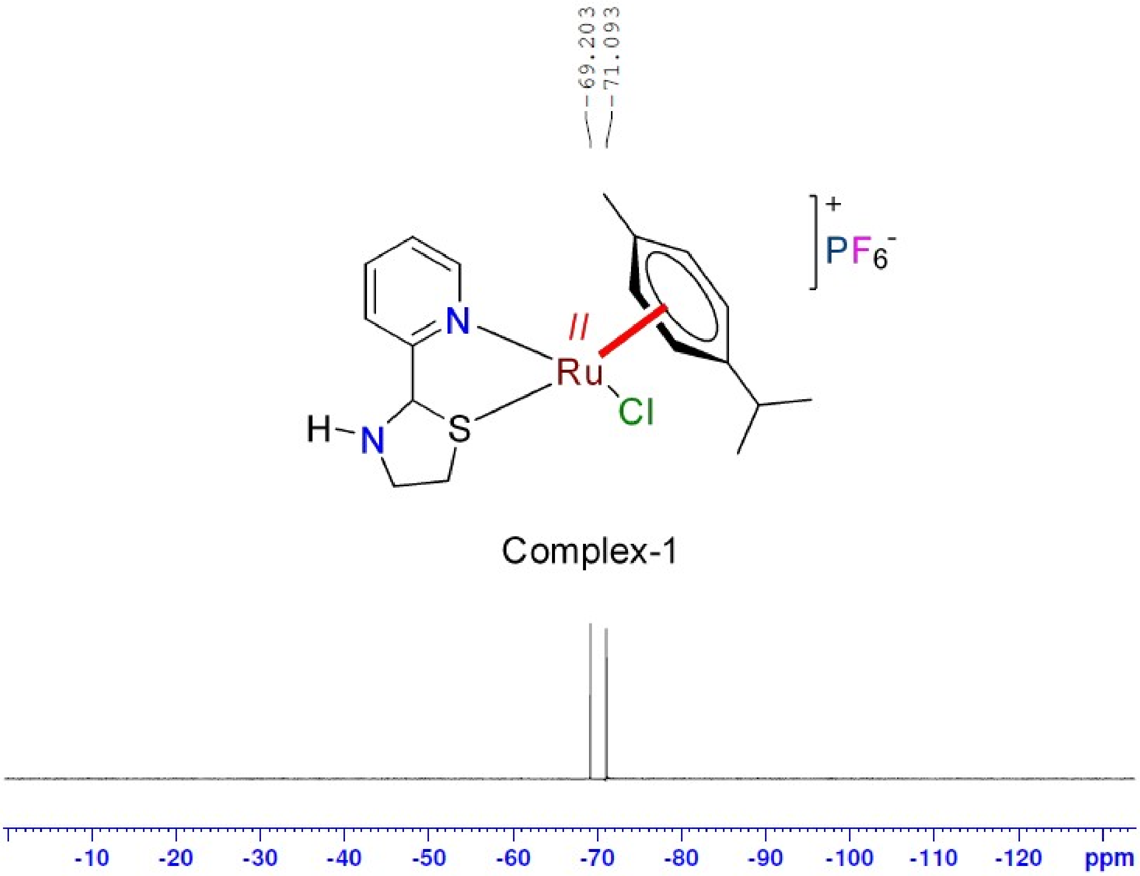

Fig. S21 ^{19}F- NMR spectrum of complex 1

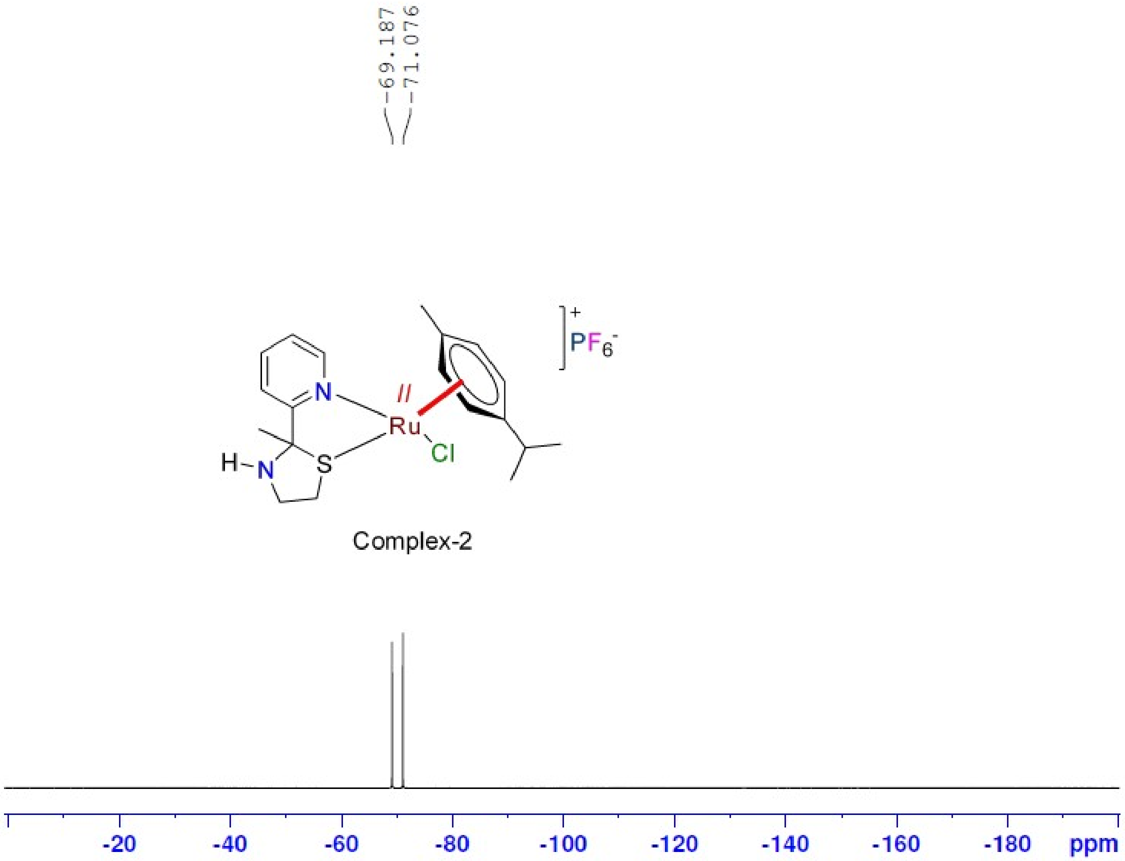

Fig. S22 ^{19}F- NMR spectrum of complex 2

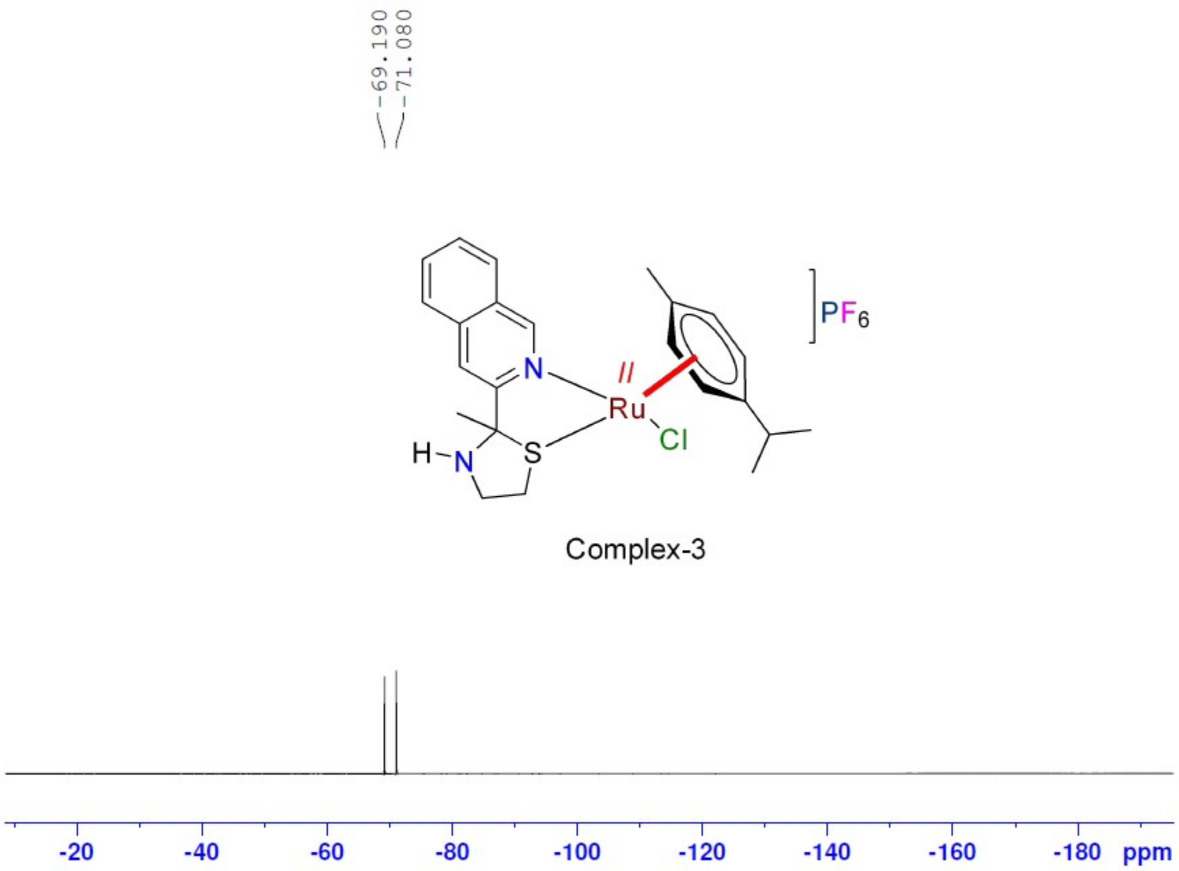

Fig. S23 ^{19}F- NMR spectrum of complex 3

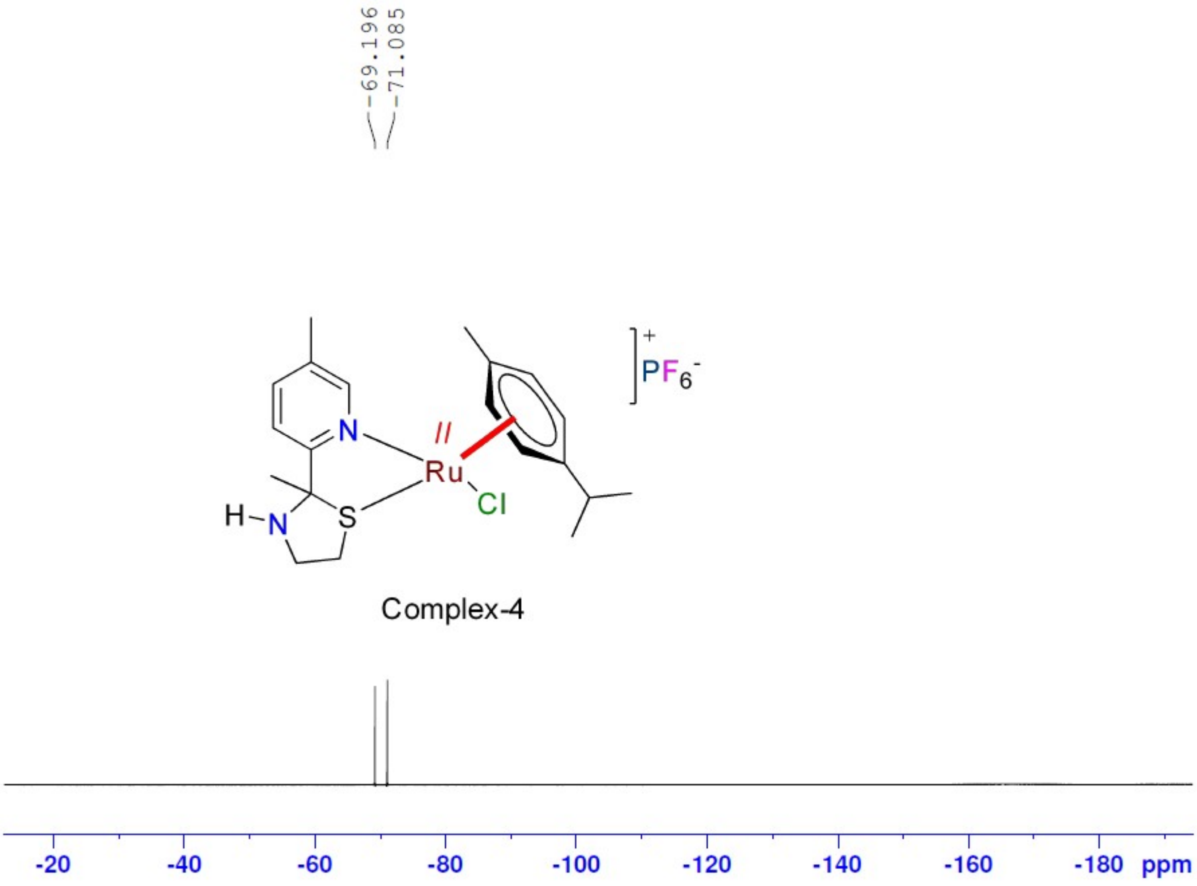

Fig. S24 ^{19}F- NMR spectrum of complex 4

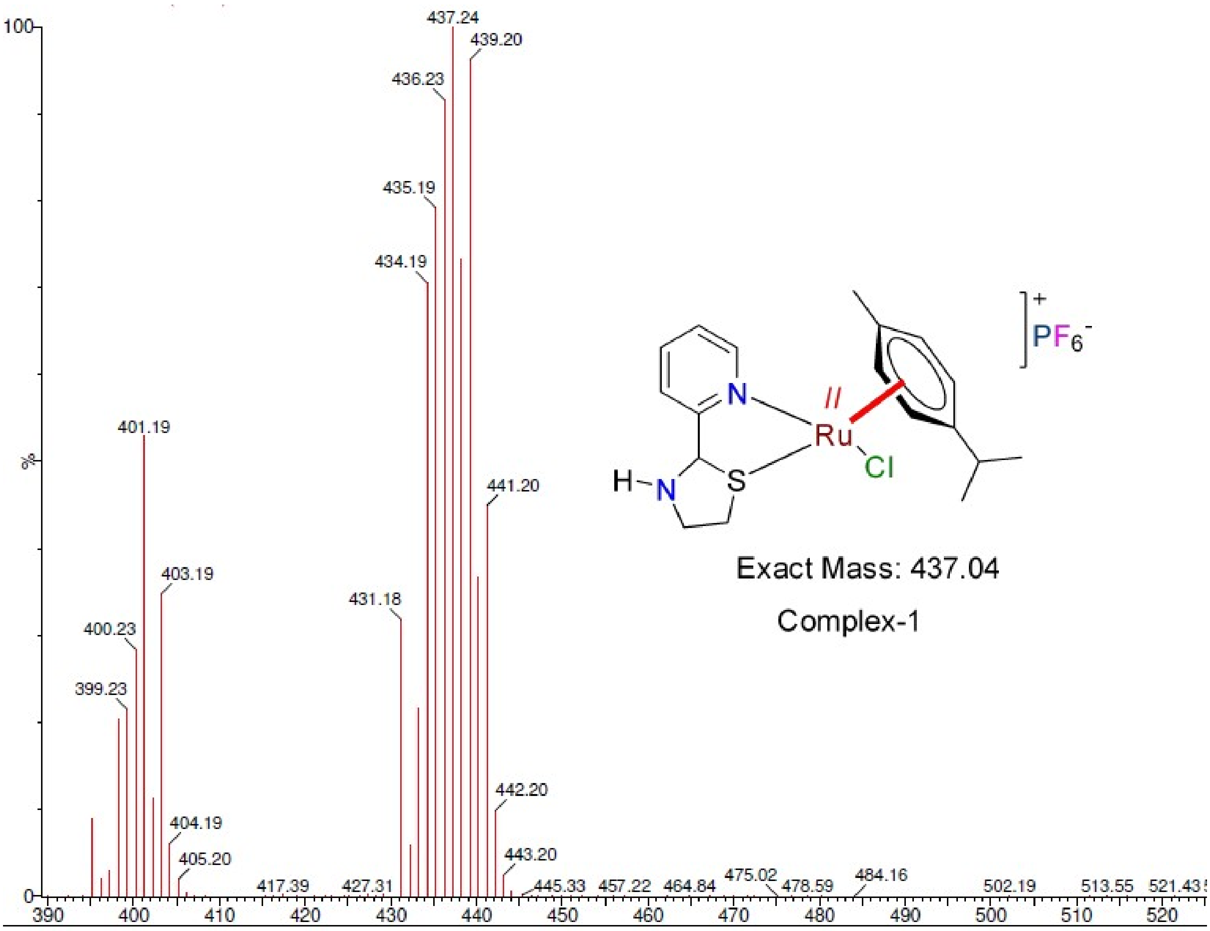

Fig. S25 Mass spectrum of complex 1

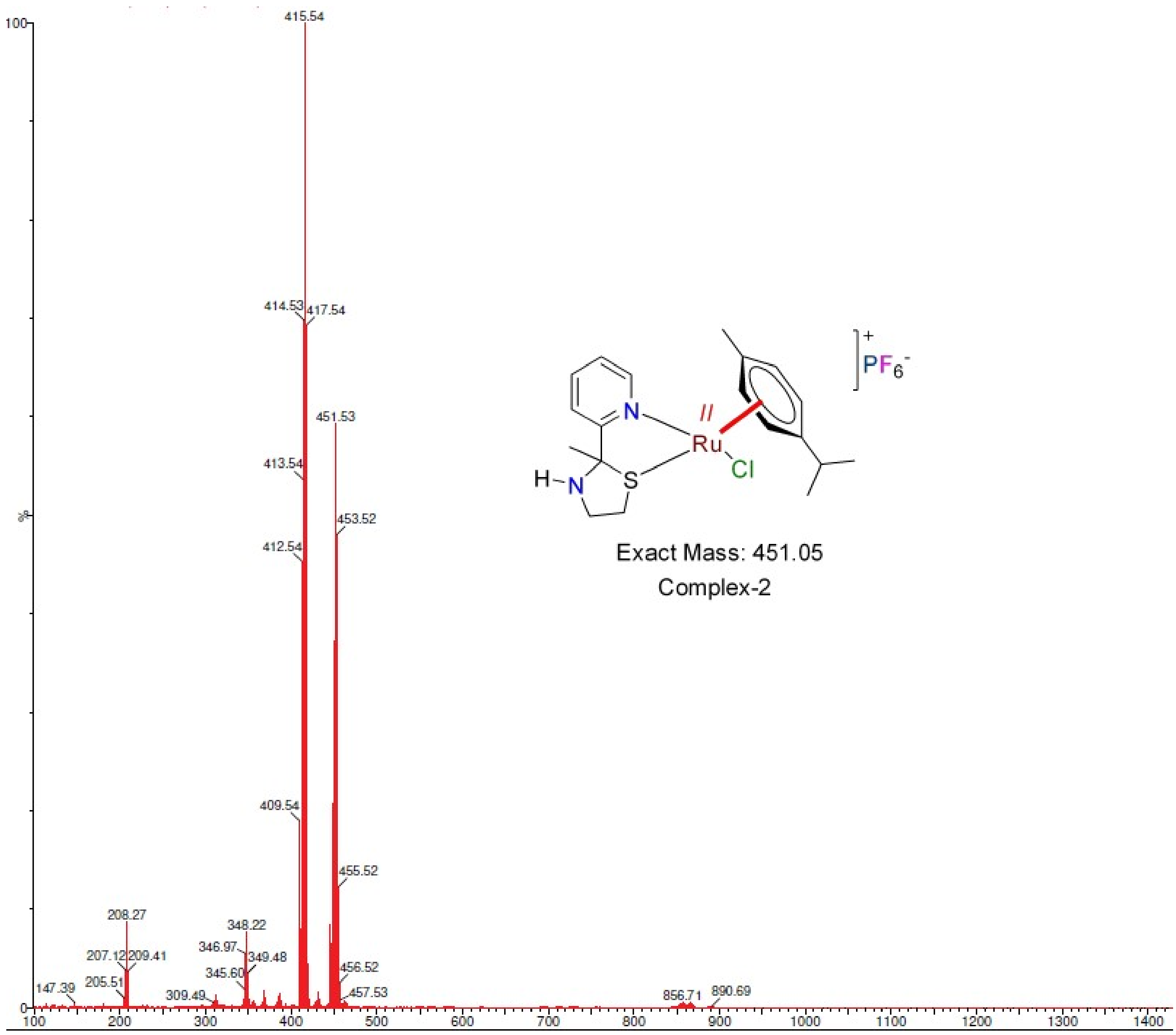

Fig. S26 Mass spectrum of complex 2

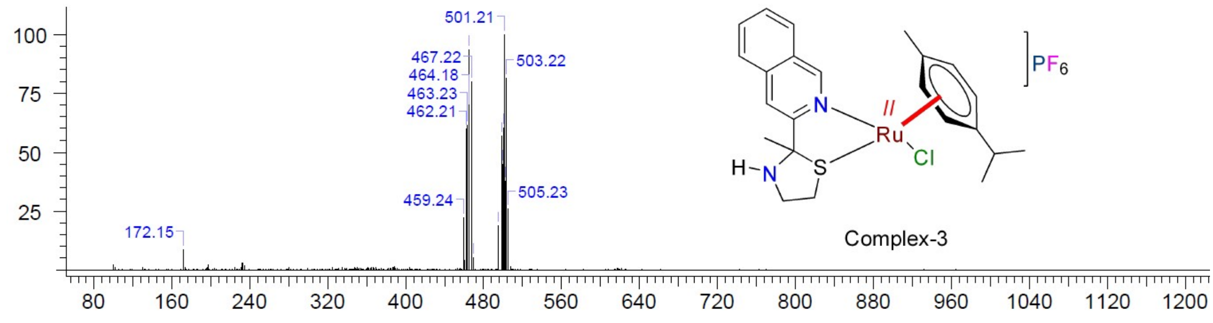

Fig. S27 Mass spectrum of complex 3

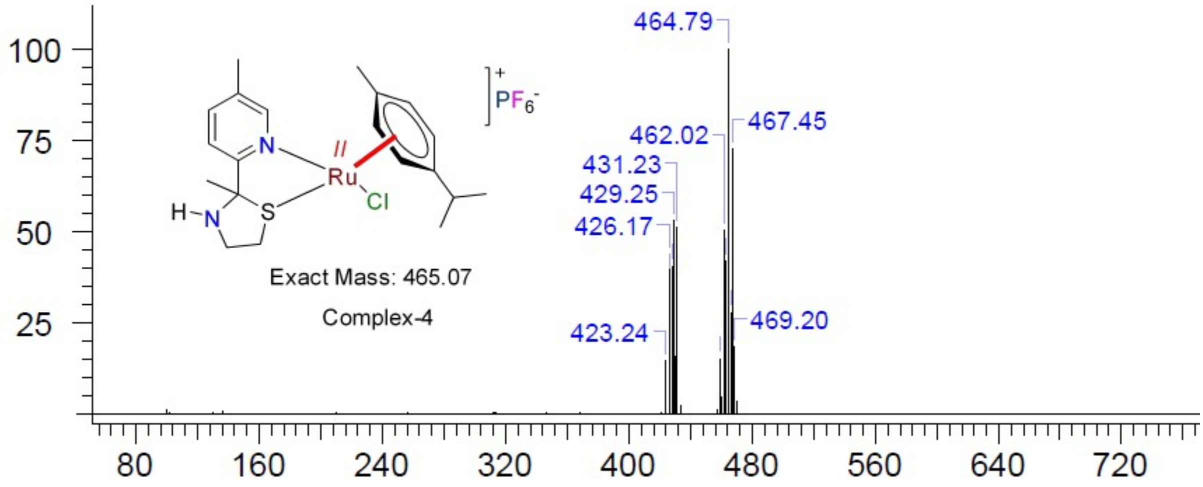

Fig. S28 Mass spectrum of complex 4

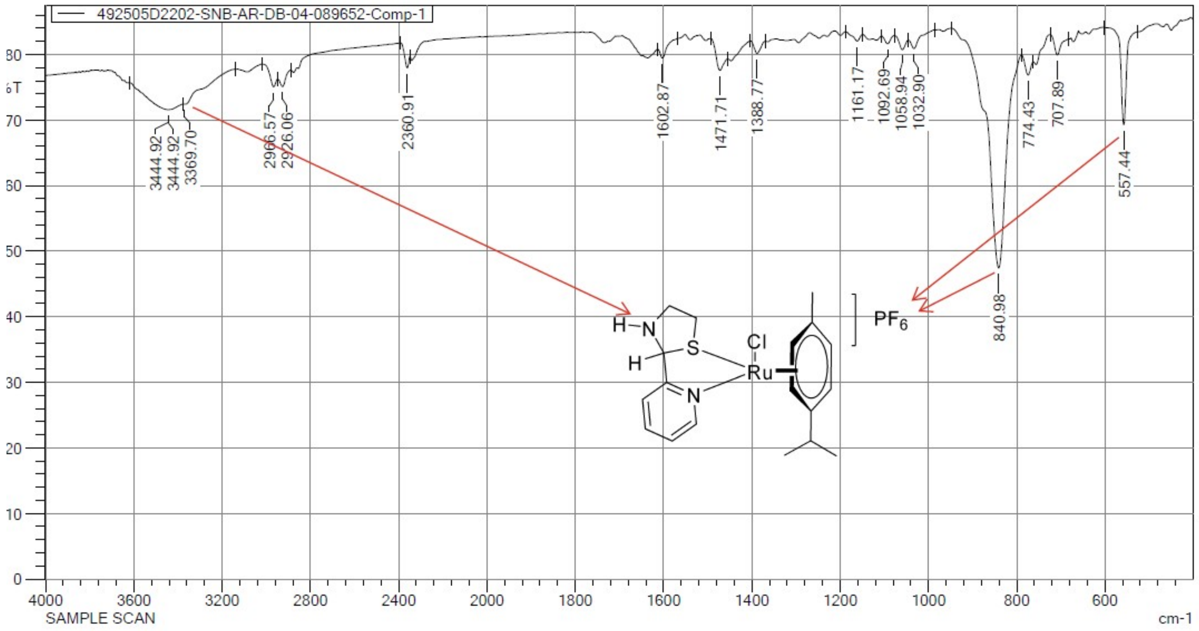

Fig. S29 FTIR spectrum of complex 1

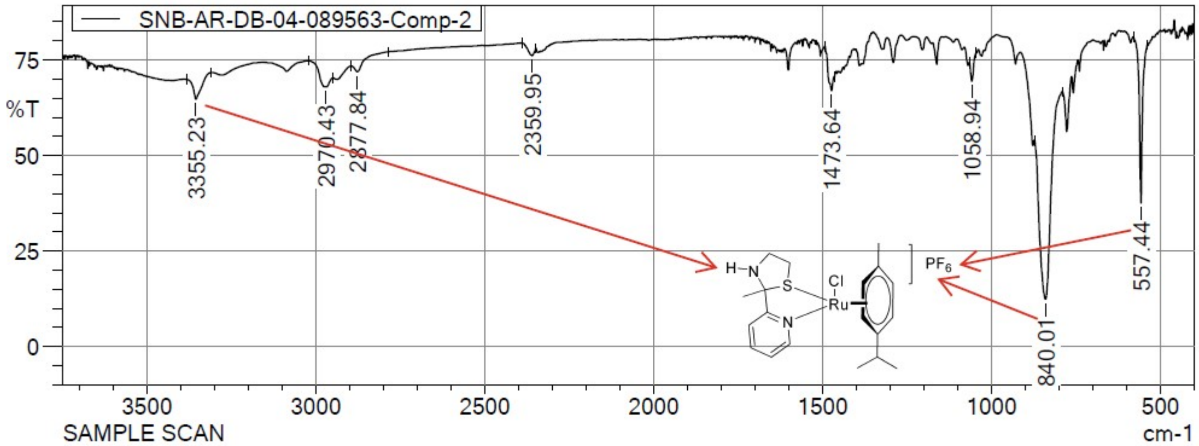

Fig. S30 FTIR spectrum of complex 2

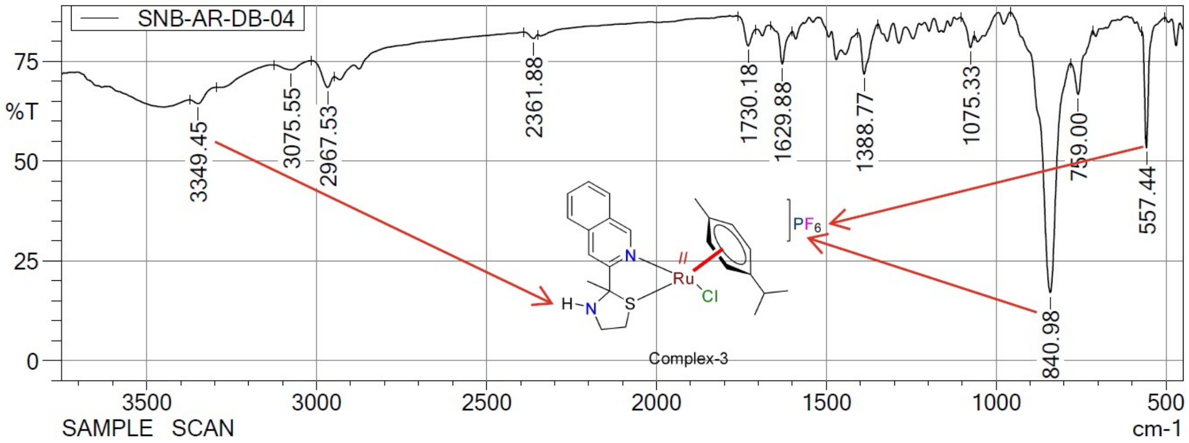

Fig. S31 FTIR spectrum of complex 3

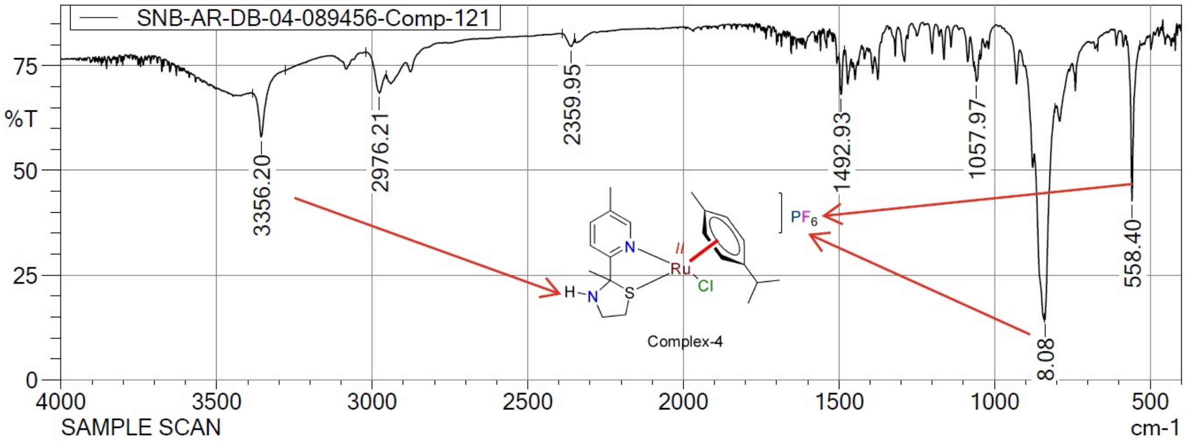

Fig. S32 FTIR spectrum of complex 4

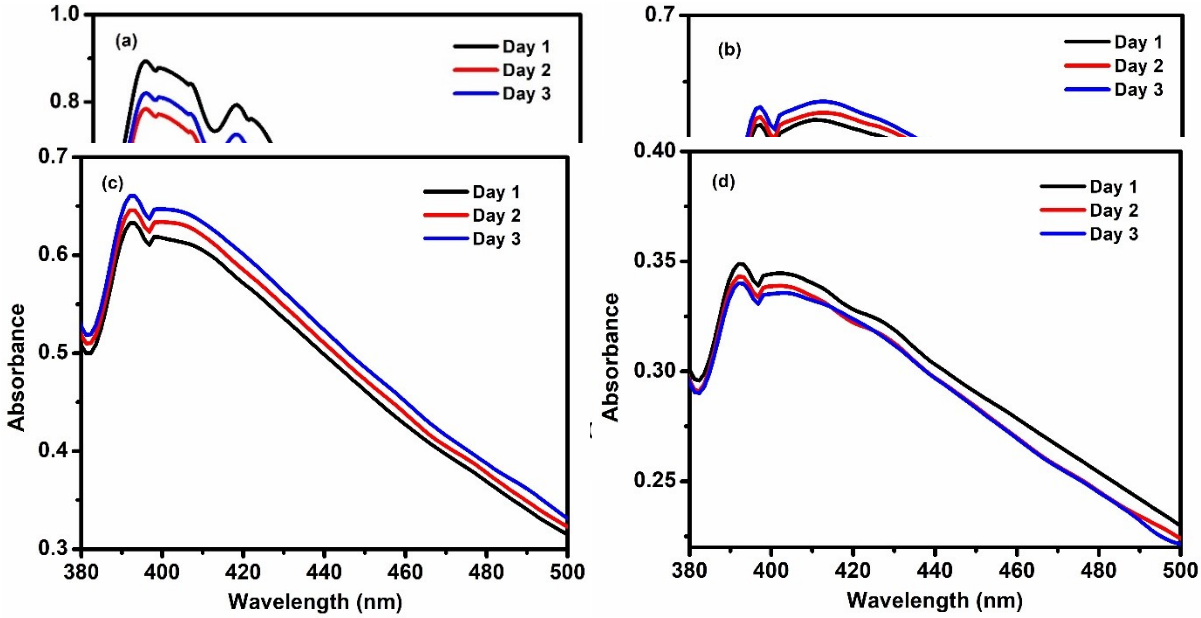

Fig. S33 UV-Vis spectral changes of pure (a) complex 1 and (b) complex 2 (c) complex 3 and (d) complex 4 with consecutive three days

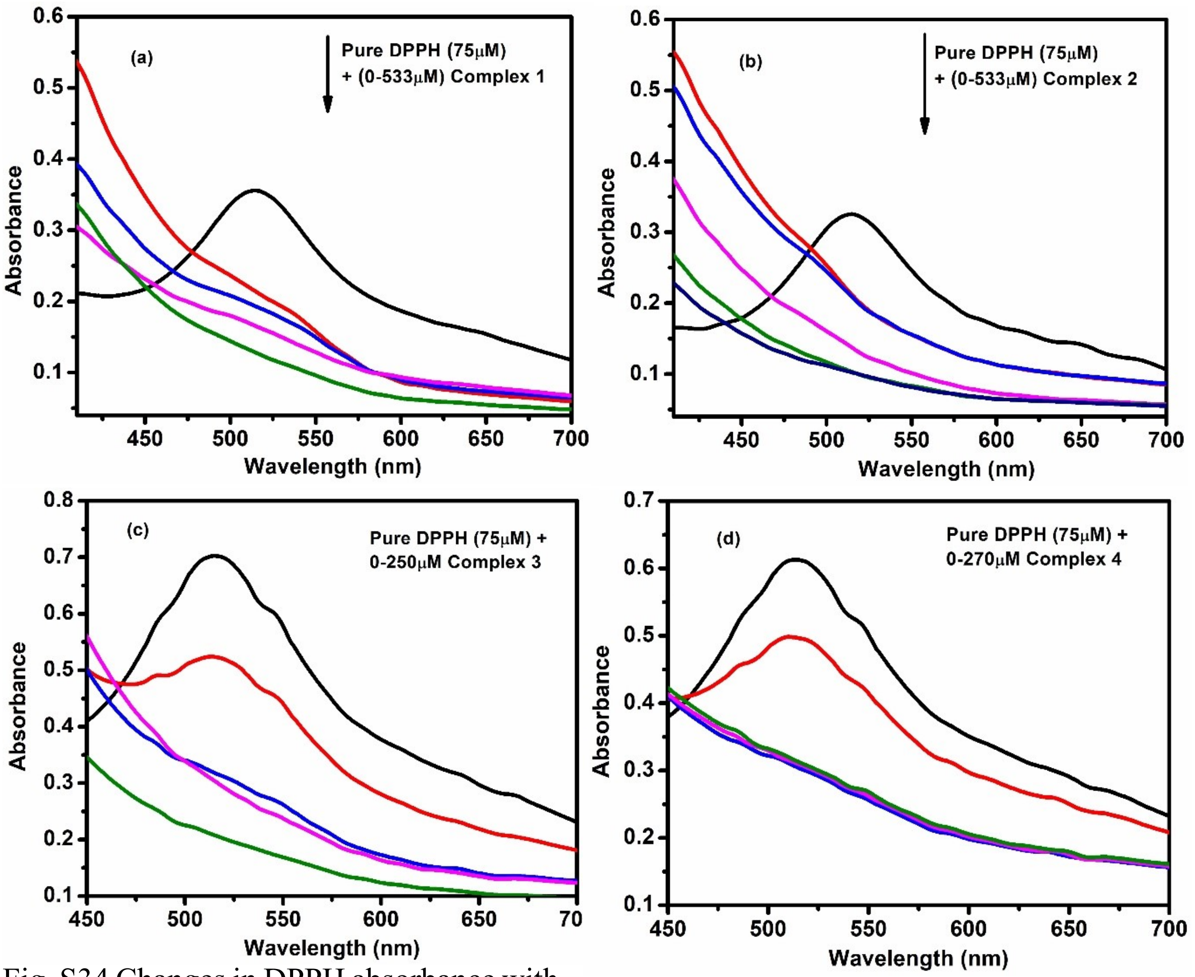

Fig. S34 Changes in DPPH absorbance with addition of (a) complex 1 (b) complex 2 (c) complex 3 (d) complex 4

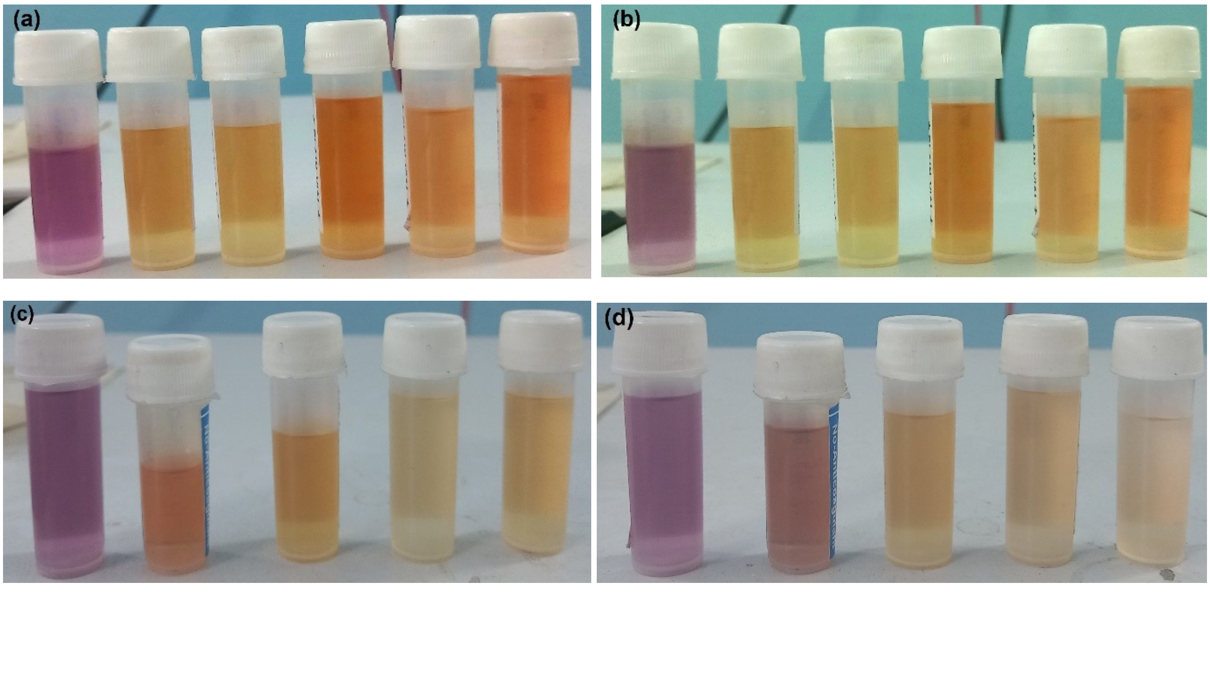

Fig. S35 Colour changes of pure DPPH solution with gradual addition of (a) complex 1 (b) complex 2 (c) complex 3 (d) complex 4

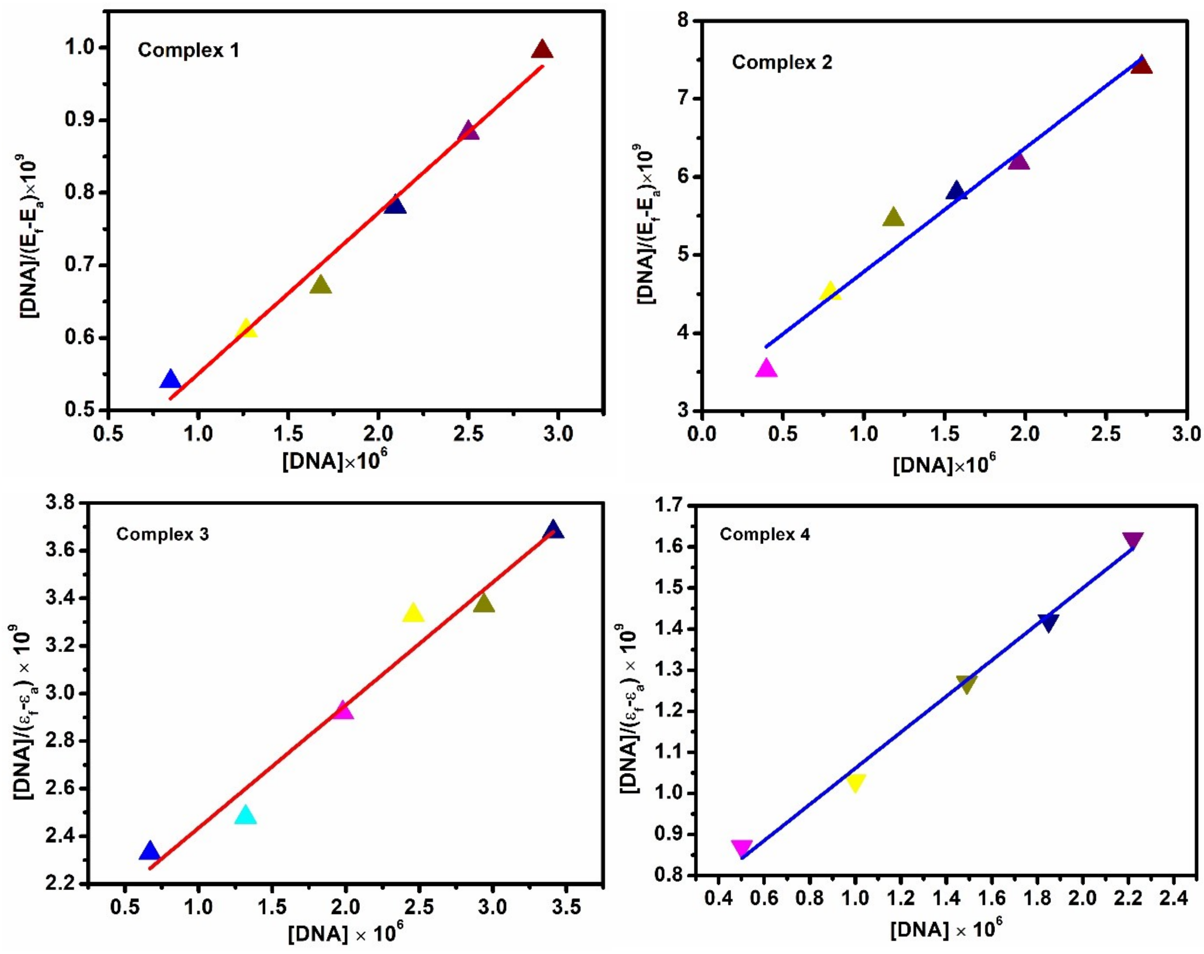

Fig. S36 Wolfe–Shimmer plots for complex 1, 2, 3 and 4.

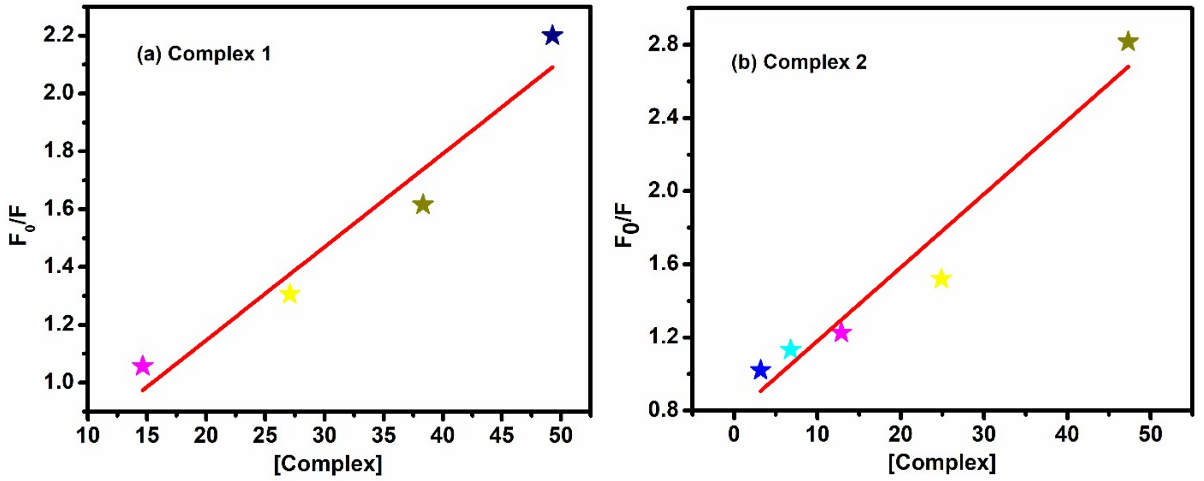

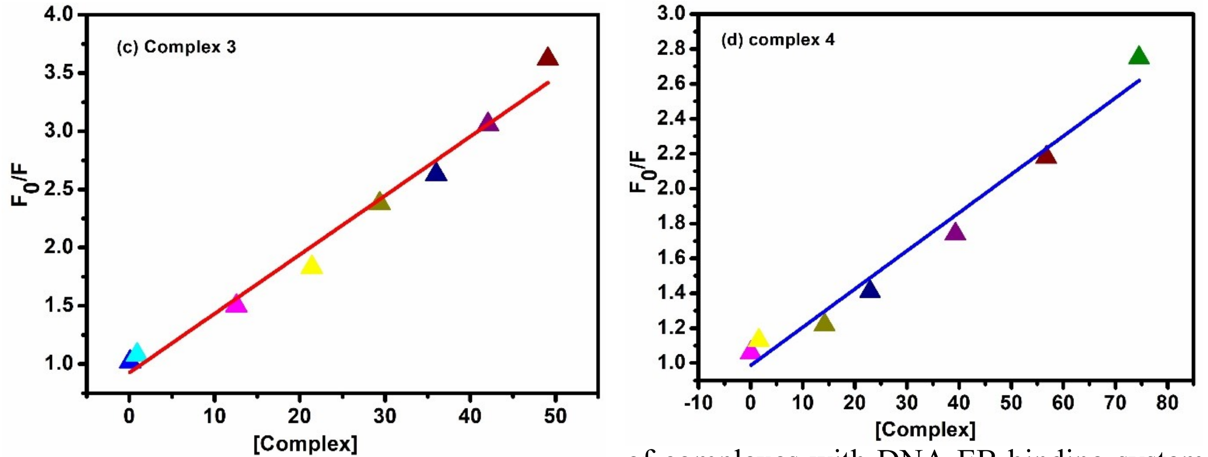

Fig. S37 Stern Volmer plots for the interaction of complexes with DNA-EB binding system (a) complex 1 (b) complex 2 (c) complex 3 and (d) complex 4

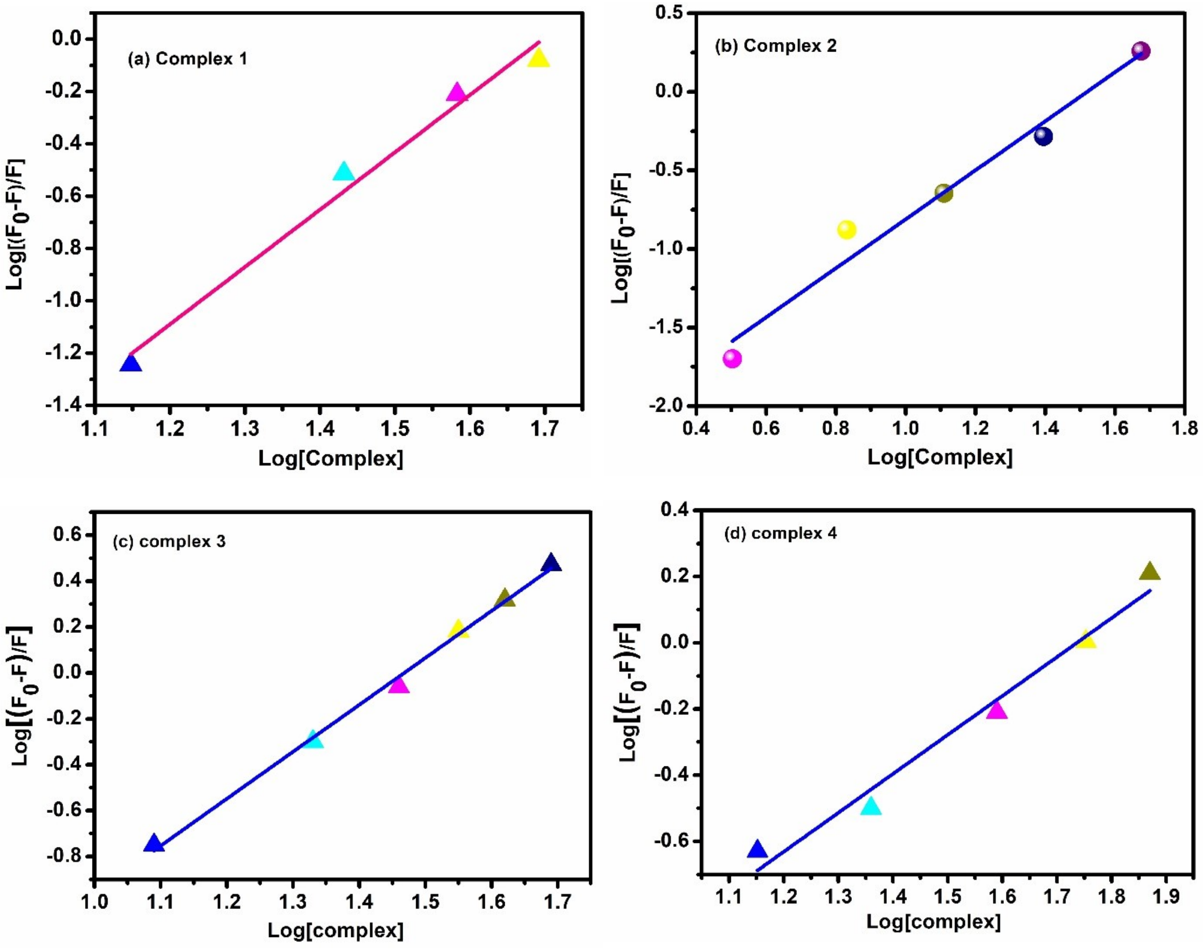

Fig. S38 Scatchard plots for the interaction of complexes with DNA-EB binding system (a) complex 1 (b) complex 2 (c) complex 3 and (d) complex 4

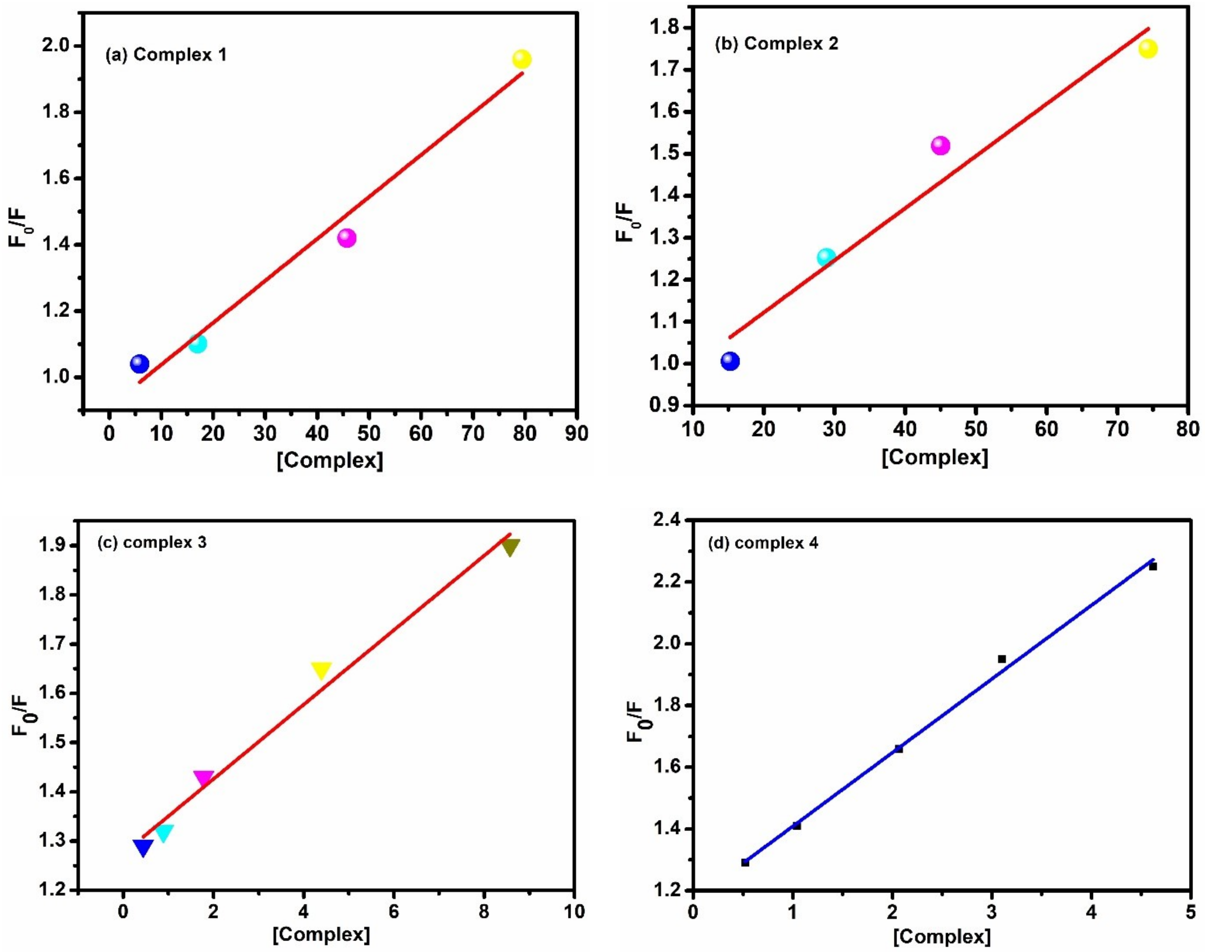

Fig. S39 Stern Volmer plots for the interaction of complexes with DNA-Hoechst binding system (a) complex 1 (b) complex 2 (c) complex 3 and (d) complex 4

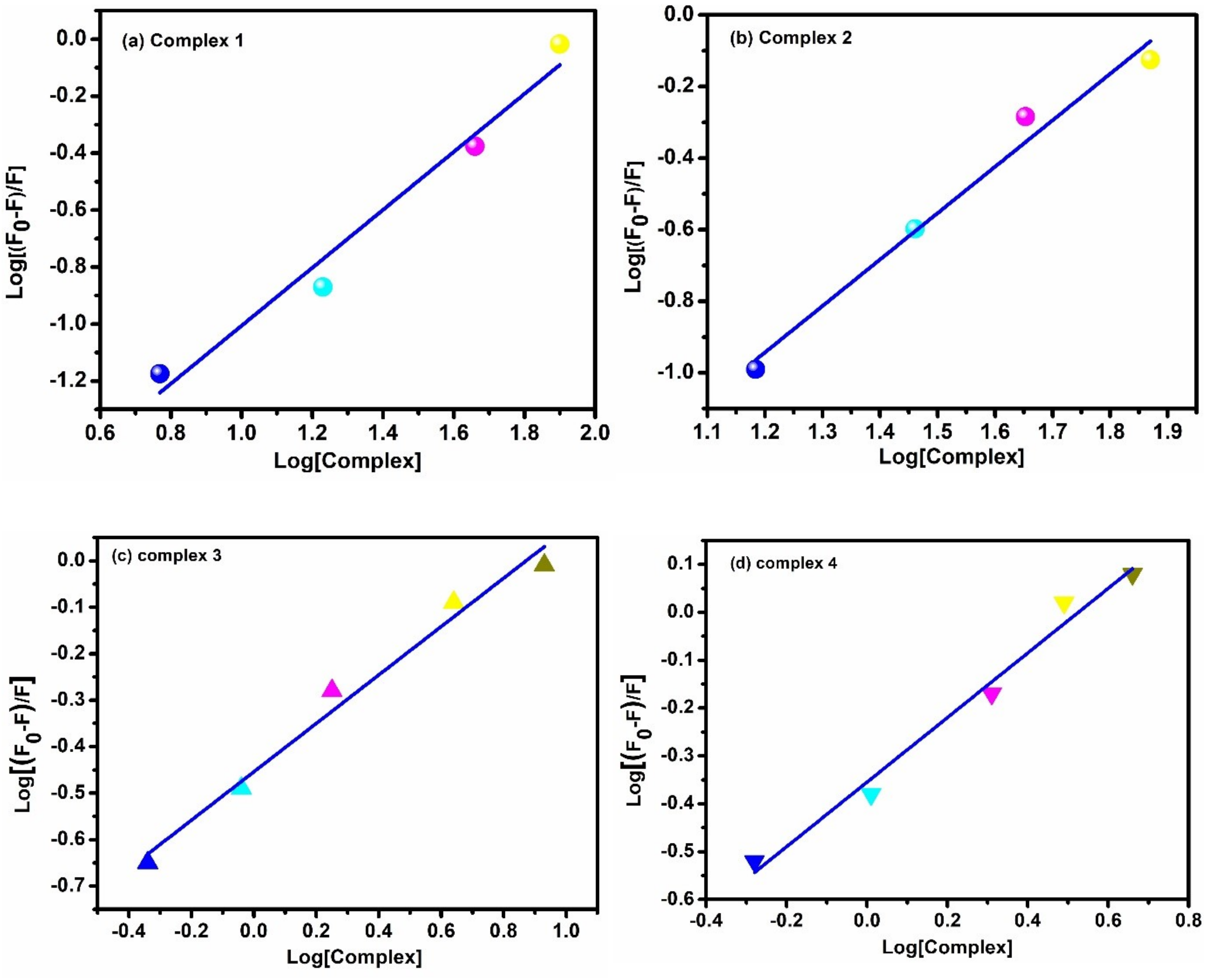

Fig. S40 Scatchard plot for the interaction of complexes with DNA-Hoechst binding system (a) complex 1 (b) complex 2 (c) complex 3 and (d) complex 4

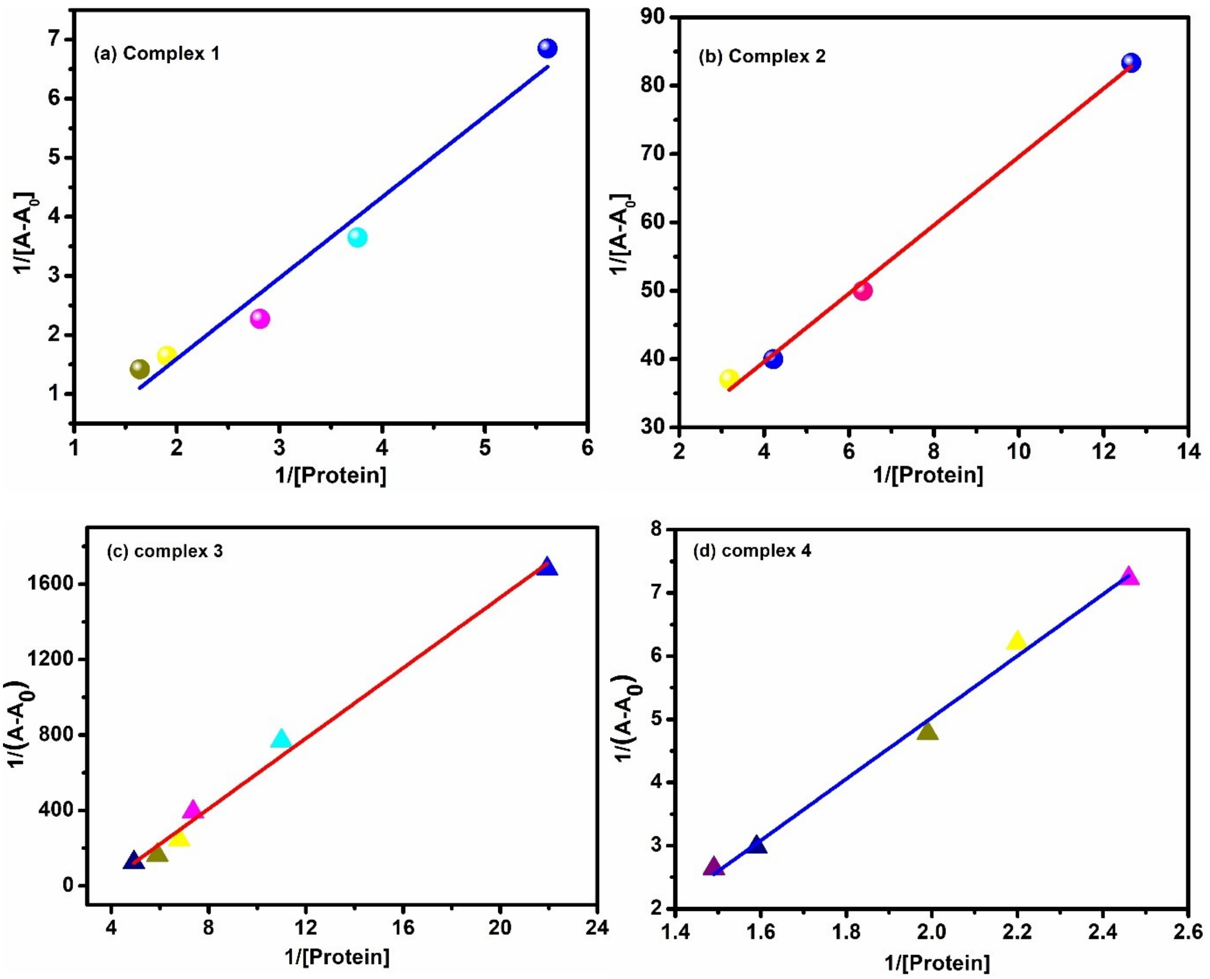

Fig. S41 Apparent binding constant plots for BSA binding interaction of (a) complex 1 (b) complex 2 (c) complex 3 (d) complex 4

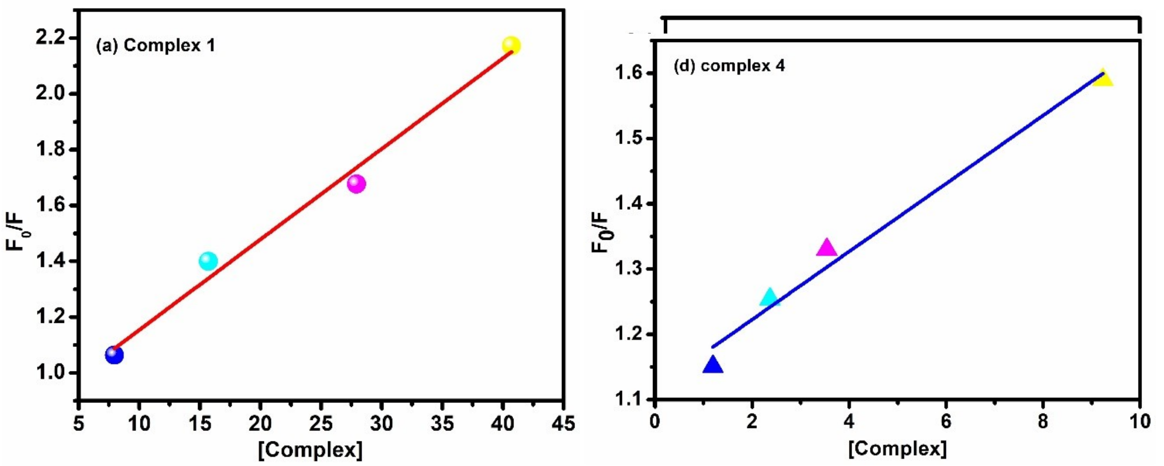

Fig. S42 Stern Volmer plots for the interaction of complexes with BSA binding system (a) complex 1 (b) complex 2 (c) complex 3 (d) complex 4

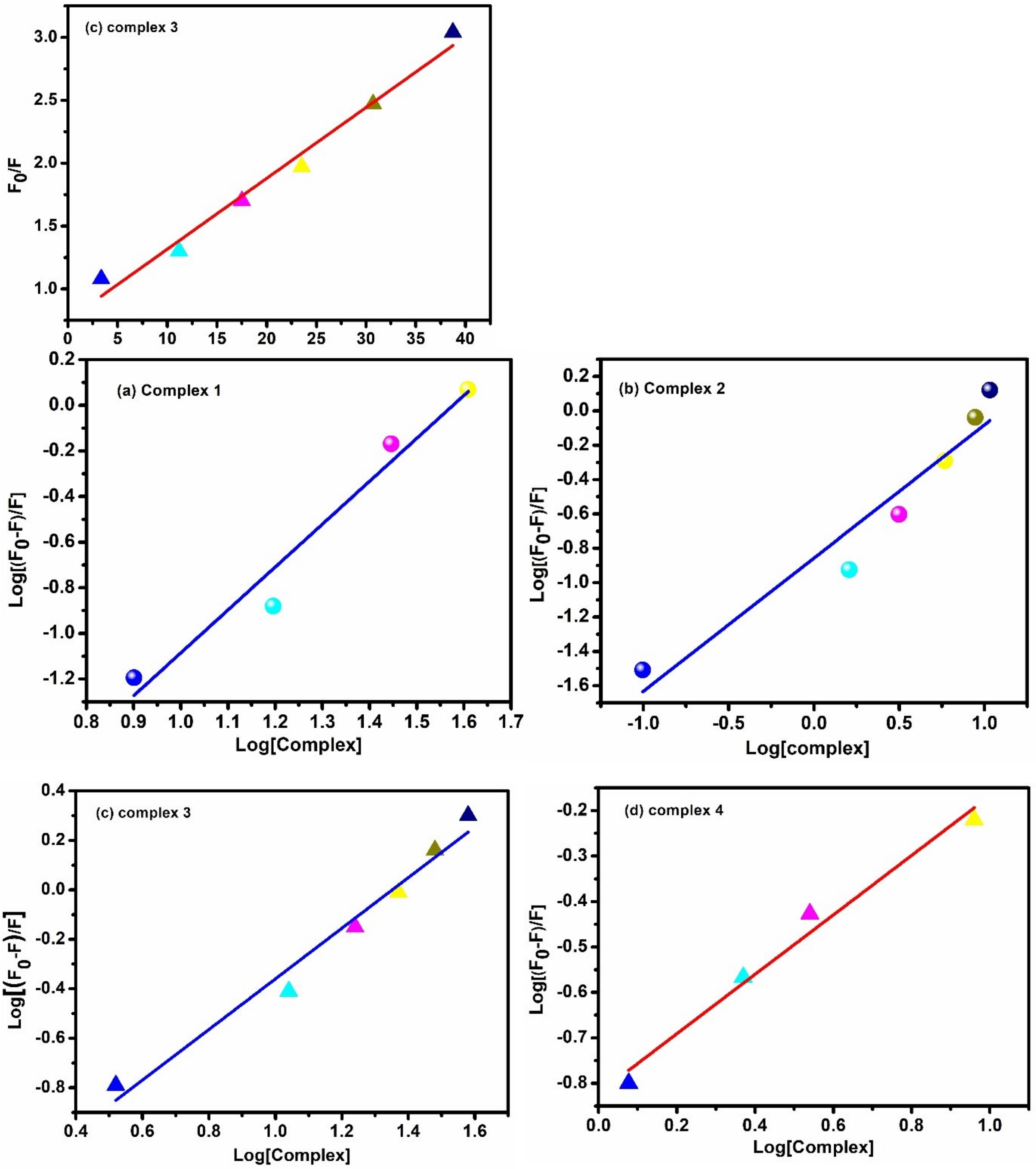

Fig. S43 Scatchard plots for the interaction of complexes with BSA binding system (a) complex 1 (b) complex 2 (c) complex 3 (d) complex 4

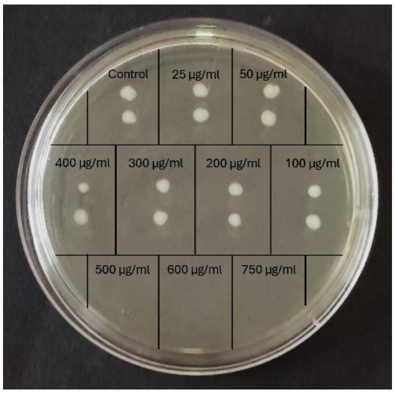

(A)

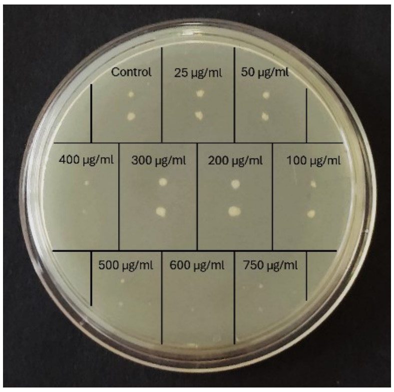

(B)

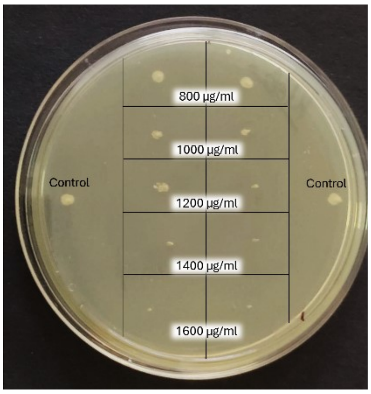

(C)

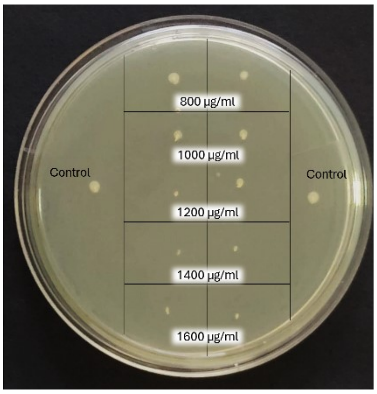

(D)

Fig. S44 MBC determination of complex 1 and complex 2 against KP and MRSA. The MBC of complex 1 against **(A)** KP and **(B)** MRSA was noted to be 500 μg/mL and 400 μg/mL, respectively. The MBC of complex 2 against **(C)** KP and **(D)** MRSA was documented to be 1400 μg/mL and 1200 μg/mL, respectively.

Table S1 Crystal data and refinement parameters of complex 2

Empirical Formula	[$C_{19}H_{26}ClN_2SRu^+$] (complex 2)
M_w	595.97
Crystal system	orthorhombic
Space group	$P2_12_12_1$
a / [Å]	9.9070(3)
b / [Å]	13.1110(3)
c / [Å]	18.3887(5)
α / [º]	90
β / [º]	90
γ / [º]	90
V [Å^3]	2388.52(11)
Z	4
D_c [Mg m^{-3}]	1.657
μ / [mm^{-1}]	0.979
F(000)	1200
Crystal size [mm^3]	0.68 x 0.59x 0.50
θ range for data collection (°)	3.817-26.418
Index ranges	-12≤*h*≤12, -16≤*k*≤16, -22≤l≤22
Reflections collected	27473
Unique reflections, [R_{int}]	4821[0.0353]
Final *R* indices	
R_1, wR_2 [I>2σI]	0.0184, 0.0493 [4766]
R_1, wR_2 (all data)	0.0187, 0.0497

Data/restraints/ parameters	4821/0/290
Goodness-of-fit on F^2	1.075

Table S2 Bond distances (Å) for complex 2

Bond lenghts			
Ru1-N1	2.101(2)	Ru1-C13	2.211(3)
Ru1-Cl1	2.3903(3)	Cu1-C14	2.205(3)
Ru1-S1	2.3393(7)	Cu1-C15	2.236(3)
Ru1-C11	2.186(3)	Cu1-C16	2.206(3)
Ru1-C12	2.228(3)		

Table S3 Hydrogen bond dimensions for Ru(II) complex 2

**D-H···A**	**H···A/ Å**	**D···A/ Å**	**D-H···A/º**
N2-H2…F1 [*1.5-x, 1-y,0.5+z*]	2.52(4)	3.346(5)	155(4)
N2-H2…F3 [*1.5-x, 1-y,0.5+z*]	2.45(5)	3.240(4)	150(3)
C3-H3…F5 [*0.5-x, 1-y,0.5+z*]	2.53	3.377(5)	151
C6-H6B…Cl1	2.68	3.342(4)	126
C13-H13…F1 [-1+*x, y,z*]	2.50	3.169(4)	129
